# γδ T cell receptor dependencies define a unique immunosurveillance modality

**DOI:** 10.1038/s41586-026-11076-4

**Published:** 2026-09-30

**Authors:** Nicolas Veland, Bethania Garcia-Cassani, Ángela Zarco-Cuadrillero, Annamaria Mavrigiannaki, Ambra Natalini, Josephine Eum, Alejandro Suarez-Bonnet, Pierre Vantourout, Duncan R. McKenzie, Jannik Franken, Ana V. Marin, Anett Jandke, Rosa Andrés-Ejarque, Jessica Strid, Adrian C. Hayday, Miguel Muñoz-Ruiz

**Affiliations:** 1 Peter Gorer Department of Immunobiology, King’s College London, London, UK; 2 Immunosurveillance Laboratory, The Francis Crick Institute, London, UK; 3 Development and Homeostasis of the Nervous System Laboratory, The Francis Crick Institute, London, UK; 4 Department of Immunology, Ophthalmology and Ear, Nose and Throat, Complutense University School of Medicine and 12 de Octubre Health Research Institute (imas12), Madrid, Spain; 5 Experimental Histopathology, The Francis Crick Institute, London, UK; 6 Department of Pathobiology and Population Sciences, Royal Veterinary College, Hatfield, UK; 7 Departement Biomedizin, University of Basel, Basel, Switzerland; 8 CRUK City of London Cancer Centre, King’s College London, London, UK; 9 Department of Immunology and Inflammation, Imperial College, London, UK; 10 Centre for Inflammation Biology and Cancer Immunology, King’s College, London, UK; 11 Present Address: CBMSO, CSIC-UAM, Institute for Molecular Biology-IUBM, UAM, Madrid, Spain

**Keywords:** T-cell receptor, Mucosal immunology

## Abstract

γδ T cells are one of three lymphocyte lineages that utilize gene rearrangement to diversify their antigen receptors. Nonetheless, the cells’ classification has remained uncertain, complicating our ability to understand the basis for their evolutionary conservation. Whereas many γδ T cells display hallmarks of adaptive immunity, others, including those in barrier tissues, make rapid, reportedly T cell receptor-independent responses that phenocopy innate immune cells^1^. Here we address this paradox and show that the phenotypes of tissue-intrinsic γδ T cells, including their rapid, innate-like responsiveness to tissue stress and carcinogenesis, acutely depend on the γδ T cell receptor (TCRγδ). Those dependencies emphasize the unique biology of γδ T cells and of the immunosurveillance modalities they mediate, with their clinical deployment evidently requiring environments conducive to TCRγδ signalling.

## Abstract (web summary)

Tissue-resident γδ T cells mount rapid, innate-like stress responses that require γδ T cell receptor signalling, revealing lineage-specific receptor dependence that underpins their conserved roles in immunosurveillance and informs therapeutic deployment.

## Main

In seeking to broaden the reach of immunotherapy, much interest is focused on immunosurveillance by human γδ T cells, particularly those that are enriched in tissues housing solid tumours or metastases^2–4^. Indeed, frequencies and activation states of human, tissue-associated γδ T cells commonly segregate with favourable patient outcomes^5–9^.

Some such γδ T cells manifest adaptive immunity, with durably expanded clones expressing diverse TCRγδ receptors^1,10–12^ that recognize self-encoded proteins qualitatively altered and/or overexpressed on infected or transformed cells^1,12^. Conversely, several γδ T cell subsets, including those intrinsic to tissues, manifest innate immunity by using cytokine and natural killer receptors (NKRs) to respond rapidly and non-clonally to stress-induced alarmins^1,13–16^. Indeed, functional, tissue-associated γδ T cells in human breast cancers lacked overt clonal expansions^5^. Moreover, several immunopathologies in mouse originate in IL-17-producing γδ T cells responding rapidly to IL-1β and IL-23 released within damaged tissues, seemingly independent of the T cell receptor (TCR)^16–18^.

In addressing whether γδ T cells are adaptive or innate, it has been hypothesized that TCRγδ may, during the cells’ development, licence them to function thereafter as innate immune cells^15^. Indeed, mouse epidermal and intestinal γδ T cells and their human colonic counterparts display TCR-dependent repertoire selection by tissue-specific, epithelial, butyrophilin-like (BTNL) molecules^19–22^, the importance of which is inferred from human disease associations of BTNL polymorphisms^22,23^.

Following development, sustained BTNL activity is required for some part of the steady-state phenotypes of epidermal and intestinal intraepithelial γδ T cells^19,24^. Nonetheless, to our knowledge, a real-time requirement for TCRγδ has not been investigated, particularly for myriad γδ T cells that are BTNL-independent—for example, human Vδ1^+^ cells and mouse Vγ6^+^ and Vγ4^+^ cells, which exist in several tissues and for which strong TCR engagement reportedly antagonizes the cells’ development^25^. Here we demonstrate that acute TCR ablation alters the steady-state phenotypes of all BTNL-dependent and BTNL-independent γδ T cell subsets that we examined and profoundly impairs the responses to inflammatory agents and cancers that are the hallmarks of γδ T cell biology in vivo. Thus, immunosurveillance by γδ T cells is unique, which is likely to be pertinent to the cells’ evolutionary conservation.

## Targeting tissue-intrinsic γδ cells

**Fig. 1:**
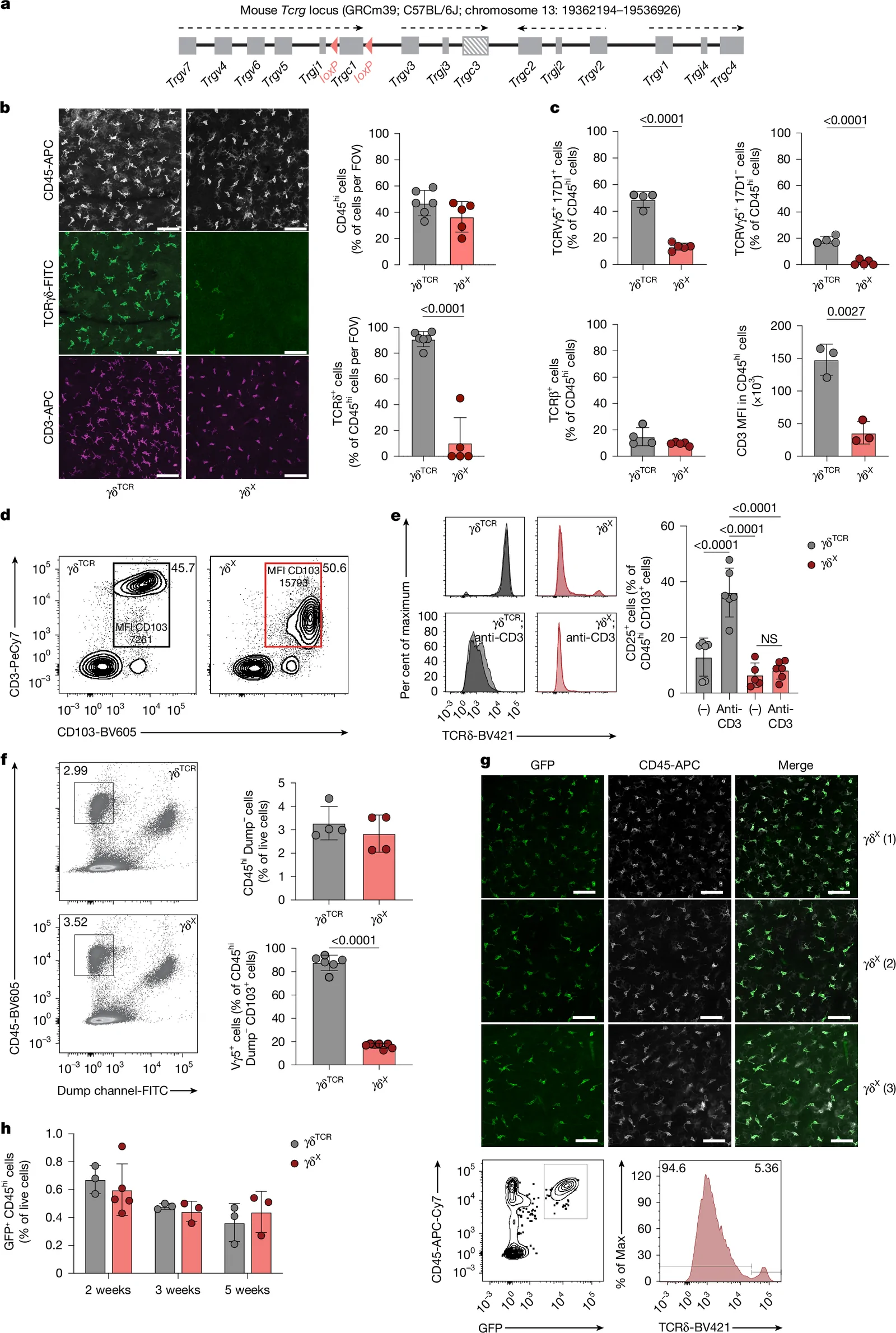
Effect of acute TCR Cγ1 deletion on skin γδ T cells. **a**, The mouse TCRγ-encoding locus. Introduced *loxP* sites (pink arrows) flank *Trgc1*, which is normally spliced to produce *Trgv7*, *Trgv4*, *Trgv6* or *Trgv5* (encoding Vγ7, Vγ4, Vγ6 or Vγ5, respectively) after recombination to *Trgj1* (encoding Jγ1). Hatched shading of *Trgc3* denotes it as a pseudogene. Arrows show the direction of transcription. **b**, Confocal micrographs of whole-mount ear skin stained for CD45, TCRγδ and CD3 in γδ^TCR^ (*n* = 6) and γδ^X^ (*n* = 5) mice, 1 week post-tamoxifen. Bars charts indicate CD45^hi^ and TCRδ^+^ cells as percentage of cells per field of view (FOV). **c**, Flow cytometry quantification of indicated populations 1 week post-tamoxifen, as percentages of CD45^hi^ epidermal cells in γδ^TCR^ (*n* = 4) and γδ^X^ (*n* = 5) mice, and CD3 expression in γδ^TCR^ and γδ^X^ mice (*n* = 3 per genotype). MFI, mean fluorescence intensity. **d**, Flow cytometry analysis of CD3 and CD103 expression on live epidermal cells from γδ^TCR^ and γδ^X^ mice 1 week post-tamoxifen. **e**, Flow cytometry analysis of TCR downregulation following overnight culture of cells from γδ^TCR^ (*n* = 6) or γδ^X^ (*n* = 6) mice 1 week post-tamoxifen with (bottom) or without (top) plate-bound anti-CD3, with associated CD25 upregulation (right) shown without (−) or with anti-CD3 stimulation. **f**, Representative gating (left) and quantification (right) of the CD45^hi^ Dump channel (F4/80^−^TCRβ^−^TCRVγ1^−^CD19^−^I-A/I-E^−^) epidermal cells from γδ^TCR^ (top, *n* = 4; bottom, *n* = 6) and γδ^X^ (top, *n* = 4; bottom, *n* = 7) mice, 2 months post-tamoxifen. **g**, Top, confocal micrographs of whole-mount ear skin showing GFP (green), CD45 (grey) and merged images from three γδ^X^ *Rosa26-mTmG*^*+/+*^ mice, 1 month post-tamoxifen. Representative flow cytometry plots show TCRδ expression (bottom right) after gating on epidermal CD45^+^GFP^+^ cells (bottom left) from a γδ^X^ *Rosa26-mTmG* mouse 1 month post-tamoxifen. **h**, Quantification of dermal GFP^+^CD45^+^ cells at the indicated time points post-tamoxifen in γδ^TCR^ (*n* = 3) or γδ^X^ (2 weeks, *n* = 5; others, *n* = 3) *Rosa26-mTmG*^*+/+*^ mice. Each dot represents an individual mouse; bars indicate mean ± s.d. s.d. *P* values determined with unpaired two-tailed *t*-tests (**b**,**c**,**f**,**h**) or two-way ANOVA with Šídák’s multiple comparisons test (**e**). Scale bars, 100 μm. Source data

To assess any real-time requirement for TCRγδ in vivo, we focussed on mouse tissue-intrinsic γδ T cells because they are broadly considered to be innate. They include two BTNL-selected, IFNγ-skewed subsets: TCRVγ5-expressing dendritic epidermal T cells (DETCs) and TCRVγ7-expressing intestinal intraepithelial lymphocytes (IELs), the latter being conserved in humans; as well as IL-17-skewed subepithelial TCRVγ6^+^ and TCRVγ4^+^ cells, which are found in many tissues including the dermis and for which no selecting element is known^1,20–22^. All such cells can be collectively targeted by deleting TCRCγ1 (*Trgc1*) (Fig. 1a). Although this should also affect systemic Vγ4^+^ T cells, systemic cells that express TCRVγ1-Cγ4, or, rarely, TCRVγ2-Cγ2 will be unaffected (Fig. 1a), limiting potential secondary effects of global γδ T cell impairment.

**Extended Data Fig. 1:**
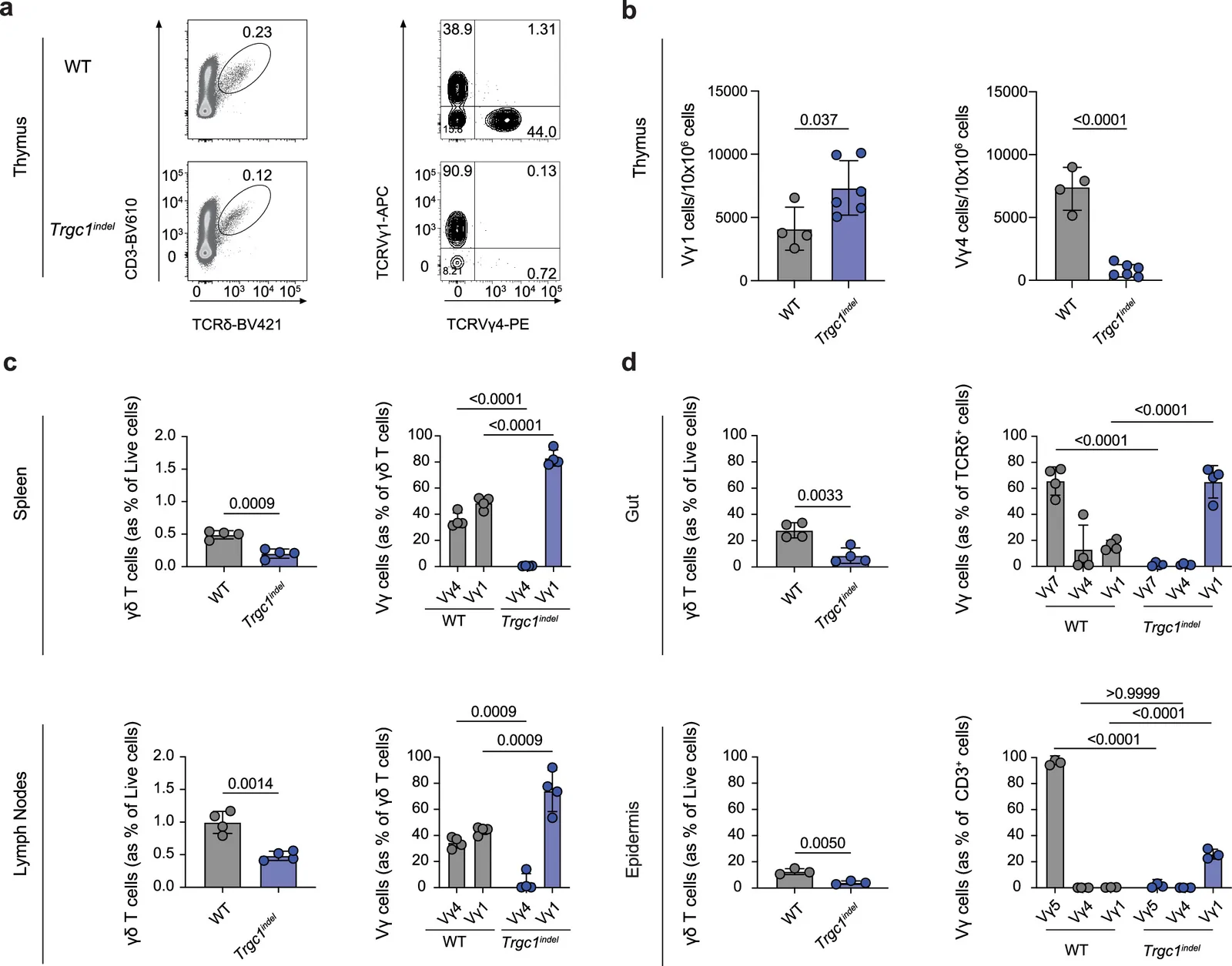
Experimental analysis of TCR deletion and γδ T cell dynamics. **a**, Flow cytometry analysis of Vγ1 and Vγ4 usage in TCRγδ^+^ thymocytes from wild-type (WT) and *Trgc1*^indel^ mice. **b**, Flow cytometry analysis of Vγ1^+^ and Vγ4^+^ TCRγδ^+^ thymocytes numbers from WT (*n* = 4) and *Trgc1*^indel^ (*n* = 6) mice. **c**, Flow cytometry analysis of the proportions of TCRγδ^+^ (relative to live cells, left column), and Vγ1^+^ or Vγ4^+^ (relative to total γδ T cells, right column) in splenocytes (upper row) and mesenteric lymph node cells (lower row) from WT (*n* = 4) and *Trgc1*^indel^ (*n* = 4) mice. **d**, Flow cytometry analysis of the proportions of TCRγδ^+^ (relative to live cells, left column), and Vγ7^+^ (or Vγ5^+^), Vγ4^+^ or Vγ1^+^ (relative to total γδ T cells or CD3^+^ cells, right column) in gut (upper row) and epidermis (lower row) from WT (gut *n* = 4, epidermis *n* = 3) and *Trgc1*^indel^ (gut *n* = 4, epidermis *n* = 3) mice. Each dot represents an individual mouse; bars indicate mean ± SD. *p*-values were determined with unpaired two-tailed *t*-test or two-way ANOVA. Source data

First we generated a mouse strain, *Trgc1*^indel^, carrying an internal *Trgc1* deletion (Supplementary Methods). Adult *Trgc1*^indel^ mice displayed approximately half the normal number of thymic and systemic γδ T cells, which mostly expressed Vγ1-Cγ4 (Extended Data Fig. 1a–c), and their intestines and epidermis were depleted of signature Vγ7^+^ and Vγ5^+^ cells, which were variably replaced by Vγ1-Cγ4^+^ cells and TCRVγ^−^ CD3^+^ cells (that is, αβT cells) (Extended Data Fig. 1d). With the practicality of selectively targeting *Trgc1* thereby established, we proceeded to inducibly deplete *Trgc1* from mature γδ T cell compartments.

## TCRγδ-depleted cells survive in tissues

A floxed allele, *Trgc1*^*fl*^, was engineered (Fig. 1a) and *Trgc1*^*fl/fl*^ mice were crossed to strains expressing tamoxifen-dependent Cre recombinase expressed from the γδ T cell-specific *Tcrd* promoter, or the *Rosa26* promoter to increase the penetrance of *Trgc1* deletion in the gut. Typically, gene deletion was achieved by administering tamoxifen over 7 consecutive days, followed by at least 7 days of rest to permit TCR protein loss.

**Extended Data Fig. 2:**
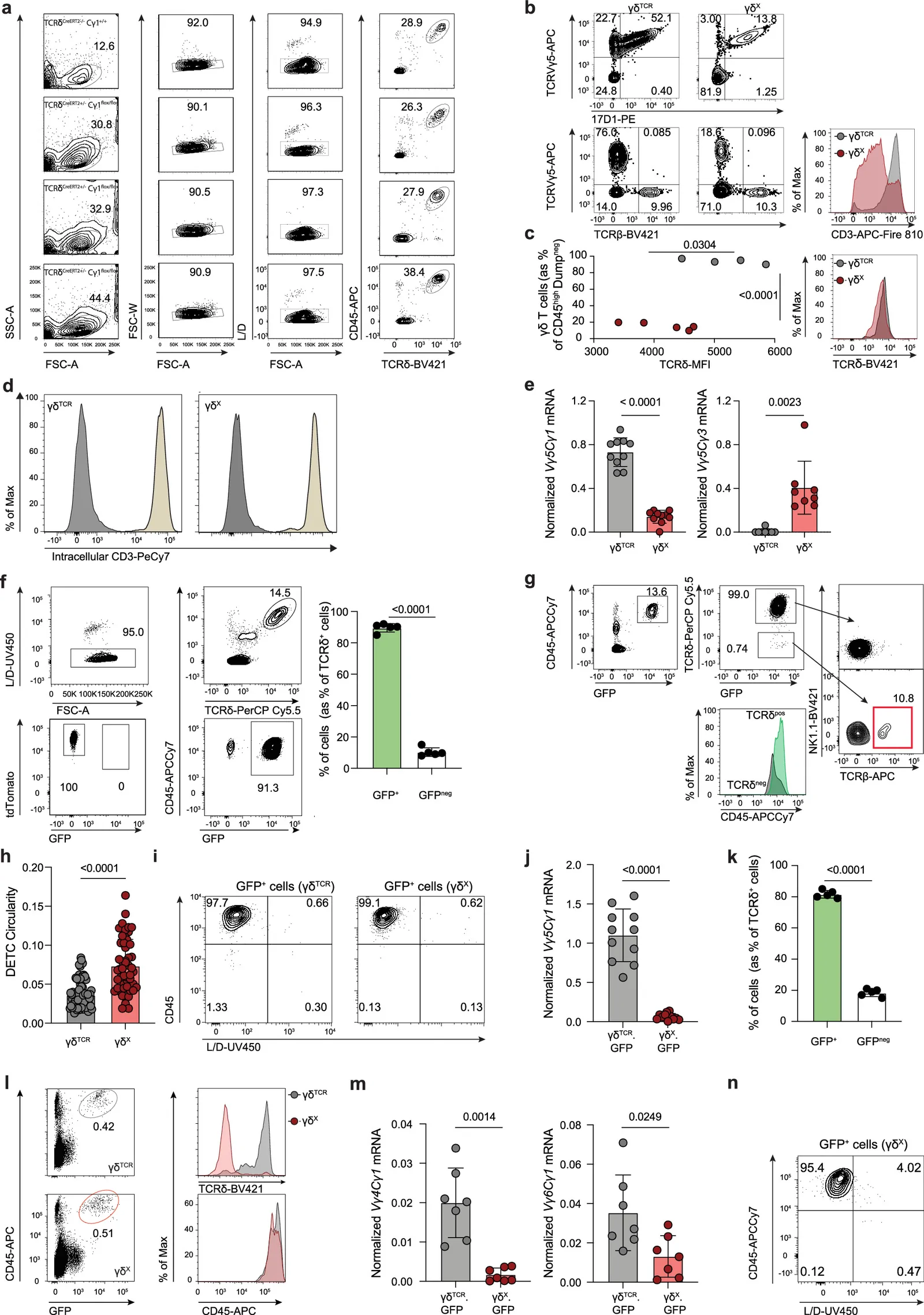
Impact of acute TCR Cγ1 deletion on skin γδ T cells. **a**, Gating strategy for identifying viable γδ T cells in the epidermis of TCRδ^*CreERT2*–/–^Cγ1^+/+^ (first row) and TCRδ^*CreERT2*+/–^Cγ1^fl/fl^ (second to fourth rows) mice, highlighting comparable frequencies of CD45^high^ TCRδ^+^ cells. **b**, Flow cytometry analysis of epidermal immune cell populations in γδ^TCR^ and γδ^X^ mice, 1-week post-tamoxifen. Representative contour plots show TCRVγ5^+^ cells versus 17D1^+^ (upper left) or versus TCRβ^+^ (lower left) after gating on CD45^high^ cells; overlayed histograms (lower right) show CD3 expression levels. **c**, Analysis of residual epidermal TCRγδ^+^ cells 1-month post-tamoxifen. Scatterplot (left) shows frequencies of γδ T cells as percentage of CD45^high^Dump^neg^ cells (y-axis) compared to TCRδ MFI (x-axis) from γδ^TCR^ (*n* = 4) and γδ^X^ (*n* = 5) mice. Each dot represents an individual mouse. Overlayed histograms (right) show representative TCRδ expression in γδ^TCR^ versus γδ^X^ mice. **d**, Representative intracellular CD3 expression levels (beige histograms) in epidermal CD45^high^ cells from γδ^TCR^ and γδ^X^ mice, 1-week post-tamoxifen. Grey histograms, negative control staining. **e**, RT-qPCR quantification of Vγ5Cγ1- and Vγ5Cγ3-encoding mRNAs in epidermal cells from γδ^TCR^ (*n* = 10) and γδ^X^ (*n* = 10) mice 1-week post-tamoxifen, normalized to Cyclophilin A (*CypA*). **f**, Flow cytometry gating strategy (contour plots) for the analysis of live (top left) epidermal cells from *Rosa26.mTmG.TCRδ*^*CreERT2*^*Trgc1*^+/+^ mice assessed for tdTomato and GFP expression (lower left) pre-tamoxifen. CD45^high^ TCRγδ^+^ cells from total live cells (top right) were analysed for GFP conversion (bottom right) 1-month post-tamoxifen, quantified as frequencies of CD45^high^ TCRγδ^+^ cells (bar chart, *n* = 5). **g**, Flow cytometry analysis of TCRβ (upper right contour), NK1.1 (lower right contour), and CD45 expression (overlayed histograms) in TCRδ^+^ and TCRδ^neg^ cells from *Rosa26.mTmG*-γδ^TCR^ mice 1-month post-tamoxifen. **h**, Confocal analysis of DETC circularity in whole-mount skin from γδ^TCR^ (*n* = 74) and γδ^X^ (*n* = 51) mice 2-weeks post-tamoxifen. **i**, Representative flow cytometry analysis of epidermal CD45^+^ GFP^+^ viability in lymphocytes from *Rosa26.mTmG*-γδ^TCR^ (left) and *Rosa26.mTmG*-γδ^X^ (right) mice 2-weeks post-tamoxifen. **j**, RT-qPCR quantification of Vγ5Cγ1-encoding mRNAs in GFP^+^ epidermal cells from *Rosa26.mTmG*-γδ^TCR^ (*n* = 11) and *Rosa26.mTmG*-γδ^X^ (*n* = 11) mice 1-week post-tamoxifen, normalized to *CypA*. **k**, Flow cytometry analysis of GFP conversion in dermal CD45^+^TCRγδ^+^ from *Rosa26.mTmG*-γδ^TCR^ (*n* = 5) mice 1-week post-tamoxifen. **l**, Flow cytometry analysis of dermal CD45^+^GFP^+^ cells from *Rosa26.mTmG*-γδ^TCR^ (upper contour plot) and *Rosa26.mTmG*-γδ^X^ (lower contour plot) mice for surface TCRδ (upper histograms) and CD45 (lower histograms) expression 1-week post-tamoxifen. **m**, RT-qPCR quantification of Vγ4Cγ1- and Vγ6Cγ1-encoding mRNAs in GFP^+^ dermal cells from *Rosa26.mTmG*-γδ^TCR^ (*n* = 7) and *Rosa26.mTmG*-γδ^X^ (*n* = 7) mice 1-week post-tamoxifen, normalized to *CypA*. **n**, Representative flow cytometry analysis of dermal CD45^+^GFP^+^ viability in lymphocytes from *Rosa26.mTmG*-γδ^X^ mice 2-weeks post-tamoxifen. Each dot represents an individual mouse except in **h** (individual cells); bars indicate mean ± SD; *p*-values determined with unpaired two-tailed *t*-test. Source data

We first confirmed that Cre recombinase expression was not leaky, prior to tamoxifen treatment, by observing that frequencies of epidermal TCRγδ^+^ cells expressing CD45 at high levels (a DETC signature trait) were comparable in untreated *Tcrd-creERT2*^*+/−*^* Trgc1*^*fl/fl*^ mice and control *Tcrd-creERT2*^*−/−*^* Trgc1*^+/+^ littermates (Extended Data Fig. 2a). Thus, we proceeded to compare mice in which Cγ1 was inducibly deleted (γδ^X^ mice) with tamoxifen-treated control mice (γδ^TCR^ mice)—that is, *Trgc1*^*fl/fl*^ mice lacking Cre, or *cre*^*+*^* Trgc1*^+/+^ mice lacking floxed alleles.

Fourteen days after starting 7 days of tamoxifen treatment, we observed comparable frequencies of epidermal CD45^hi^ cells in γδ^X^ and γδ^TCR^ mice (note, CD45^low^ cells are Langerhans cells), but CD45^hi^ cells in γδ^X^ mice were mostly TCRδ^−^ (Fig. 1b). Residual TCRγδ-expressing cells in γδ^X^ mice were mostly Vγ5^+^, stained with 17D1, an antibody specific at steady state for the Vγ5^+^ DETC TCR, and displayed moderate to neglible TCR levels, presumably reflecting variable Cre penetrance (Fig. 1c and Extended Data Fig. 2b,c).

By contrast, most CD45^hi^ cells in γδ^X^ mice were TCRβ^−^, expressed surface CD3 at low levels, but expressed intracellular CD3 at levels comparable to γδ^TCR^ DETCs (Fig. 1b–d and Extended Data Fig. 2d), phenocopying immature thymocytes prior to TCR expression^26^. They also appeared less dendritic than normal (Fig. 1b), phenocopying DETCs dysregulated by transient, antibody-mediated blockade of SKINT1, the TCR-dependent, BTNL-selecting element for DETCs^24^. We conclude that these are TCR-ablated DETCs that conspicuously retained CD103, a marker of tissue-resident T cells, at slightly higher levels than γδ^TCR^ DETCs (Fig. 1d). As anticipated, they expressed significantly reduced Vγ5-Cγ1 transcripts, with Vγ5 RNAs now spliced to Cγ3, a pseudogene^27^ constituting a biologically neutral marker of Cγ1 deletion (Fig. 1a and Extended Data Fig. 2e).

Whereas residual TCRγδ-expressing cells in γδ^X^ mice displaying TCR levels resembling those of control DETCs responded to cell-activating CD3-reactive antibodies by TCR downregulation, epidermal γδ^X^ cells en masse showed no functional response to anti-CD3, as assessed by high-affinity IL-2 receptor (CD25) upregulation (Fig. 1e). Thus, TCRγδ had been physically and functionally ablated from most mature DETCs within 1 week of tamoxifen withdrawal.

To assess the survival of TCR-ablated cells, we tracked cells failing to stain in a ‘Dump’ channel comprising antibodies for TCRβ, TCRVγ1 (systemic γδ cells), major histocompatibility complex (MHC) class II (to detect Langerhans cells), F4/80 (to detect macrophages) and CD19 (to detect B cells) (Fig. 1f). Notwithstanding a slight downward trend, CD45^hi^Dump^−^ cells were comparably abundant in γδ^X^ and γδ^TCR^ mice for more than 2 months after tamoxifen treatment, and any residual TCRγδ-expressing DETCs remained TCRVγ5^+^ rather than reflecting heterologous T cell infiltration (Fig. 1f).

To study cells deprived of their defining marker, TCRγδ, we next crossed *Tcrd-creERT2* strains to *Rosa26-mTmG* mice in which cells activating Cre shift from GFP^−^Tomato^+^ to GFP^+^^28^. Validating this, more than 90% of epidermal CD45^hi^TCRγδ^+^ cells in *Tcrd-creERT2*^*+/−*^* Trgc1*^*+/+*^ mice became GFP^+^ within 1 week of tamoxifen treatment, and remained so for at least 1 month thereafter (Extended Data Fig. 2f). Moreover, more than 99% of GFP^+^ cells were CD45^hi^TCRδ^+^ and of the remaining 1%, only about 10% expressed TCRβ and none expressed NK1.1 (identifying natural killer (NK) and natural killer T (NKT) cells) (Extended Data Fig. 2g).

With the mTmG system validated, we examined *Tcrd-creERT2*^*+/*−^*;Rosa26-mTmG Trgc1*^*fl/fl*^ mice, observing, for at least 4 weeks post-tamoxifen treatment, CD45^hi^ GFP^+^ cells evenly distributed across the epidermis and somewhat rounded, as confirmed by quantitative morphometrics (Fig. 1g and Extended Data Fig. 2h). By live/dead staining, γδ^X^ epidermal GFP^+^ cells showed comparable viability to γδ^TCR^ GFP^+^ cells, and their TCR-ablated status was confirmed by the near-absence of Vγ5-Cγ1 transcripts (Extended Data Fig. 2i,j).

In *Tcrd-creERT2;Rosa26-mTmG Trgc1*^*+/+*^ mice, more than 80% of dermal TCRγδ^+^ cells converted to GFP^+^ (Extended Data Fig. 2k). Similar populations of dermal CD45^hi^GFP^+^ cells were observed in γδ^X^ mTmG mice, but they were almost all TCRδ^−^ and expressed significantly reduced Vγ4-Cγ1 and Vγ6-Cγ1 mRNA levels (Extended Data Fig. 2l,m). The CD45^hi^GFP^+^TCRδ^−^ cells were evidently viable (Extended Data Fig. 2n), and were comparably represented to TCRδ^+^GFP^+^ cells in γδ^TCR^ mTmG mice, with both strains showing gradual declines that typically follow perinatal maturation (Fig. 1h). In sum, the maintenance of dermal or epidermal γδ cells for at least several weeks was negligibly affected by acute conversion from a TCR^+^ state to a TCR^−^ state.

## Phenotypes of TCRγδ-depleted cells

**Fig. 2:**
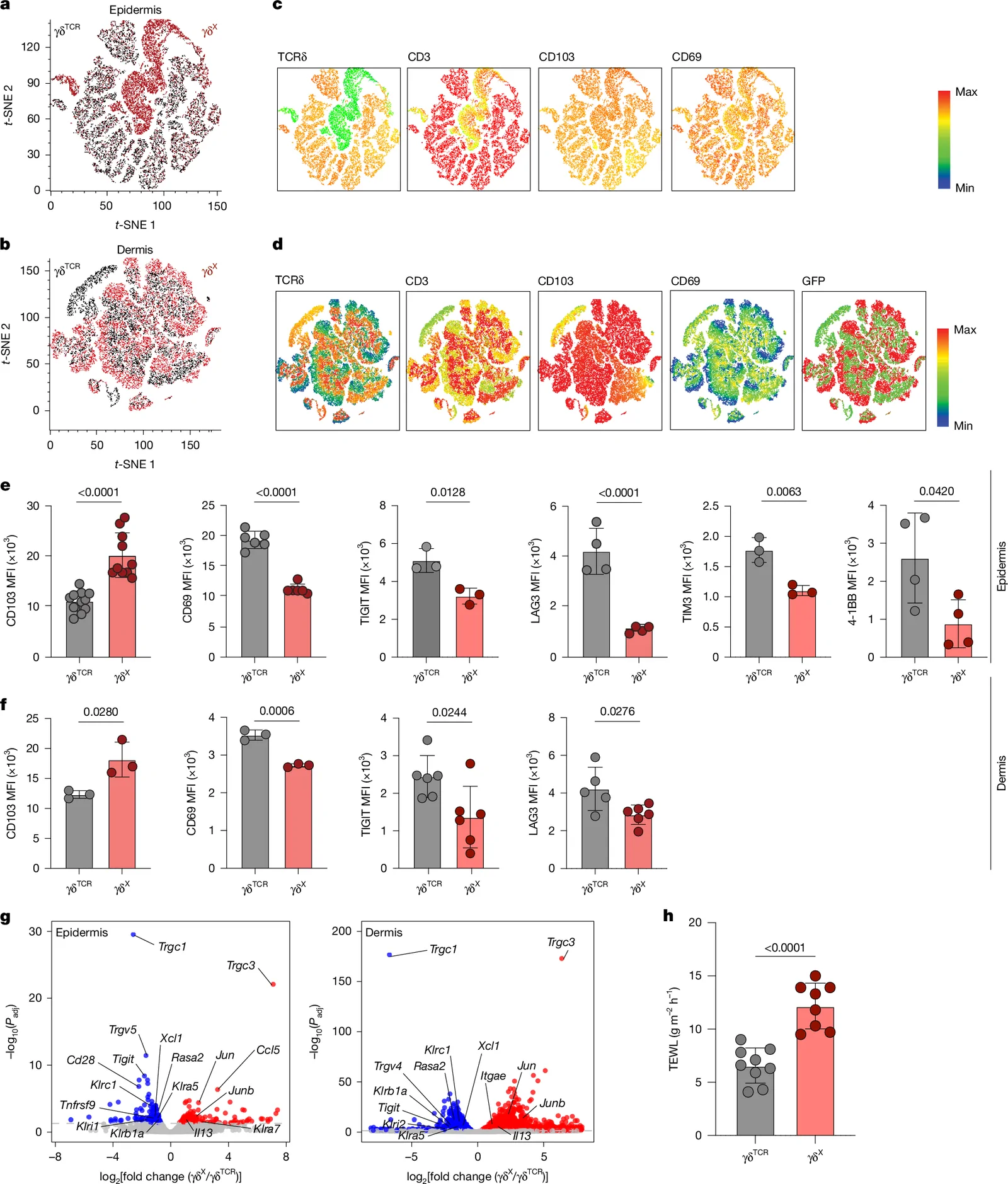
Phenotypic and physiologic differences in skin of γδ^X^ mice. **a**–**d**, Spectral flow cytometry analysis of epidermal CD45^+^Dump^−^ cells from γδ^TCR^ (*n* = 2) and γδ^X^ (*n* = 2) mice (**a**) 1 week post-tamoxifen, and dermal TCRδ^+^ cells from γδ^TCR^ mice (*n* = 4) and GFP^+^ cells from γδ^X^ mice (*n* = 5) (**b**) 1 week post-tamoxifen. *t*-distributed stochastic neighbour embedding (*t*-SNE) was performed on concatenated γδ^TCR^ and γδ^X^ datasets using 10,000 iterations, incorporating all fluorescent parameters except those used for gating, viability or directly associated with the TCR (CD3, TCRδ and TCRαβ). Relative expression of key markers is shown on concatenated *t*-SNE plots for epidermal (**c**) and dermal (**d**) cells. Max, maximum; min, minimum. **e**, Quantification of expression for surface markers in epidermal CD45^+^Dump^−^ cells. For CD103, γδ^TCR^ (*n* = 11) versus γδ^X^ (*n* = 10); CD69, γδ^TCR^ (*n* = 6) versus γδ^X^ (*n* = 6); TIGIT, γδ^TCR^ (*n* = 3) versus γδ^X^ (*n* = 3); LAG3, γδ^TCR^ (*n* = 4) versus γδ^X^ (*n* = 4); TIM3, γδ^TCR^ (*n* = 3) versus γδ^X^ (*n* = 3); 4-1BB, γδ^TCR^ (*n* = 4) versus γδ^X^ (*n* = 4). **f**, Same as in **e**, for dermal GFP^+^ cells. For CD103, γδ^TCR^ (*n* = 3) versus γδ^X^ (*n* = 3); CD69, γδ^TCR^ (*n* = 3) versus γδ^X^ (*n* = 3); TIGIT, γδ^TCR^ (*n* = 6) versus γδ^X^ (*n* = 6); LAG3, γδ^TCR^ (*n* = 5) versus γδ^X^ (*n* = 6). **g**, Genes that were significantly downregulated (blue) or upregulated (red) in RNA-seq analysis of epidermal CD45^+^Dump^−^ (left) and dermal GFP^+^ (right) cells from γδ^X^ (*n* = 3) compared to γδ^TCR^ (*n* = 3) mice 1 week post-tamoxifen. *P*_adj_, adjusted *P* value. **h**, TEWL measured on ear skin from γδ^TCR^ (*n* = 9) and γδ^X^ (*n* = 8) mice 2 weeks post-tamoxifen. Each dot represents an individual mouse except in **g**, where each dot represents an individual gene. Bars indicate mean ± s.d. *P* values determined with unpaired two-tailed *t*-test except in **g** (Methods). Source data

**Extended Data Fig. 3:**
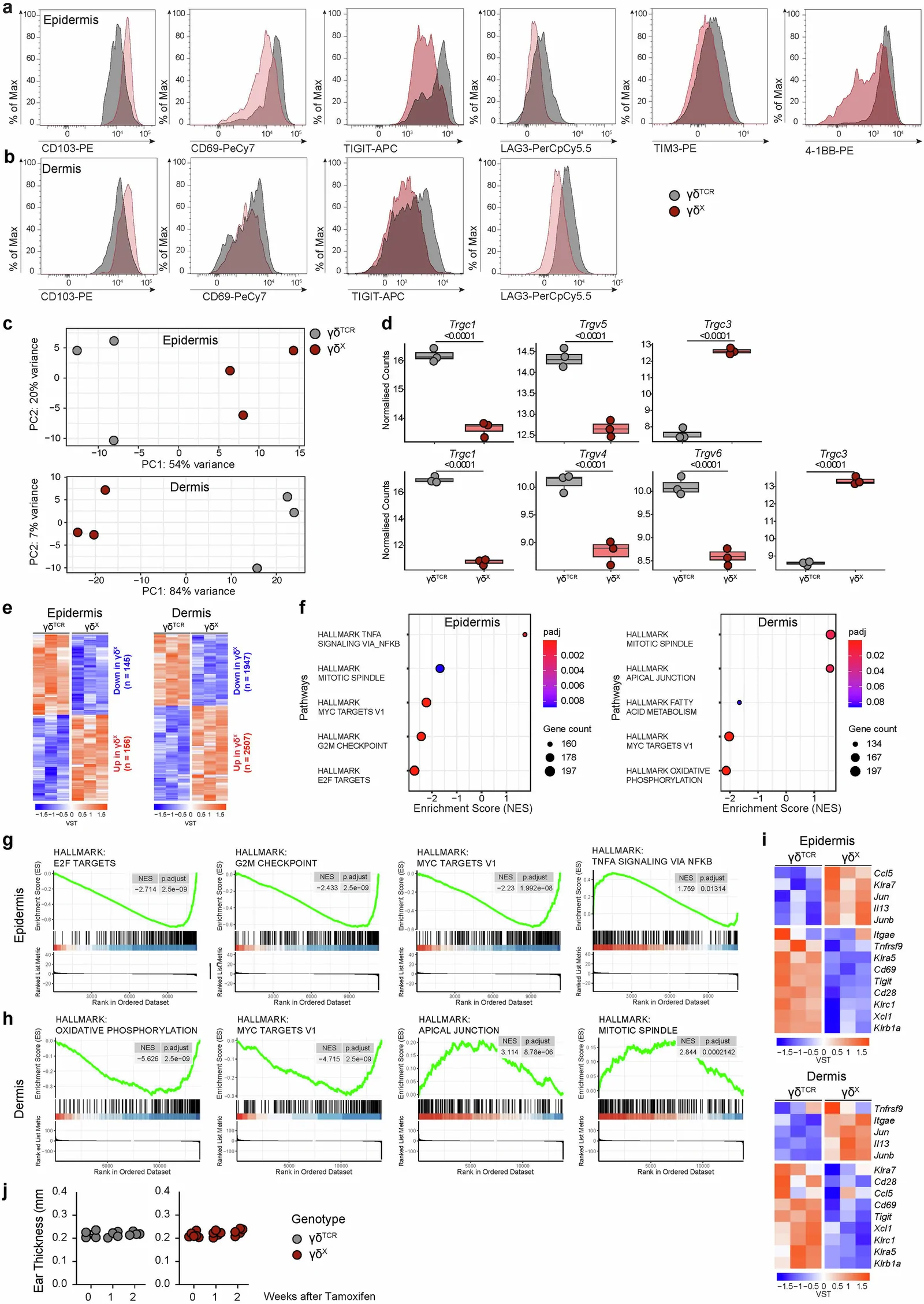
Phenotypic and physiologic differences in γδ^TCR^ and γδ^X^ skin. **a**,**b**, Representative flow cytometry analysis for the expression of the indicated proteins in epidermal CD45^+^Dump^neg^ (**a**) and dermal GFP^+^ (**b**) cells from γδ^TCR^ and γδ^X^ mice 1 week post-tamoxifen. **c*-*i**, RNA-seq data on epidermal and dermal cells from γδ^TCR^ (*n* = 3) and γδ^X^ (*n* = 3) mice (Fig. 2g) was examined by Principal Component Analysis (PCA) (**c**); expression levels of Variance Stabilised Transformation (VST)-normalised counts for the indicated mRNAs relating to TCR gene usage (**d**, boxes indicate median, interquartile range and minimum/maximum); heatmaps of VST-normalised counts for differentially expressed genes (DEG) downregulated (blue) or upregulated (red) in γδ^X^ compared to γδ^TCR^ cells (**e**); Gene set enrichment analysis (GSEA) of DEGs (**f**, top 5 sets ranked by normalised enrichment score (NES) using the MSigDB Hallmark gene sets, dot size and colour indicate number of genes and padj respectively); running enrichment score for the top 4 significantly enriched gene sets from MSigDB Hallmark gene sets based on NES values (**g**, **h**); and heatmaps of VST-normalised counts for the indicated genes upregulated (red) or downregulated (blue) in γδ^X^ compared to γδ^TCR^ cells (**i**). All adjusted *p*-values (padj) were determined with DESeq2 package using the Benjamini–Hochberg procedure (for details, see Methods). **j**, Ear thickness measurement in γδ^TCR^ (*n* = 4) and γδ^X^ (*n* = 6) mice at the indicated time points post-tamoxifen. Horizontal lines represent the mean for each condition. Each dot in **c**, **d** and **j** represents an individual mouse. Source data

Given that epidermal and dermal γδ T cells markedly differ developmentally, phenotypically and functionally, we used spectral flow cytometry to explore whether they were comparably affected by acute TCR deletion. Dump^−^ epidermal T cells and GFP^−^ dermal γδ T cells were predictably heterogeneous, and yet their counterparts from γδ^X^ mice largely segregated into distinct clusters, validated a posteriori by assessing surface TCRγδ and CD3 expression and (for dermal cells) GFP (Fig. 2a–d). Moreover, monitoring several important immunoregulatory proteins across independent cohorts of mice revealed that epidermal and dermal γδ T cells shared responses to acute TCRγδ depletion, namely, CD103 upregulation and CD69, TIGIT and LAG3 downregulation, added to which epidermal γδ^X^ cells acutely downregulated TIM3 and 4-1BB, with a loss of 4-1BB^hi^ cells, as occurred in SKINT1-blockaded mice^24^ (Fig. 2e,f and Extended Data Fig. 3a,b).

To further investigate TCR dependencies of skin γδ cells, we used transcriptomics. Previous application of single-cell RNA-sequencing (scRNA-seq) resolved steady-state DETC transcriptomes into four major subtypes^24^ but was not effective at distinguishing DETCs from unmanipulated and SKINT1-blockaded mice, largely because there were few differentially expressed genes (DEGs). Thus, we adopted bulk RNA sequencing (RNA-seq) to compare skin cells from γδ^X^ and γδ^TCR^ mice (Supplementary Table 1).

Using principal component analysis (PCA), transcriptomes completely segregated according to genotype in PC1 (which contains most of the variance) (Extended Data Fig. 3c) and, as expected, showed significant differences in *Trgc1* and *Trgv5* RNA counts (for epidermal cells) and *Trgc1*, *Trgv4* and *Trgv6* counts (for dermal cells) (Extended Data Fig. 3d). Also as anticipated, epidermal and dermal γδ^X^ cells showed significantly upregulated *Trgc3* expression (Extended Data Fig. 3d), which, via low-input, high-resolution proteomic analysis of TCR proteins, was confirmed to be a non-coding, and thus neutral, marker of γδ^X^ cells (Supplementary Methods and Supplementary Table 2).

Beyond TCR RNAs, epidermal γδ^X^ and γδ^TCR^ cells were segregated by only 145 downregulated and 156 upregulated DEGs with 11,723 unaffected genes, whereas dermal γδ^X^ cells displayed 1,947 downregulated and 2,507 upregulated DEGs, with 10,751 unaffected genes (Fig. 2g, Extended Data Fig. 3e and Supplementary Table 1). Thus, TCRγδ is an essential real-time regulator of gene expression in epidermal and dermal γδ T cells, albeit to different degrees.

Gene set enrichment analysis (GSEA) showed that epidermal γδ^X^ cells exhibited significant albeit weak dysregulation of genes classified as ‘TNF signalling via NF-κB’ (Extended Data Fig. 3f,g) These include RNAs encoding proteins implicated in maintaining mouse tissue-intrinsic γδ T cells in an activated-yet-resting state^29^ (such as BTG2, JUNB and FOS) (Fig. 2g). Substantially downregulated gene sets included ‘E2F target’, ‘G2M checkpoint’ and ‘MYC targets’, which were also among the most significantly downregulated in dermal γδ^X^ cells (Extended Data Fig. 3f–h). Epidermal γδ^X^ cells also downregulated ‘mitotic spindle’ genes, and although this gene set was overall upregulated in dermal γδ^X^ cells (Extended Data Fig. 3f,h), individual genes showed concordant patterns in epidermal and dermal γδ^X^ cells—for example, downregulation of *Rasa2* (Fig. 2g and Supplementary Table 3), loss of which improves CAR-T cell activation^30^. Dermal γδ^X^ cells also downregulated ‘oxidative phosphorylation’ genes (Extended Data Fig. 3f,h), which seems noteworthy given that these cells produce IL-17 and that oxidative phosphorylation is the major metabolic pathway of systemic IL-17-producing Vγ4^+^ cells^31^.

Some DEGs paralleled protein changes: for example, *Tigit* and *Cd69* downregulation in epidermal and dermal γδ^X^ cells, and dermal γδ^X^ cell upregulation of *Itgae* (encoding CD103α) (Fig. 2g and Extended Data Fig. 3i). Nonetheless, mRNA and protein expression do not always align^32^, and significant decreases in TIM3 and LAG3 surface expression were not consistently mirrored transcriptionally (Supplementary Table 1).

Epidermal and dermal γδ T cells also responded to acute TCR deletion by downregulating mRNAs encoding XCL1 (Fig. 2g and Extended Data Fig. 3i), a chemokine that mediates DETC-dependent recruitment of dendritic cells following cytomegalovirus infection^33^ and is implicated in the maintenance of CD8^+^ T cell competence following immune checkpoint therapy^34,35^. They also shared upregulation of *Jun* and *Junb*, encoding early-response transcription factors that regulate cellular activation states, as well as *Il13* (Fig. 2g and Extended Data Fig. 3i), which evokes upregulation of T helper 2 (T_H_2)-associated genes following nutrient deprivation^36^. Conspicuously, TCRγδ determined expression levels of RNAs encoding NKRs and killer inhibitory receptors (KIRs) that regulate innate immune responses. Thus, epidermal and dermal γδ^X^ cells shared significant downregulation of *Klrb1a* (encoding Ly55a), *Klra5* (encoding Ly49e) and *Klrc1* (encoding NKG2A), and variable dysregulation of *Klra7* (encoding Ly49g) (Fig. 2g and Extended Data Fig. 3i).

We next tested whether the effects of TCR ablation on skin γδ T cell phenotypes might have overt physiological consequences. Indeed, within two weeks of tamoxifen administration, γδ^X^ mice showed impaired epidermal integrity as judged by significantly increased trans-epidermal water loss (TEWL) (Fig. 2h), as occurs in γδ T cell-deficient mice and SKINT1-blockaded mice^24,37^. This did not reflect an induced pro-inflammatory state, because ear thickness was contemporaneously unaffected (Extended Data Fig. 3j).

## Distinct gut and skin TCRγδ dependences

**Fig. 3:**
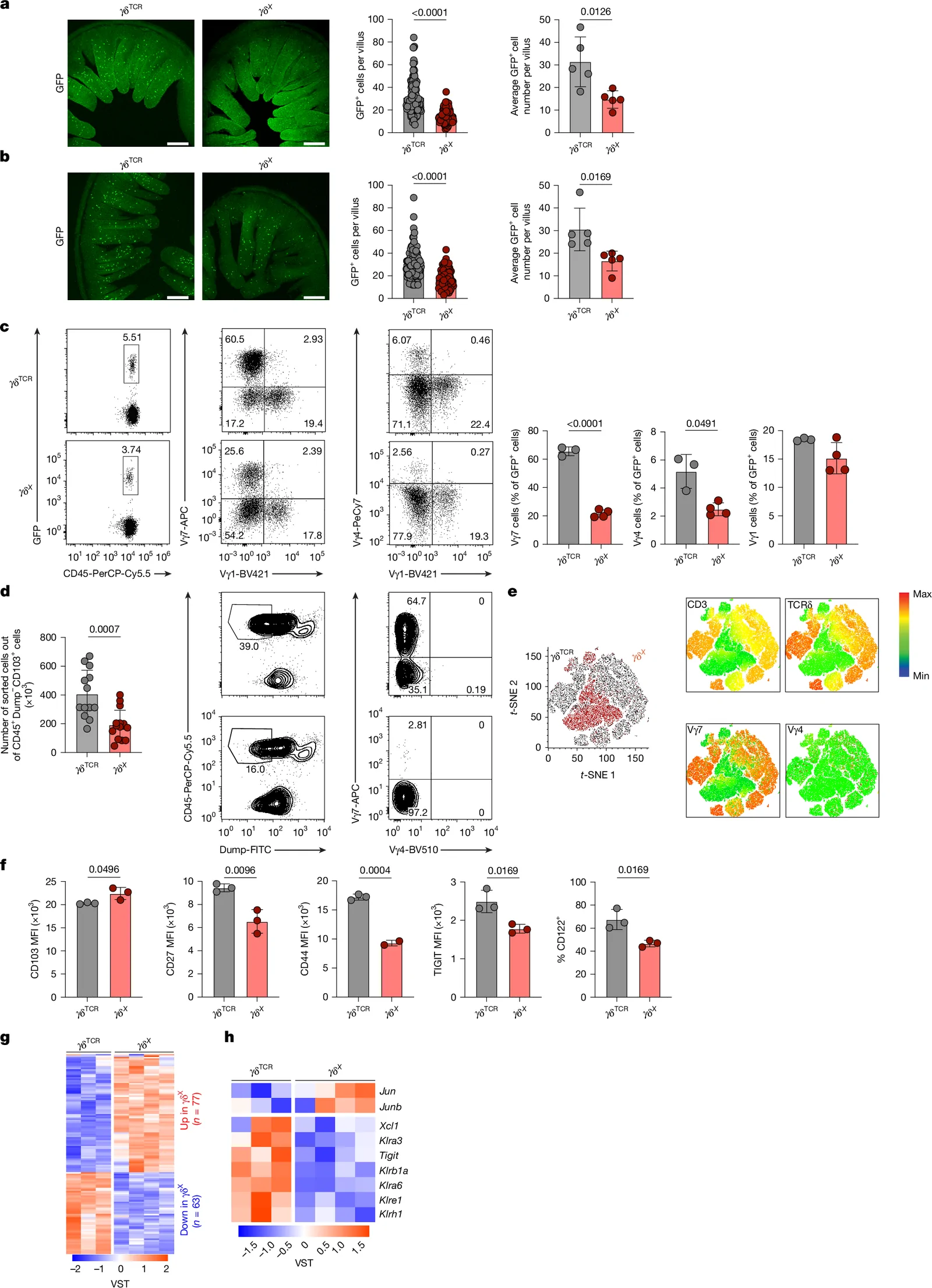
Phenotypic effect of TCRγδ deletion on small intestinal IELs. **a**,**b**, Left, representative confocal micrographs showing GFP^+^ lymphocyte distribution in 80-µm sections of small intestine from *Rosa26-mTmG*-γδ^TCR^ (*n* = 5) and *Rosa26-mTmG*-γδ^X^ (*n* = 5) mice at 2 (**a**) and 4 (**b**) weeks post-tamoxifen. Right, quantification of GFP^+^ cell numbers. Scale bars, 100 µm. **c**, Left, representative flow cytometry analyses of TCRVγ7^+^, Vγ4^+^ and Vγ1^+^ in GFP^+^ cells from small intestines of *Rosa26-mTmG*-γδ^TCR^ (*n* = 3) and *Rosa26-mTmG*-γδ^X^ (*n* = 4) mice 2 weeks post-tamoxifen. Right, quantification of indicated cell types. **d**, Flow cytometry analysis of the number of recovered CD45^+^Dump^−^CD103^+^ IELs (left) and their Vγ7 and Vγ4 usage (representative flow plots) in *Rosa26-creERT2*-γδTCR (*n* = 13) and *Rosa26-creERT2*-γδ^X^ (*n* = 15) mice 2 weeks post-tamoxifen. **e**, Spectral flow cytometry analysis of intestinal CD45^+^Dump^−^ cells from γδ^TCR^ (*n* = 3) and γδ^X^ (*n* = 3) mice 2 weeks post-tamoxifen. *t*-SNE was performed on concatenated γδ^TCR^ and γδ^X^ datasets using 10,000 iterations, incorporating all fluorescent parameters except those used for gating and viability, and those directly associated with the TCR (CD3, TCRδ, Vγ7 and Vγ4), relative expression levels of which are shown on concatenated *t*-SNE plots, as indicated. **f**, Quantification of surface markers in intestinal CD45^+^Dump^−^ IELs. For CD103, γδ^TCR^ *n* = 3 versus γδ^X^ *n* = 3; CD27, γδ^TCR^ *n* = 3 versus γδ^X^ *n* = 3; CD44, γδ^TCR^ *n* = 3 versus γδ^X^ *n* = 2; TIGIT, γδ^TCR^ *n* = 3 versus γδ^X^ *n* = 3; CD122, γδ^TCR^ *n* = 3 versus γδ^X^ *n* = 3. **g**,**h**, Genes that were significantly downregulated (blue) or upregulated (red) in RNA-seq analysis of small intestinal IELs from γδ^X^ (*n* = 4) compared to γδ^TCR^ (*n* = 3) mice 2 weeks post-tamoxifen. Heat maps show variance-stabilized transformation (VST)-normalized values for all (**g**) and a select subset of (**h**) DEGs. Each dot represents an individual mouse except for left bar charts in **a**,**b** (individual villus). Bars indicate mean ± s.d. *P* values determined with unpaired two-tailed *t*-test except in **g**,**h** (Methods). Source data

**Extended Data Fig. 4:**
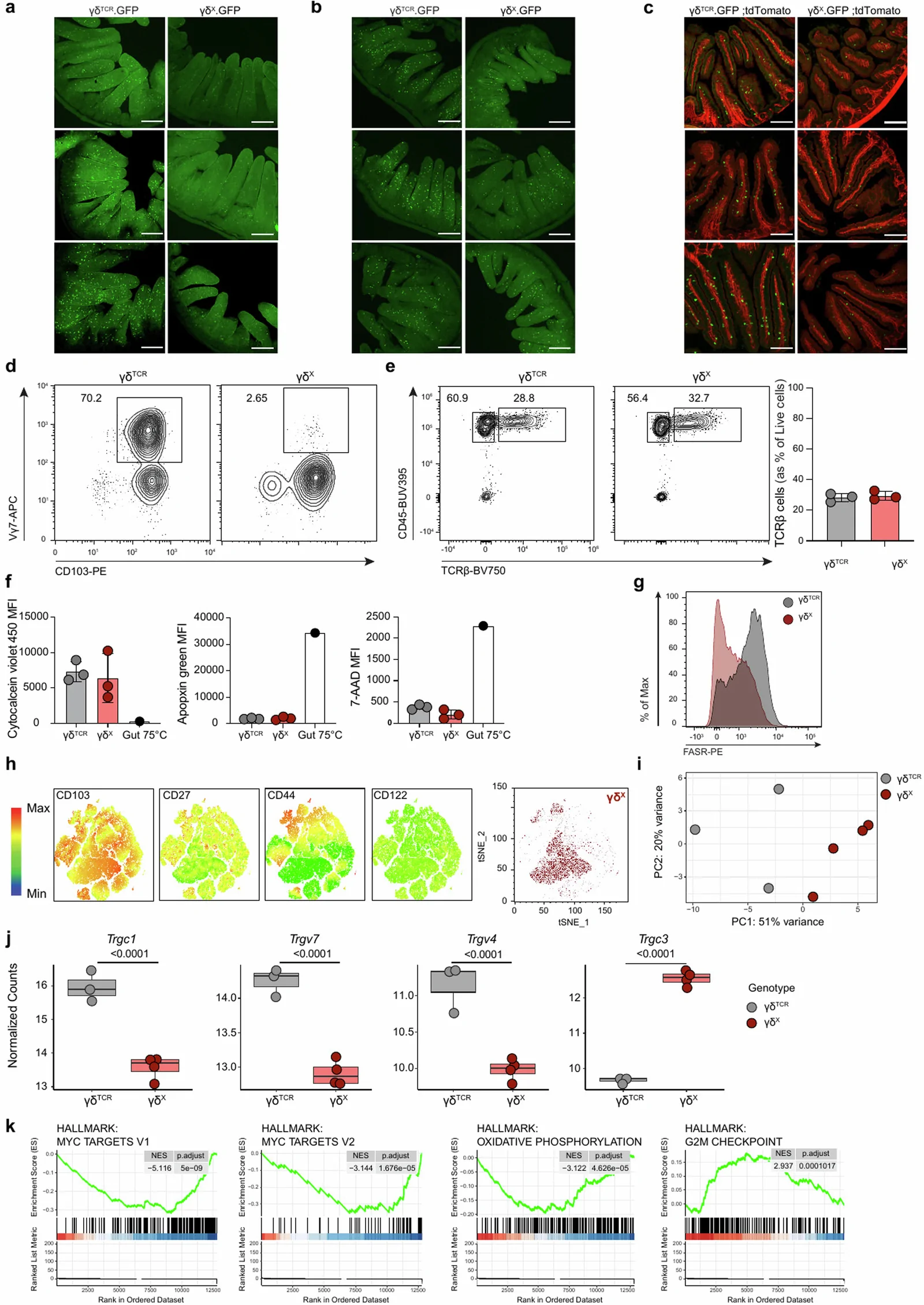
Phenotypic impact of TCRγδ deletion on small intestinal IEL. **a**,**b**, Representative confocal micrographs showing GFP^+^ lymphocytes distribution in 80 µm sections of small intestine from *Rosa26.mTmG*-γδ^TCR^ (*n* = 5) and *Rosa26.mTmG*-γδ^X^ (*n* = 5) mice 2- (**a**) and 4- (**b**) weeks post-tamoxifen. Data are representative of three independent experiments. **c**, Representative confocal micrographs showing GFP^+^ lymphocyte distribution with tdTomato as a counterstain in 20 µm sections of small intestine from *Rosa26.mTmG*-γδ^TCR^ (*n* = 5) and *Rosa26.mTmG-*γδ^X^ (*n* = 5) mice 2 weeks post-tamoxifen. **d**,**e**, Representative flow cytometry analysis of the proportion of Vγ7^+^ cells (**d**), and proportion of TCRβ^+^ cells (**e**, contour plots) and their quantification (**e**, bar charts) in intestinal CD45^+^Dump^neg^ IEL from *Rosa26*^*CreERT2*−^γδ^TCR^ (*n* = 3) and *Rosa26*^*CreERT2*−^γδ^X^ (*n* = 3) mice 2 weeks post-tamoxifen. f, Flow cytometry analysis of small intestine IEL viability from *Rosa26*^*CreERT2*^*-*γδTCR (*n* = 3) and *Rosa26*^*CreERT2*^*-*γδ^X^ (*n* = 3) mice 2 weeks post-tamoxifen using Cytocalcein (left), Apopxin (centre) and 7-AAD (right) that label viable, early-apopotic, and late-apoptotic/dead cells respectively. Cells incubated for 10 min at 75 ˚C were included as a positive control. g, Flow cytometry analysis of FasR expression by intestinal IEL from γδ^TCR^ and γδ^X^ mice 2 weeks post-tamoxifen. **h**, Representative plots for the expression of the indicated makers analysed by spectral flow cytometry in Fig. 3f (right, t-SNE plot repeated from Fig. 3e to facilitate the identification of each population). **i**-**k**, RNA-seq dataset on intestinal IEL from γδ^TCR^ (*n* = 3) and γδ^X^ (*n* = 4) mice (Fig. 3g,h) was analysed by: PCA (**i**); expression levels of VST-normalised counts for the indicated mRNAs relating to TCR gene usage (**j**, boxes indicate median, interquartile range and minimum/maximum); running enrichment score for the top 4 significantly enriched gene sets from MSigDB Hallmark gene sets based on NES values (**k**). White scale bars (**a**-**c**), 100 µm. All adjusted *p*-values (padj) were determined with DESeq2 package using the Benjamini–Hochberg procedure (for details, see Methods). Each dot in **e**, **f**, **i** and **j** represents an individual mouse; bars in **e** and **f** indicates mean ± SD; statistical significance in **e** and **f** was ruled out with unpaired two-tailed *t*-test. Source data

Having established core TCR dependencies of different types of skin γδ T cell, we investigated the effects of acute TCR deletion in the gut. Micrographs of 80-μm sections of γδ^X^ *mTmG* small intestines at 2 and 4 weeks post-tamoxifen treatment revealed reductions in average numbers of GFP^+^ cells per villus of around 50% in γδ^X^ *mTmG* versus γδ^TCR^ *mTmG* mice (Fig. 3a,b and Extended Data Fig. 4a,b). Moreover, thinner sections, which inevitably displayed fewer GFP^+^ cells, clearly showed that most were aligned with the epithelium (Extended Data Fig. 4c). Reductions of IEL numbers in γδ^X^ *mTmG* mice were similarly evidenced by flow cytometry, which also showed the substantial loss of Vγ7^+^ and Vγ4^+^ cells, whereas while Vγ1-Cγ4^+^ cells (where Cre is active but the TCR is not targeted) were largely unaffected (Fig. 3c).

Selective losses of intestinal TCR-ablated GFP^+^ cells might reflect their displacement by GFP^−^Vγ7-Cγ1^+^ cells developing from progenitors in which *Tcrd-creERT2* was not expressed. Thus, we utilized *Rosa26-creERT2* to ensure *Trgc1* deletion in progenitors and now observed that more than 90% of Dump^−^CD103^+^ cells of γδ^X^ mice were Vγ7^−^ (Extended Data Fig. 4d). Crucially, the yield of intestinal Dump^−^CD103^+^ cells from *Rosa26-creERT2*-γδ^X^ mice was invariably reduced by about 30–50% compared with that from *Rosa26-creERT2*-γδ^TCR^ mice (Fig. 3d), and, just as there was no compensatory expansion or influx of Vγ1-Cγ4 cells (Fig. 3c), there was also no compensatory expansion of TCRαβ^+^ IELs in the timeframe that we examined (Extended Data Fig. 4e). Thus, unlike in the skin, the maintenance of prototypic gut γδ IELs requires TCRγδ, and the cells’ loss cannot be ascribed to displacement by other cells.

Nonetheless, primary γδ^X^ IELs showed no significant differences in apoptosis or necrosis relative to γδ^TCR^ IELs (Extended Data Fig. 4f). We therefore assessed γδ^X^ IELs for FAS (also known as CD95) expression since *Fas*-deficient mice cannot stably retain Vγ7^+^ IELs^38^. Indeed, we observed significant surface downregulation of FAS relative to γδ^TCR^ IELs (Extended Data Fig. 4g).

Further phenotypic differences between small intestinal γδ^X^ and γδ^TCR^ IELs were evidenced by the cells’ clear segregation in spectral flow cytometry, validated a posteriori by CD3, TCRγδ, Vγ7 and Vγ4 expression profiles (Fig. 3e). As in skin, γδ^X^ IELs either retained or upregulated CD103 expression, whereas there was aggregate downregulation of TIGIT, CD27 and CD44, and fewer γδ^X^ IELs expressed CD122 (IL2Rβ) (Fig. 3f and Extended Data Fig. 4h), as was observed for Vγ7 IELs of mice acutely depleted of *Btnl1*^19^.

Next, we compared CD45^hi^Dump^−^ cells from γδ^X^ and γδ^TCR^ mice using RNA-seq (Supplementary Table 1). Again, PC1, which accounted for most of the variance, showed clear transcriptome segregation according to genotype, and as expected *Trgc1, Trgv7* and *Trgv4* were downregulated and *Trgc3* was upregulated (Extended Data Fig. 4i,j). Similarly to γδ^X^ DETCs, γδ^X^ IELs (which are also BTNL-selected) displayed relatively few DEGs: 77 upregulated and 63 downregulated genes, versus 12,683 that were unchanged (Fig. 3g). Furthermore, as for γδ^X^ DETCs and dermal cells, γδ^X^ IELs downregulated *Tigit*, *Xcl1* and ‘Myc targets’, whereas *Jun* and *Junb* were most often upregulated (Fig. 3h and Extended Data Fig. 4k).

Similarly, γδ^X^ intestinal IELs also showed TCRγδ dependencies of innate immune receptor RNAs. Downregulated genes included: *Klrb1a* (encoding Ly55a), which was also downregulated in epidermal and dermal γδ^X^ cells; *Klre1* (encoding NKG2I) which trended downwards in epidermal and dermal γδ^X^ cells; and *Klra6* (encoding Ly49F), *Klra3* (encoding Ly49C) and *Klrh1* (Fig. 3h). *Klra5*, which was significantly downregulated in epidermal and dermal γδ^X^ cells, also trended downwards in γδ^X^ IELs. In GSEA, ‘oxidative phosphorylation’ was downregulated in gut IELs (Extended Data Fig. 4k) and in dermal γδ^X^ cells, and mRNAs that were significantly downregulated in at least three of four gut γδ^X^ samples showed strong concordance with those that were downregulated in all three dermal γδ^X^ samples (Supplementary Table 3). Thus, whereas epidermal, dermal and small intestinal intraepithelial γδ T cells are highly distinct from one another, each was overtly affected by acute TCR ablation with several shared phenotypic changes.

**Extended Data Fig. 5:**
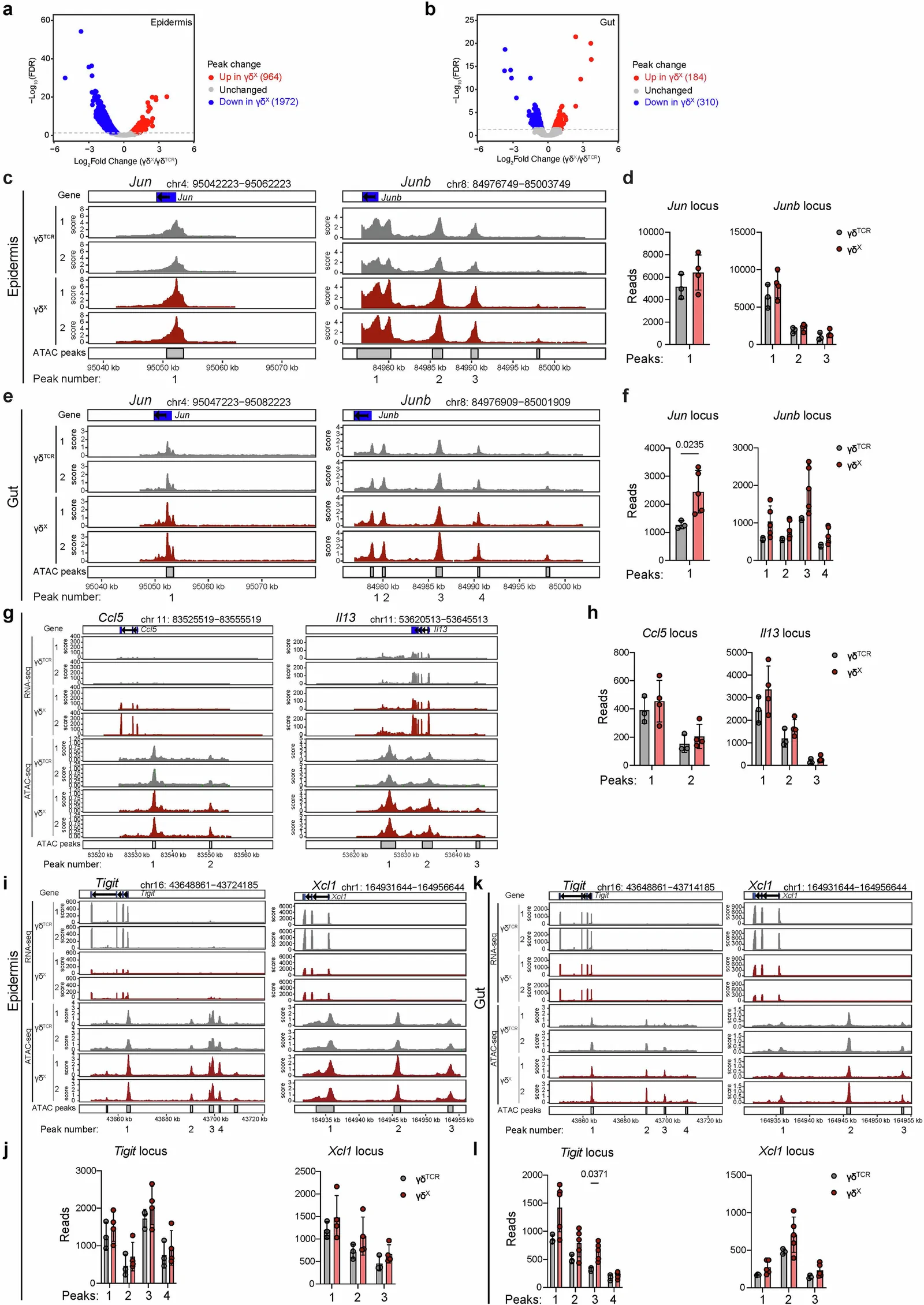
Impact of TCRγδ deletion on chromatin. **a**,**b**, Chromatin regions with significantly increased (red dots) or decreased (blue dots) accessibility in epidermal (**a**) and small intestinal (**b**) γδ^X^ versus γδ^TCR^ CD45^high^Dump^neg^ cells (FDR < 0.05), following ATAC-seq analysis at 1-week post-tamoxifen. **c-f**, Representative chromatin accesibility tracks and read count quantification for selected peaks at the *Jun* and *Junb* loci of epidermal lymphocytes (**c** and **d**; γδ^TCR^, *n* = 3; γδ^X^, *n* = 4) and small intestinal IEL (**e** and **f**; γδ^TCR^, *n* = 3; γδ^X^, *n* = 5) following ATAC-seq analysis. **g**,**h**, Representative paired RNA-seq (upper four tracks) and ATAC-seq tracks (lower four tracks) at *Ccl5* and *Il13* loci for epidermal CD45^high^Dump^neg^ cells from two γδ^TCR^ mice (grey peaks) and γδ^X^ mice (red peaks) following ATAC-seq analysis; with associated quantification (**h**) of ATAC-seq read counts for the indicated numbered peaks; γδ^TCR^, *n* = 3; γδ^X^, *n* = 4. **i**, **j**, As in **g**, **h**, but at the *Tigit* and *Xcl1* loci, as indicated; γδ^TCR^, *n* = 3; γδ^X^, *n* = 4. **k**,**l**, As in **i**, **j** but for small intestinal CD45^high^Dump^neg^ IEL; γδ^TCR^, *n* = 3; γδ^X^, *n* = 5. Each dot in **d**, **f**, **h**, **j** and **l** represents an individual mouse; bars indicate mean ± SD; *p*-values with unpaired two-tailed *t*-test. Source data

Because a cell’s steady-state phenotype and its responsiveness are regulated epigenetically, we used the assay for transposase-accessible chromatin using sequencing (ATAC-seq) to investigate whether TCRγδ regulated chromatin accessibility in DETCs and small intestinal IELs (note: for dermal cells, this analysis was precluded by insufficient cell recovery and viability). Although chromatin accessibility (peak heights) was largely comparable in γδ^TCR^ and γδ^X^ cells (31,373 sites in epidermis and 26,136 in gut), a small fraction showed accessibility that segregated with genotype (Extended Data Fig. 5a,b). This fraction was larger in epidermal versus gut γδ^X^ cells, despite their comparable numbers of TCR-dependent DEGs.

Consistent with increased gene expression, the *Jun* and *Junb* loci consistently trended towards greater accessibility in γδ^X^ cells, in one case reaching significance (Extended Data Fig. 5c–f). Increased *Ccl5* and *Il13* expression in epidermal γδ^X^ cells was similarly reflected in heightened chromatin accessibility (Extended Data Fig. 5g,h). Nonetheless, epidermal and intestinal γδ^X^ cells displayed atypical patterns for *Tigit* and *Xcl1* in that significantly reduced expression was associated with increased chromatin accessibility, again reaching significance in one case (Extended Data Fig. 5i–l). One or more factors downstream of steady-state TCR signalling may occlude labile repressors from these loci, which are therefore bound more readily upon TCR loss.

## TCR dependencies of human Vδ1^+^ cells

**Extended Data Fig. 6:**
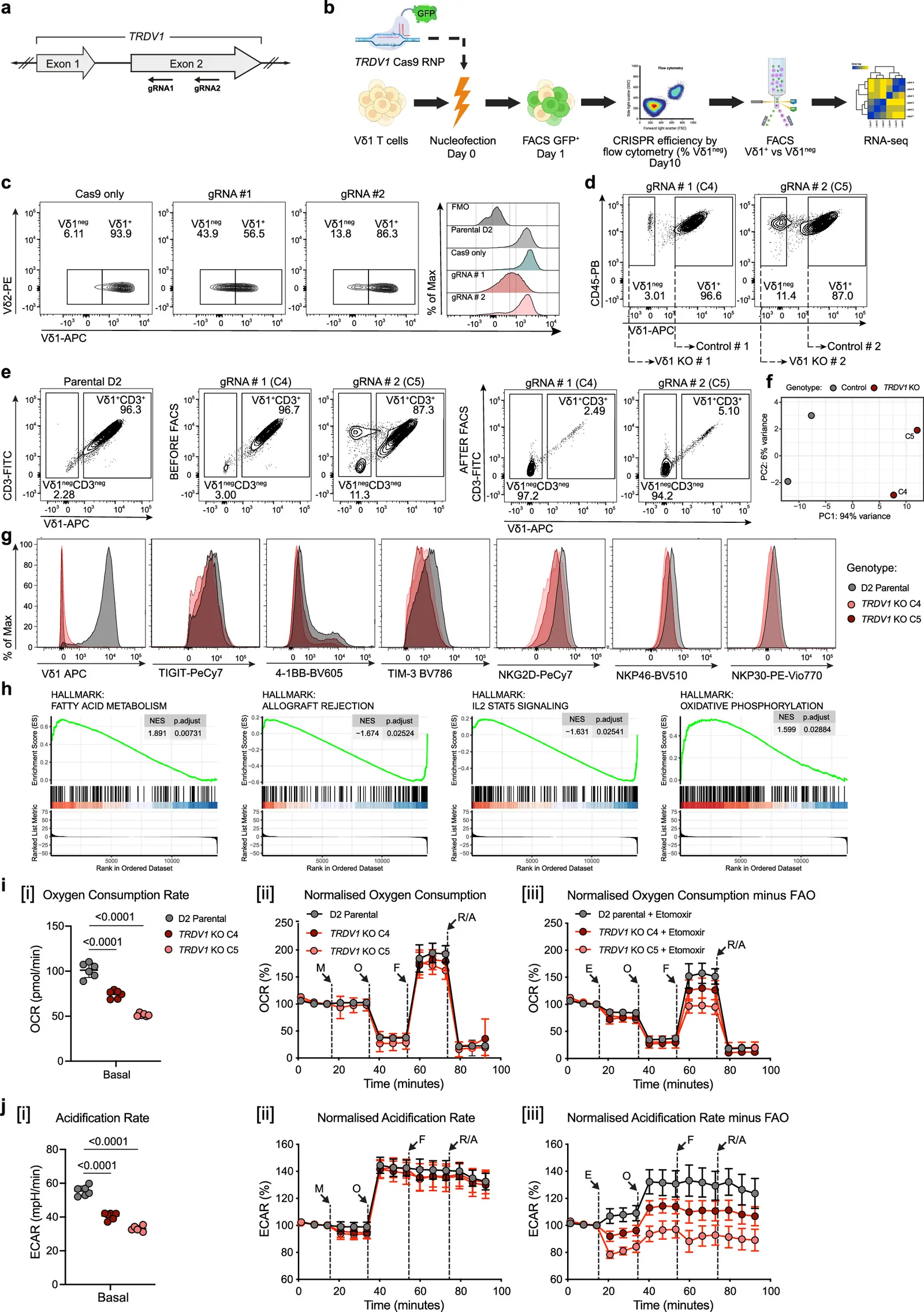
*TRDV1* deletion in human Vδ1^+^ T cells. **a**,**b**, CRISPR strategy and workflow for the generation of human *TRDV1* KO cell lines C4 and C5 using two guide RNAs (gRNA 1 and 2 respectively) targeting different regions of *TRDV1* exon 2 (**a**). Vδ1^+^ cells (parental line D2) were nucleofected with Cas9/gRNA complexes and GFP^+^ cells sorted one day later. KO efficiency was assessed by flow cytometry 10-days post-nucleofection, and Vδ1^+^ and Vδ1^neg^ subsets sorted for subsequent RNA-seq analysis. Diagram was created in BioRender; Veland, N. https://BioRender.com/zsheji4 (2026) (**b**). **c**, Representative flow cytometry analysis of Vδ1 expression following the indicated treatments, highlighting increased percentage of Vδ1^neg^ cells in gRNA-nucleofected samples. **d**, Gating strategy used for sorting Vδ1^+^ (control) and Vδ1^neg^ (*TRDV1* KO) cells for subsequent RNA-seq analysis. **e**, Flow cytometry analysis of Vδ1 and CD3 expression in the parental (D2), compared to *TRDV1* KO (C4 and C5) lines before and after sorting. **f**, RNA-seq PCA showing control Vδ1^+^ cells (*n* = 2) and *TRDV1* KO cells (*n* = 2). **g**, Representative flow cytometry histograms for the expression of the indicated proteins quantified in Fig. 4b,c. **h**, NES-based running enrichment scores for the top 4 significant gene sets (Human MSigDB Hallmark) in *TRDV1* KO versus Control cells (for significance, see Methods). **i**, **j**, Real-time mitochondrial metabolic profiling of *TRDV1* KO (C4, C5) compared to parental (D2) lines, showing (**i**) Basal oxygen consumption rate (OCR), *n* = 6 per cell line [i], and normalised OCR percentage after sequential injections (vertical dashed lines and arrows) into medium (M) of oligomycin (O), FCCP (F), and rotenone/antimycin A (R/A) without [ii] or with [iii] prior inhibition of fatty acid oxidation (FAO) with etomoxir (E); and (**j**) Basal extracellular acidification rate (ECAR), *n* = 6 per cell line [i], and normalised ECAR percentage without [ii] or with [iii] prior inhibition of FAO, using the same method as in **i**. Each dot in [ii] and [iii] of **i** and **j** indicates the mean ± SD of technical replicate wells ([ii] D2 *n* = 7, C4 and C5 *n* = 9; [iii] D2 *n* = 6, C4 and C5 *n* = 9); *p*-values determined with one-way ANOVA using Dunnet’s test for multiple comparison correction. Source data

Next, we explored whether human γδ T cells expressing Vδ1, the main TCRγδ chain utilized by tissue-intrinsic human γδ T cells^1^, might similarly show acute TCR dependencies. Because we clearly could not target Vδ1 in cultures requiring sustained TCR stimulation^39^, we examined human T-lymphotropic virus-1 (HTLV1)-immortalized Vδ1^+^ cell lines for which this is not the case^40^. Ten days following CRISPR-mediated *TRDV1* gene targeting of parental cells (D2), we flow-sorted two sets of cultures, achieving 94–97% enrichment of Vδ1^−^ cells (C4 and C5), and immediately compared their transcriptomes to those of residual TCRVδ1^+^ cells from the same cultures (Extended Data Fig. 6a–e).

**Fig. 4:**
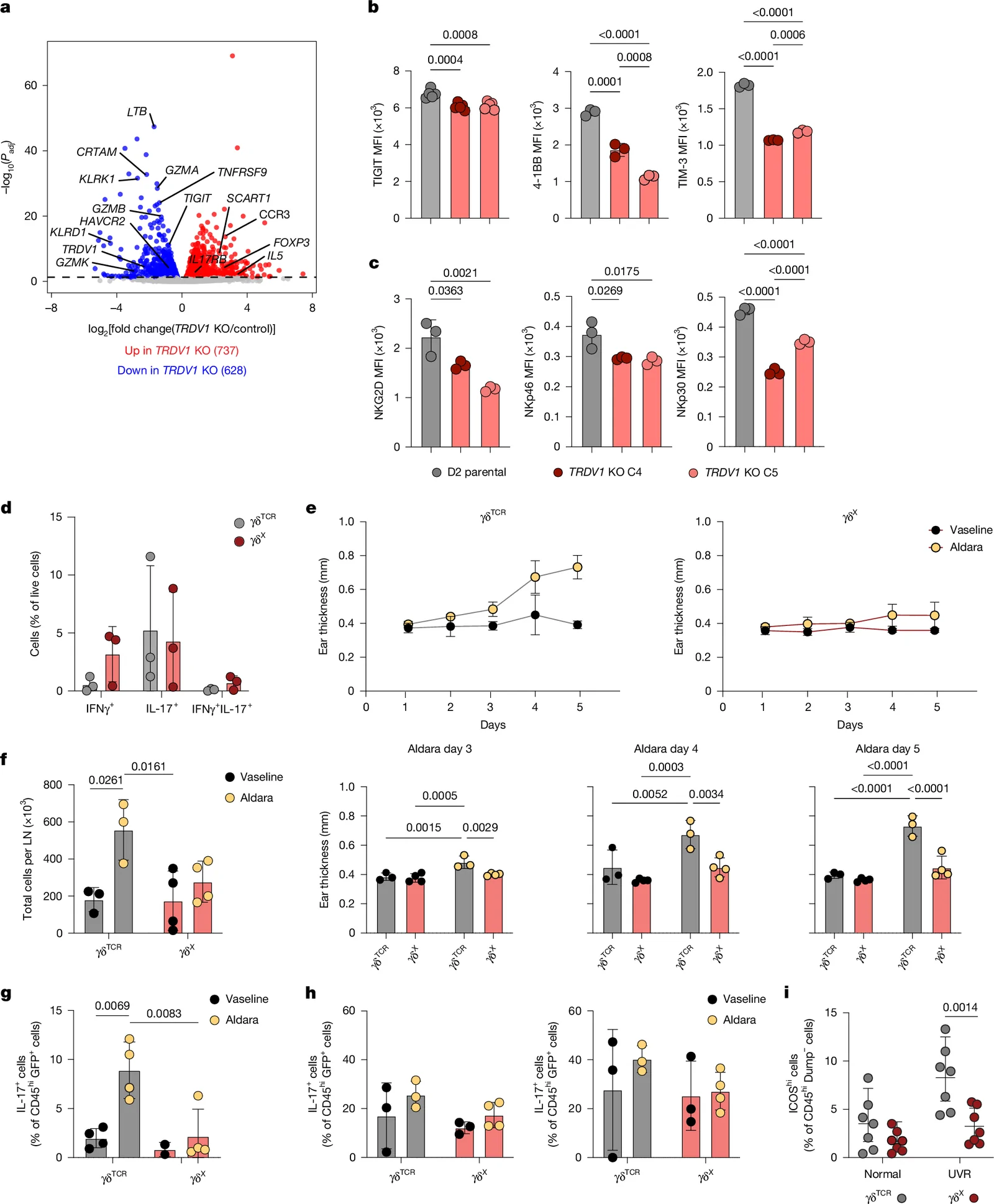
TCRγδ deletion affects human γδ T cells and impairs innate responses. **a**, DEGs that were upregulated (red) or downregulated (blue) in RNA-seq analysis of Vδ1^−^ (*TRDV1*-knockout (KO)) versus Vδ1^+^ cells (control) sorted 10 days post-nucleofection with two different Cas9–guide RNA (gRNA) complexes (Methods). **b**,**c**, Quantitative flow cytometry analysis of cell surface expression of indicated proteins by two *TRDV1*-KO lines (C4 and C5) and a parental Vδ1^+^ counterpart (D2). TIGIT, *n* = 5; 4-1BB, TIM3, NKG2D, NKp46 and NKp30, *n* = 3. Each dot represents an independent replicate. Bars indicate mean ± s.d. *P* values determined by one-way ANOVA with Tukey’s multiple comparisons test. **d**, Quantitative intracellular flow cytometry of skin CD45^hi^Dump^−^ cells producing IL-17, IFNγ or both from γδ^TCR^ (*n* = 3) or γδ^X^ (*n* = 3) mice 2 weeks post-tamoxifen, following collection and 21 h of in vitro stimulation with IL-12, IL-18, IL-1β and IL-23. **e**, Measurement of ear thickness at indicated days following application of Aldara to one ear and Vaseline to the contralateral ear of γδ^TCR^ (*n* = 3 average) and γδ^X^ (*n* = 4 average) mice, 1 week post-tamoxifen. Bar charts show ear thickness for individual γδ^TCR^ (*n* = 3) and γδ^X^ (*n* = 4) mice on indicated days. **f**–**h**, Flow cytometry analysis of ear-draining lymph node (LN) cells from *Rosa26-mTmG*-γδ^TCR^ (*n* = 3) and *Rosa26-mTmG*-γδ^X^ (*n* = 4) mice 1 week post-tamoxifen, and following 5 days of Aldara or Vaseline application, for cellularity (**f**; γδ^TCR^, *n* = 3 versus γδ^X^ *n* = 4) and frequency of IL-17-production directly ex vivo (**g**; γδ^TCR^ *n* = 4 versus γδ^X^ *n* = 4), or following in vitro stimulation with IL-1β plus IL-23 (for 21 h) or PMA plus ionomycin (for 5 h) (**h**; γδ^TCR^ *n* = 3 versus γδ^X^ *n* = 4). **i**, Flow cytometry analysis of ICOS expression in epidermal cells from γδ^TCR^ (*n* = 7) and γδ^X^ (*n* = 7) mice 1 week post-tamoxifen under normal and post-ultraviolet radiation conditions. Dots in bar charts in **d**–**h** and dots in **i** represent individual mice. Bars indicate mean ± s.d. *P* values determined with two-way ANOVA. Source data

Whereas HTLV1 immortalization might have been predicted to override any steady-state TCRγδ dependencies, this was not the case with transcriptomes that segregated according to TCR genotype in PC1, accounting for more than 90% of the variance (Extended Data Fig. 6f). No statistical assessment of the PCA was made because it was a 2 × 2 comparison, but we could nonetheless identify around 1,400 DEGs in TCR^−^ cells, that were divided almost equally into upregulated and downregulated genes (Fig. 4a), several of which were selected for independent validation by flow cytometry.

Parallels with mouse TCRγδ dependencies were conspicuous, including downregulation of *XCL1* (for which there was a trend) and of several other mRNAs and surface proteins—for example, *TIGIT*, *TNFSFR9* (encoding 4-1BB), *HAVCR2* (encoding TIM3) and KLR and NKR genes (Fig. 4b and Extended Data Fig. 6g), including *KLRB1* (encoding CD161 (also known as Ly-55)), which was downregulated in all mouse subsets examined and which trended in that direction despite very low expression by parental Vδ1^+^ cells. Also downregulated were *KLRD1* (encoding CD94) and *KLRK1* and its protein product NKG2D, together with NKp46 and NKp30, all of which can promote innate cytolytic responses (Fig. 4c and Extended Data Fig. 6g). Other downregulated mRNAs encoding cytolytic regulators were *GZMA*, *GZMB*, *GZMK* and *CRTAM*, whereas mRNAs associated with T_H_2 cell biology, including *IL5*, *CCR3* and *IL17RB* were upregulated (Fig. 4a), with *IL9* and *IL13* trending towards upregulation (Supplementary Table 1), thus evoking *Il13* upregulation in mouse epidermal and dermal γδ^X^ cells.

In sum, several TCR-regulated genes and proteins in mouse tissue-intrinsic γδ T cells were also TCR-dependent in virus-immortalized human Vδ1^+^ T cells. Additionally, gene sets regulating metabolism were affected by TCR ablation in human Vδ1^+^ T cells, as in mouse dermal and intestinal γδ T cells (Extended Data Fig. 6h and Supplementary Table 3). Indeed, the two TCR^−^ lines (C4 and C5) showed reduced metabolic activity compared with parental Vδ1^+^ cells (D2), reflected in lower basal oxygen consumption rates (OCR) and extracellular acidification rates (ECAR) (Extended Data Fig. 6i(i),j(i)). Although the TCR^−^ lines had similar spare respiratory capacity (Extended Data Fig. 6i(ii),j(ii)), this reserve was greatly diminished in TCR^−^ cells when fatty acid oxidation (FAO) was inhibited by etomoxir, with ECAR also being more sensitive to etomoxir treatment (Extended Data Fig. 6i(iii),j(iii)). Thus, TCR ablation rapidly shifted human Vδ1 cells to greater dependence on FAO, as predicted by the transcriptomics analyses.

## TCR ablation impairs innate responses

**Extended Data Fig. 7:**
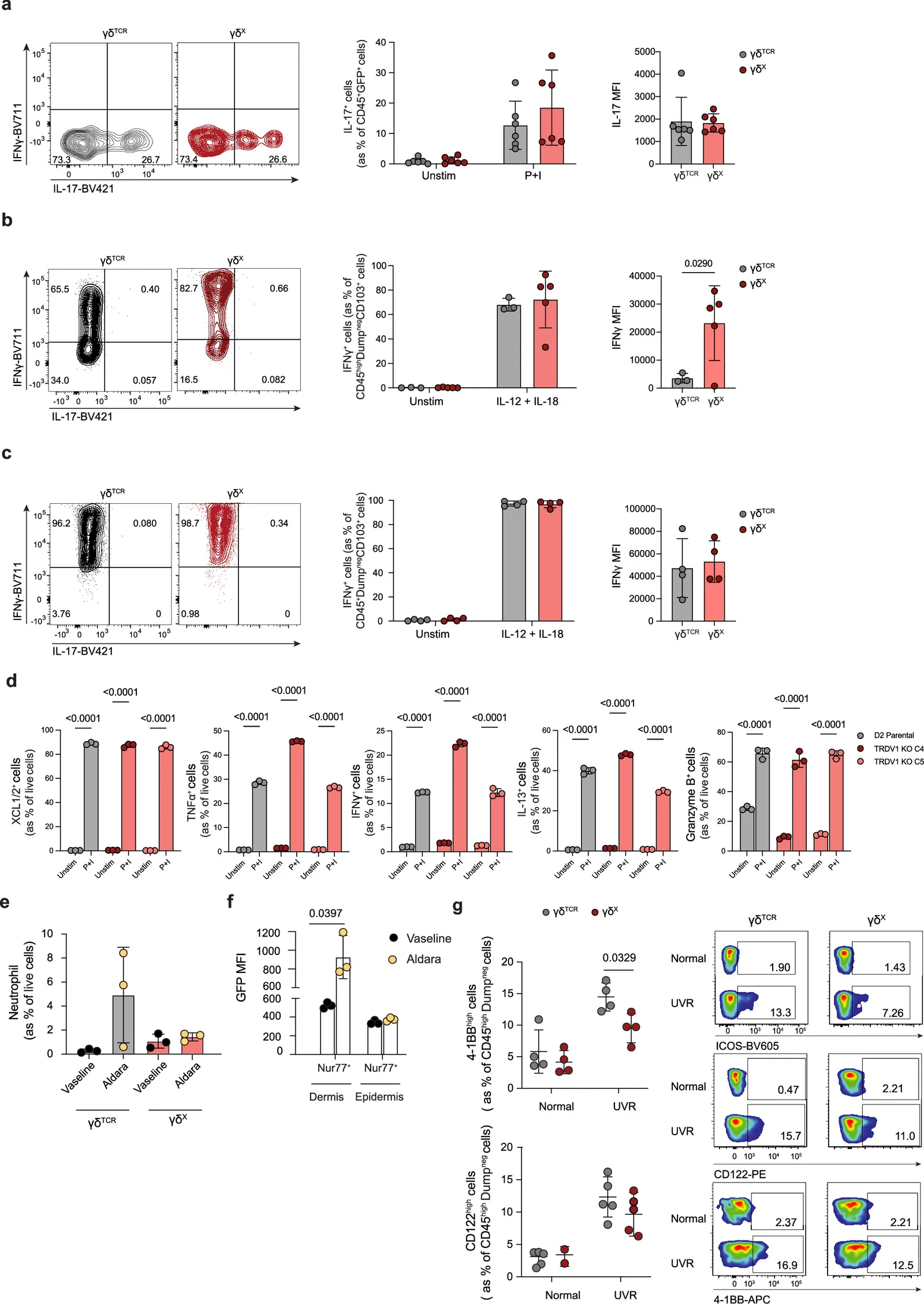
Functional impacts of γδ TCR deletion. **a**-**c**, Flow cytometry analysis of IFNγ and IL-17 production, showing representative stainings (contour plots) and quantification (bar-charts) for dermal GFP^+^ cells from *Rosa26.mTmG*-γδ^TCR^ (*n* = 6) and *Rosa26.mTmG*-γδ^X^ (*n* = 6) mice 1-week post-tamoxifen, following 5-hours in vitro culture without (unstim) or with stimulation with PMA + Ionomycin (P + I) (**a**); for epidermal lymphocytes from γδ^TCR^ (*n* = 3) and γδ^X^ (*n* = 5) mice, isolated and tamoxifen-treated in vitro for 1-week prior to 21-hours culture without (unstim) or with stimulation with IL-12 + IL-18 (**b**); and for intestinal γδ IEL from *Rosa26*^*CreERT2*^-γδ^TCR^ (*n* = 4) and Rosa26^*CreERT2*^ γδ^X^ (*n* = 4) mice 1-week post-tamoxifen, following 21-hours in vitro culture without (unstim) or with stimulation with IL-12 + IL-18 (**c**). **d**, Flow cytometry analysis of the indicated proteins expression in *TRDV1* KO (C4 and C5) and parental Vδ1^+^ (D2) cells following 3 h culture without (unstim) or with P + I stimulation (*n* = 3 per cell line). **e**, Flow cytometry analysis of neutrophil (MHC^neg^ CD11b^+^) frequencies following a 5-days application of Aldara (yellow) to one ear and Vaseline (black) to the contralateral ear of γδ^TCR^ (*n* = 3) and γδ^X^ (*n* = 3) mice 1-week post-tamoxifen. **f**, Flow cytometry analysis of GFP expression levels in dermal and epidermal γδ cells following a 5-days application of Aldara (yellow) to one ear and Vaseline (black) to the contralateral ear of *Nur77.GFP* mice (*n* = 3). **g**, Flow cytometry analysis showing representative stainings (contour plots) and quantification (charts) of the indicated proteins expression by CD45^+^Dump^neg^ epidermal lymphocytes from γδ^TCR^ and γδ^X^ mice 1-week post-tamoxifen under normal and UVR conditions. Each dot represents an individual mouse (**a**, **b**, **c**, **e**, **f** and **g**) or human cell line stimulation replicate (**d**); bars represent mean ± SD; *p*-values determined with unpaired two-tailed *t*-test. Source data

The next question was whether acute TCRγδ loss affected the cells’ competence to respond to stimuli. First, comparable effector responsiveness of dermal γδ^X^ and γδ^TCR^ cells was evidenced by IL-17 production in response to PMA plus ionomycin (P + I), which bypasses the TCR to potently activate downstream signalling pathways (Extended Data Fig. 7a). Likewise, total skin cells from γδ^X^ and γδ^TCR^ mice responding innately to cytokines IL-1β and IL-23 showed variable but comparable IL-17 production (Fig. 4d).

Although DETCs are skewed away from IL-17^21^, their innate response to cytokines IL-12 plus IL-18 evokes little IFNγ, in which context, γδ^X^ DETCs trended towards increased IFNγ production (Fig. 4d). Moreover, when IFNγ production was increased by adapting DETCs to short-term culture, *Trgc1* gene ablation in vitro further increased responsiveness (Extended Data Fig. 7b). Similarly, gut γδ^X^ IELs were at least as robust as γδ^TCR^ IELs in producing IFNγ (Extended Data Fig. 7c), and the human cell line D2 and its TCR-deficient derivatives C4 and C5 showed comparable effector responses to PMA plus ionomycin (Extended Data Fig. 7d). In sum, acute TCR deletion impaired neither the innate responsiveness nor the functional skewing of mouse and human γδ T cells in vitro. We therefore examined innate γδ T cell responses in vivo.

Epicutaneous ear exposure to Aldara cream (also known as imiquimod) stimulates local myelomonocytic cells to produce IL-1β and IL-23, rapidly provoking dermal γδ T cells to release IL-17 that recruits neutrophils and elicits psoriaform skin-thickening over 3–5 days^41^. Notably, this response was almost fully ablated in ears of γδ^X^ mice (Fig. 4e and Extended Data Fig. 7e). Indeed, whereas draining lymph nodes ordinarily swell and become replete with IL-17-producing γδ T cells, neither occurred in Aldara-treated γδ^X^ mice (Fig. 4f,g). Nonetheless, the potential of draining lymph node γδ^X^ cells to produce IL-17 was evident following their isolation and exposure to cytokines in vitro (Fig. 4h). Thus, the prototypic innate γδ T cell response to Aldara in vivo required TCRγδ in real time. Indeed, Aldara treatment ordinarily induced TCRγδ activation, as evidenced by Nur77-GFP mice, in which increased GFP expression reflected TCR signalling^42^ (Extended Data Fig. 7f).

Another prototypic innate γδ T cell response is the rapid upregulation by DETCs of the activation markers, 4-1BB, ICOS and CD122 following skin exposure to ultraviolet irradiation^24^. By contrast, the 4-1BB and ICOS responses were significantly blunted in γδ^X^ mice that were irradiated after 7 days of tamoxifen treatment, with CD122 induction also trending downward (Fig. 4i and Extended Data Fig. 7g).

## TCR dependence of cancer surveillance

**Fig. 5:**
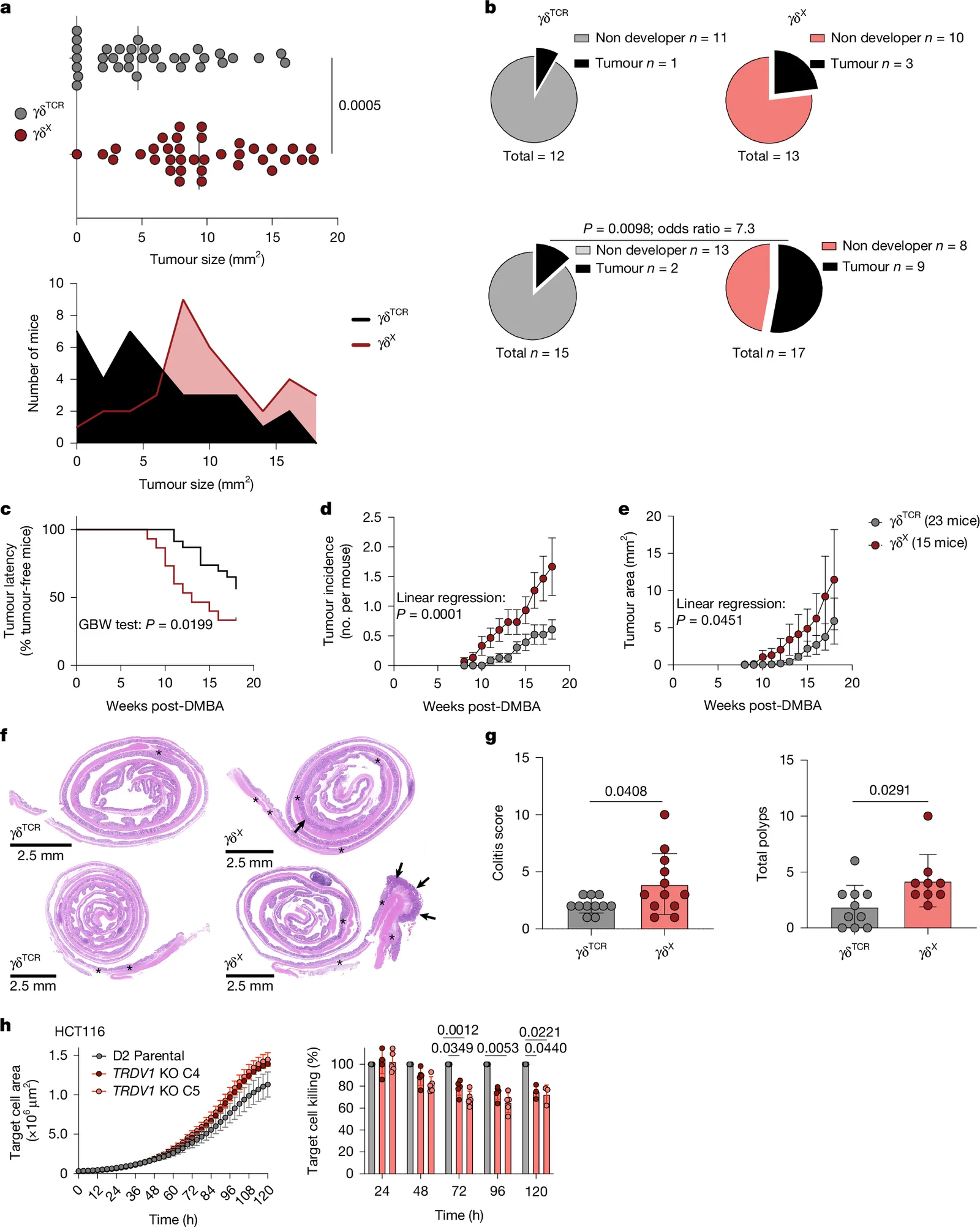
TCRγδ dependence of cancer immunosurveillance. **a**, Tumour development starting 5 days post-subcutaneous injection of B16-F10 melanoma cells into γδ^TCR^ (*n* = 35) and γδ^X^ (*n* = 36) mice 1 week post-tamoxifen treatment, and continuing until day 10 after subcutaneous cell injection, showing tumour sizes per mouse (top) and size distribution (bottom). Vertical lines represent the mean for each condition. **b**, Analysis of indicated numbers of tumour developers and non-developers 17 days after epicutaneous application of 2 × 10^5^ (top) or 4 × 10^5^ (bottom) B16-F10 cells to γδ^TCR^ and γδ^X^ mice 1 week post-tamoxifen. Odds ratio determined with Fisher’s exact test. **c**–**e**, Tumour development following DMBA plus TPA-induced carcinogenesis in γδ^TCR^ (*n* = 23) and γδ^X^ (*n* = 15) mice starting 1 week post-tamoxifen and continuing until day 42 post-DMBA, showing tumour latency (**c**), incidence (**d**) and area (**e**). GBW, Gehan–Breslow–Wilcoxon. **f**,**g**, Illustrative histopathology of inflammatory and carcinoma lesions 14 weeks post-AOM plus DSS treatment of γδ^TCR^ (*n* = 11) and γδ^X^ (*n* = 12) mice initiated 1 week post-tamoxifen (**f**) and associated colitis scores (**g**, left). γδ^TCR^ mice exhibit discrete foci of inflammatory infiltrates (asterisks). γδ^X^ mice exhibit several areas of adenocarcinoma and hyperplasia (arrows) and extensive and multiple foci of inflammatory infiltrates (asterisks). **g**, Right, Quantification of polyps based on visual (Extended Data Fig. 8g) and histopathological analyses (γδ^TCR^, *n* = 10; γδ^X^, *n* = 9). **h**, Representative growth curves over 5 days for mCherry-expressing HCT116 cancer cells co-cultured with the indicated T cell lines (left) and quantification of target cell loss at the indicated time points in co-cultures with *TRDV1*-KO C4 and C5 normalized to co-cultures with Vδ1^+^ D2 parental cells for visualization (right, *n* = 5). Each dot represents an individual mouse (**a**,**g**), mouse genotype (**d**,**e**), cell line average time point (**h**, left) or independent experiment (**h**, right. Data are mean ± s.e.m. (**c**–**e**) or mean ± s.d. (**g**,**h**). *P* values determined with unpaired two-tailed *t*-test (**a**,**g**), Gehan–Breslow–Wilcoxon test (**c**), linear regression analysis (**d**,**e**) or linear mixed-effects model with Dunnett’s test (**h**). Source data

Finally, we examined immunosurveillance responses to solid cancers. B16-F10 melanoma cells infused subcutaneously into C57BL/6 mice form tumours within 1–2 weeks, and although these are poorly controlled by endogenous T cells^43^, natural γδ T cell-mediated surveillance has been inferred from larger tumours developing more rapidly in mice deficient in γδ T cells^44^. This was conspicuously phenocopied by γδ^X^ versus γδ^TCR^ mice injected with tumour cells within 5 days of tamoxifen treatment (*n* = 71). Tumours formed in 80% of γδ^TCR^ mice compared with more than 97% of γδ^X^ mice, in which the modal tumour size was also larger (Fig. 5a).

**Extended Data Fig. 8:**
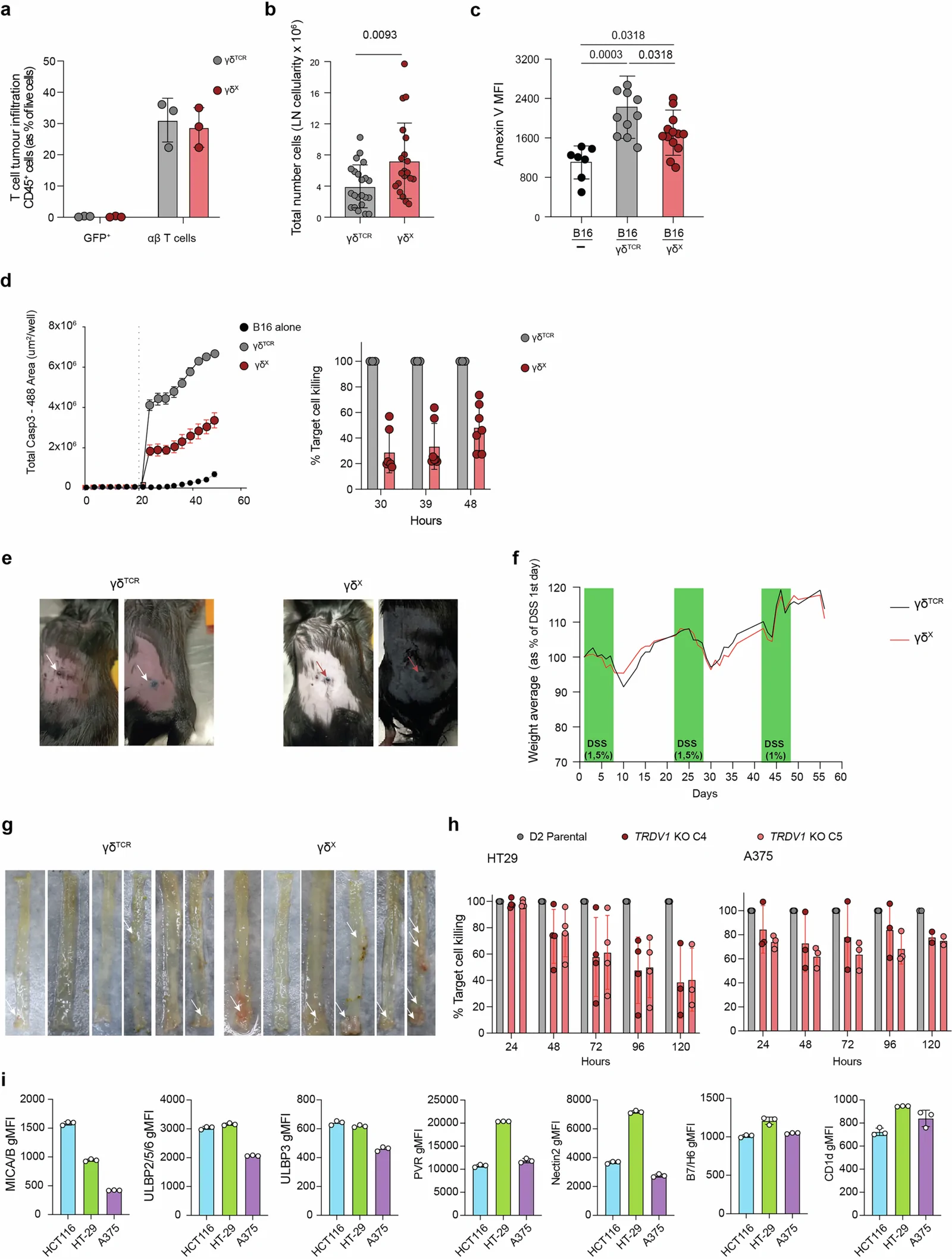
TCRγδ dependence of cancer immunosurveillance. **a**,**b**, Flow cytometry analysis of tumour-infiltrating GFP^+^ or TCRβ^+^ cells (**a**) and total LN cellularity (**b**) in γδ^TCR^ (*n* = 3) and γδ^X^ (*n* = 3) mice after subcutaneous injection of B16.F10 melanoma cells. **c**, Flow cytometry analysis of B16.F10 cells apoptosis determined by Annexin V staining following a 24-hours in vitro culture alone (−) or with CD45^high^Dump^neg^CD103^+^ cells (3:1 effector:target ratio) from γδ^TCR^ and γδ^X^ mice 2-weeks post-tamoxifen. **d**, IncuCyte analysis of NucLightRed-labelled B16.F10 cells killing following in vitro co-culture with epidermal short-term cultured lymphocytes from γδ^TCR^ (*n* = 3) and γδ^X^ (*n* = 3) mice 9 days post-4-Hydroxytamoxifen in vitro in the presence of Casp3-488, showing representative cell death curves (left, based on Casp3-488 area measurement) and percentage of target cell killing by γδ^X^ normalised to γδ^TCR^ cells for visualization (right). **e**, Representative images of epicutaneous tumours (arrows) after B16.F10 melanoma cells application in γδ^TCR^ and γδ^X^ mice used for the analysis in Fig. 5b. **f**, Average weight changes during AOM-DSS treatment of γδ^TCR^ and γδ^X^ mice from Fig. 5f,g. Green areas, DSS cycles. **g**, Representative images of colons following AOM-DSS treatment of γδ^TCR^ and γδ^X^ mice used for the analysis of polyp formation (white arrows) in Fig. 5g. **h**, IncuCyte analysis of mCherry-expressing HT-29 (left, *n* = 4) and A375 (right, *n* = 3) cancer cells killing by human *TRDV1* KO (C4 and C5) and parental Vδ1^+^ (D2) lines, showing percentages of target cell killing by *TRDV1* KO C4 and C5 normalized to D2 parental cells for visualization. i, Flow cytometry analysis of the indicated NKR ligands expression in mCherry-expressing HCT116 (*n* = 3), HT-29 (*n* = 3) and A375 (*n* = 3) cancer cell lines used in Fig. 5h and Extended Data Fig. 8h. Each dot represents an individual mouse (**a**, **b** and **c**), mouse or human (d and h respectively) target cell line time-point or replicate, or human cell line staining replicate (i); bars and curves represent mean ± SD; *p*-values determined with unpaired two-tailed *t*-test (a and b), one-way ANOVA (Holm-Šídák test) (**c**), or LMM with Dunnett’s test (**d** and **h**). Source data

Impaired tumour surveillance capacity reflected neither a lack of intratumoural GFP^+^ cells, since those were comparably rare in γδ^TCR^ and γδ^X^ mice, nor a failure to promote intratumoural αβ T cells, since their frequencies were also unaffected (Extended Data Fig. 8a). Indeed, γδ^X^ mice showed higher draining lymph node cellularity (Extended Data Fig. 8b), possibly reflecting persistent stimulation by larger tumours. However, partial defects in inducing B16-F10 cell apoptosis (measured by annexin V or caspase-3 staining) were shown by DETCs that were freshly isolated from tamoxifen-treated γδ^X^ mice or generated via tamoxifen treatment of short-term DETC cultures (Extended Data Fig. 8c,d).

Next, epidermal immunosurveillance was specifically assessed by epicutaneous application of B16-F10 cells to tape-stripped skin^45^. Over 30 days, only a fraction of mice developed tumours (Extended Data Fig. 8e), but this fraction was increased in γδ^X^ mice across three experiments using two different doses of cells. At the higher dose (4 × 10^5^ cells), the likelihood of γδ^X^ mice developing tumours was increased more than sevenfold compared with γδ^TCR^ cells (Fig. 5b).

γδ T cells also limit squamous cell carcinoma following epicutaneous application of a mutagen, 7,12-dimethylbenz[*a*]anthracene (DMBA), followed by an inflammatory tumour promoter, 12-*O*-tetradecanoylphorbol-13-acetate (TPA (also known as PMA))^46^. Although these tumours take months to develop in C57BL/6 mice, Vγ5Vδ1^+^ DETCs act early to limit cancer initiation^13^. In this context, tumour burden was significantly higher and tumour onset markedly accelerated in γδ^X^ mice (Fig. 5c–e).

Finally, an analogous system of intestinal carcinogenesis utilizes azoxymethane (AOM) as mutagen and repeated bouts of dextran sodium sulfate (DSS) as the pro-inflammatory promoter, and this too is limited by TCRγδ^+^ IELs^47^. In γδ^X^ and γδ^TCR^ mice, this modality elicited comparable cycles of acute weight loss and diarrhoea followed by recovery as illustrated for one of two experiments in Extended Data Fig. 8f. Over several months, the mice developed inflammatory lesions and polyps, as assessed macroscopically post-mortem (Extended Data Fig. 8g) and by blinded, independent histopathology, which showed that both inflammatory lesion severity and the numbers of polyps were significantly increased in γδ^X^ versus γδ^TCR^ mice (Fig. 5f,g).

Thus, all four cancer immunosurveillance systems tested were TCRγδ-dependent. Seeking parallels in human, we found that despite the demonstrated capacity of PMA plus ionomycin stimulation to evoke comparable effector responses from TCRγδ^−^ cells (C4 and C5) and parental Vδ1^+^ cells (D2), TCRγδ^−^ cells showed impaired capacity to limit the growth of each of three human cancer cell lines tested: colorectal cancers (HCT116 and HT29), and melanoma (A375), as assessed by IncuCyte, particularly after longer periods of T cell–target cell co-culture (Fig. 5h and Extended Data Fig. 8h). Of note, the collective expression by the tumour cell lines of NKR ligands (Extended Data Fig. 8i) effectively excluded the prospect that their absence created an atypical dependence on TCRγδ for optimal immunosurveillance.

## Discussion

Acute TCR ablation in subsets of mature, tissue-associated γδ T cells rapidly dysregulated their steady-state phenotypes and impaired immunosurveillance responses that are core components of γδ T cell biology, namely, cancer immunosurveillance, innate immune mobilization following chemical (Aldara) exposure, and regulation of barrier integrity. Thus, in all these settings, regulation by TCRγδ was overarching, consistent with TCRγδ being the probable driver of the high evolutionary conservation of γδ T cells.

Innate immune regulators—for example, NKRs—undoubtedly contribute substantially to γδ T cell-mediated surveillance, and may mediate residual competences of TCR-ablated cells, although those may also reflect incomplete penetrance of Cre-mediated deletion. Either way, TCRγδ ablation invariably affected KLR and NKR gene expression, with some targets shared across cell types (such as CD161, which is reported to be a key marker of cytokine-responsive, innate-like T cells^48^), and others being γδ subset-specific. These targets included Ly49-encoding genes whose variegated expression regulates innate ‘missing-self’ recognition of dysregulated cells^49^. Thus, ongoing TCRγδ activity may license mature γδ T cells for innate responsiveness, irrespective of any previous, TCR-dependent developmental programming.

Conspicuously, TCRγδ dependence was most overt in vivo, as exemplified by TCR-ablated cells producing IL-17 following cytokine stimulation in vitro but not after Aldara exposure in vivo. TCRγδ signalling might overcome unknown checkpoints that limit cell activation in vivo, but which are largely bypassed in vitro. This prospect raises the question of how TCRγδ is activated to mediate its real-time functions.

For DETCs and for mouse and human gut IELs, BTNL proteins mediate TCRγδ-dependent selection, and in mice some cognate T cell phenotypes and surveillance competences were impaired by inducible BTNL ablation or blockade^19,24^. However, those effects were not obviously synonymous with TCR ablation, for example, acute Btnl1 deletion did not affect intestinal IEL survival^19^, challenging attempts to ascribe real-time requirements for TCR signalling solely to BTNL engagement. Moreover, BTNL engagement clearly cannot explain the TCR dependence of BTNL-independent cells, including dermal γδ T cells and most human Vδ1^+^ cells.

TCRγδ can clearly mediate clonal responses to alterations in autologous ligands, for example, EPCR^12^, that are induced by cell transformation or infection. Moreover, Aldara stimulation reportedly expanded IL-17-producing TCRCγ1^+^ T cells that then responded better to re-challenge, a hallmark of adaptive immunity^50,51^. However, to explain their rapid actions, the relevant TCRs must probably be either polyreactive, as reported for mouse Vγ6-Vδ1^52^, or nonclonal, positing the existence of additional, unidentified BTNL-like elements. Alternatively, the TCR may function independently of ligand, for which precedent exists^53,54^, or the data may reflect tonic signalling. However, given that tonic signalling of αβ TCRs requires some components of clonotypic ligands, for example, MHC^55^, any structural basis of tonic TCRγδ signalling remains to be explained.

TCR ablation affected αβ T cells to varying degrees in a subset-specific manner^56,57^, and similiarly, responses to TCRγδ ablation were subset-specific. Thus, in BTNL or SKINT-selected T cells, TCR ablation dysregulated only a few hundred genes, in contrast to the 6,337 DEGs that distinguish mouse IFNγ-producing γδ T cells from IL-17-producing counterparts^58^. Nonetheless, a small core of RNA and protein molecules, spanning discrete effectors, cell regulatory receptors and transcription factors, was affected across distinct γδ T cell types. Some were downregulated and others were upregulated, potentially offering biomarkers for TCRγδ activity and insights into evolutionarily conserved modes by which the TCR determines γδ T cell status. Additionally, TCRαβ signalling was shown to regulate tissue-resident memory (T_RM_) cell responses to TGFβ^59^, possibly reflected in TCRγδ-ablated cells by increased expression of CD103, which is regulated by TGFβ.

Although many questions remain unanswered, our results establish unique features of γδ T cell-mediated immunosurveillance, which require cancers or other lesions to be conducive to TCR function. This in turn should inform the therapeutic deployment of γδ T cells, in which innate and adaptive immune activities are evidently both TCR-dependent.

## Methods

### Mice

Adult mice of both sexes were used at 6–24 weeks of age. Mice were bred at the Francis Crick Institute (London, UK) and Animalario del Centro de Investigaciones Biológicas Margarita Salas (Madrid, Spain). All mouse strains were of C57BL/6J genetic background: *Trgc1*^−*/*−^, *Tcrd-creERT2* *Trgc1*^*fl/fl*^, *Rosa26-creERT2* *Trgc1*^*fl/fl*^, *Tcrd-creERT2* *Trgc1*^*fl/fl*^;*Rosa26-mTmG*^*fl/fl*^ (this mouse strain is the intercross of *Tcrd-creERT2* and *Rosa26-mTmG*^*fl/fl*^, which enables the γδ T cell-specific expression of EGFP after tamoxifen administration), and Nur77-GFP reporter mice (*Nr4a1*^*GFP*^), which was used to assess antigen receptor signalling activity in vivo through GFP expression downstream of TCR activation. Mice were housed under specific pathogen-free conditions in a 12 h light:12 h dark cycle at an ambient temperature of 20–24 °C and relative humidity of 45–65%. Mice were kept in filter-topped cages with sterilized food and water ad libitum and autoclaved corncob bedding that was changed at least once weekly. All experiments were performed according to UK and Spain animal protection laws.

### Conditional Cγ1 deletion

To induce conditional deletion of *Cγ1*, *Trgc1*^*fl*^ mice were crossed with two different lines expressing tamoxifen-inducible Cre recombinase. *Tcrd-creERT2* mice were used for *Trgc1* deletion specifically in dermal and epidermal γδ T cells, while *Rosa26-creERT2* mice allowed for ubiquitous deletion upon tamoxifen administration including the small intestine. Primer sequences for genotyping are indicated in Supplementary Table 4. Experimental mice received tamoxifen for 7 days, by intraperitoneal injection. Following treatment, epidermis and dermis samples were collected from *Tcrd-creERT2* mice, whereas small intestinal IELs were isolated from *Rosa26-creERT2* mice for further analysis.

### Generation of human γδ T cell lines

Peripheral blood mononuclear cells (PBMCs) from a healthy male donor were isolated by centrifugation on a Ficoll-Paque PLUS (GE Healthcare) gradient. Cells were stained with an anti-TCR V delta 1 Monoclonal Antibody (TS8.2 clone from Invitrogen) and Vδ1^+^ cells isolated by fluorescence-activated cell sorting (FACS)-sorting with a FACSAria Fusion flow cytometer (BD Biosciences) at Flow Cytometry Facilities at Centro de Investigaciones Biológicas Margarita Salas (Madrid, Spain). First, polyclonal Vδ1^+^ cells were stimulated at day 0 with 1 mg ml^−1^ phytohemagglutinin-L (PHA-L) from Phaseolus vulgaris (Sigma-Aldrich), and co-cultured with irradiated allogeneic feeder cells weekly (PBMCs and Epstein Barr virus-transformed B cells, 40 and 65 Gy, respectively) at 1:2 ratio in Iscove’s Modified Dulbecco’s Medium (IMDM) (GE Healthcare) supplemented with 40 IU ml^−1^ recombinant human IL-2, 10% AB+ human serum, and 1% l-glutamine and Antibiotic–Antimycotic (Life Technologies). After 3 weeks of allogeneic stimuli, 0,7 million expanded Vδ1^+^ cells were stimulated with 10 μg ml^−1^ PHA-L (only at day 0) for 24 h and co-cultured with irradiated (150 Gy) HTLV1-producing cell line (MT2) at 1:1 ratio in RPMI 1640 medium from Lonza, supplemented with 100 IU ml^−1^ recombinant interleukin-2 (rIL-2), 10% fetal bovine serum and 1% l-glutamine and antibiotics-antimycotic. After 4 weeks, cell lines phenotype was checked to be exclusively Vδ1^+^ cells by flow cytometry with a FACSCelesta flow cytometer (BD Biosciences) at the Center for Cytometry and Fluorescence Microscopy of Complutense University of Madrid (Spain).

### CRISPR RNP nucleofection and human TCR deletion

Human blood HTLV1-immortalized Vδ1^+^ T cells were cultured in complete RPMI medium (RPMI 1640 with 10% heat-inactivated fetal bovine serum (FBS), 10 mM HEPES, 1× non-essential amino acids (NEAA), 2 mM l-glutamine, 1 mM sodium pyruvate, 50 μM β-mercaptoethanol, 100 U ml^−1^ penicillin and 100 μg ml^−1^ streptomycin) supplemented 100 U ml^−1^ of rhIL-2 (PeproTech, 200-02) and subjected to CRISPR to delete *TRDV1* gene using a modified protocol published previously^60^. The *TRDV1* gene was independently targeted by two different custom designed gRNAs (5′-CATCAGAACCCTGGCGAATA-3′, 5′-ACCACCAACTTGTTTCATAC-3′) synthesized by IDT. In brief, Cas9 RNPs were assembled by complexing two-component 40 μM gRNA (CRISPR RNA (crRNA):*trans*-activating crRNA (tracrRNA)) with 13 μM Cas9–GFP nuclease (IDT, 10008100) to form an RNP at 20 μM immediately before experiments. Nucleofection was carried out on 1 × 10^6^ cells with 3 μl of Cas9 RNP and P3 buffer (Lonza, V4XP-3032) in a total of 20 μl per reaction using the Lonza Nucleofection system with pulse code EH100. Immediately after nucleofection, 80 μl of pre-warmed complete RPMI medium without penicillin-streptomycin was added directly to the cells in the nucleofection cuvettes and were rested for 10 min at 37 °C. After 10 min, cells were transferred to 48-well cell culture plates and incubated overnight at 37 °C, 5% CO_2_ in complete RPMI medium containing 100 U ml^−1^ of rhIL-2 but without penicillin-streptomycin. After 24 h, cells were sorted based on GFP signal using BD FACS Aria III Cell Sorter (BD) to enrich for transfected GFP^+^ cells and returned to culture. Following 10 days, *TRDV1*-knockout efficiency was determined by flow cytometry with TCR Vδ1 antibody (Miltenyi Biotec, 130-118-968) using the BD LSR Fortessa instrument (BD).

### Isolation of mouse epidermal cells

Whole trunk epidermis was separated from the dermis by incubating skin samples in 2 mg ml^−1^ Dispase II at 37 °C for 2 h (ref. ^61^). Following incubation, the epidermal sheets were carefully peeled off and digested with 0.1 mg ml^−1^ of DNase I at 37 °C for 15 min shaking at 230 rpm to obtain single-cell suspensions for subsequent analysis.

### Isolation of mouse dermal cells

After epidermis separation, dermis tissue was finely minced with scissors and incubated at 37 °C for 1 h with shaking at 700 rpm on a Thermoblock in digestion solution containing 2.7 mg ml^−1^ collagenase XI, 0.25 mg hyaluronidase, and 0.1 mg ml^−1^ DNase I in RPMI with 1× HEPES. Digested tissue was then filtered through a 30-μm filter cone (CellTrics 30 μm, 04-0042-2316, Sysmex) into a new tube for subsequent analysis.

### Isolation of mouse intestinal IELs

Mouse IELs were isolated from small intestine^21^. In brief, small intestine was opened, washed in PBS, cut into 0.5-cm-long pieces and incubated at room temperature in complete RPMI supplemented with 1 mM DTT. Tissues were then subjected to two rounds of washes with complete RPMI, vortex and filtered through 70-μm cell strainers. The IEL fraction was then purified by Percoll density gradient (80:40:20) centrifugation and stained by flow cytometry.

### Spleen, lymph nodes and thymus cells isolation

After spleen, thymus and lymph node dissection, tissue was mechanically disaggregated and filtered through 40-μm cell strainers to obtain single-cell suspensions. Red blood cells were lysed with red blood lysis buffer for 5 min at room temperature. Lymphocytes were then washed with complete RPMI and stained for flow cytometry.

### Isolation of primary DETC lines

Primary DETCs were isolated and grown as previously described^62^. After approximately 1 month, cells were assessed for purity by flow cytometry. Cell lines with ≥85% DETCs (short-term DETCs) were used for experiments.

### Flow cytometry

To analyse γδ T cell populations, separate tissue-specific cell suspensions were prepared as described above from ears or back skin or gut of individual mice. γδ T cells were blocked with normal hamster IgG plus anti-FcR (2.4G2) and stained with specific antibodies (Supplementary Table 5). For mouse and human experiments, all commercial antibodies were used at a dilution of 1:100. In-house generated antibody was used at a dilution of 1:200. Isotype-matched control antibodies were used at the same concentrations as test antibodies. Analysis was performed with a LSR Fortessa (BD Biosciences) with electronic gates set on live cells by a combination of forward and side light scatter and viability dye exclusion. A minimum of 1,000 live events were collected per sample and data were analysed with FlowJo v10 software (BD). The anti-TCRγδ antibody clone 17D1 was used as previously described^61^ to assess Vγ5 epidermal γδ T cells. For spectral analysis, flow cytometry was performed using a Spectral Aurora flow cytometer (Cytek Biosciences, 5-laser system), capable of full-spectrum detection across UV (355 nm), violet (405 nm), blue (488 nm), yellow-green (561 nm) and red (640 nm) lasers. Fluorescence signals were recorded using the Cytek SpectroFlo software. Single-stained controls were included for spectral unmixing, using either compensation beads (UltraComp eBeads, Thermo Fisher) or cells. Negative and fluorescence-minus-one (FMO) controls were included to define gating strategies. Flow cytometry data were acquired using FACSDiva (9.2) with gating performed on live, singlet populations based on FSC-A versus FSC-H and viability dye exclusion. Dimensionality reduction techniques, including *t*-SNE were applied to visualize high-dimensional datasets using FlowJo v10 (BD Biosciences).

### FACS for bulk RNA-seq and ATAC-seq

To isolate mouse γδ T cell populations, separated tissue-specific cell suspensions were prepared as described above, incubated with antibodies for 30 min at 4 °C in FACS buffer (0.5% FBS, 2 mM EDTA in PBS, pH 7.4), then washed twice in FACS buffer. Viable γδ T cell cells were sorted into PBS buffer + 0.04% BSA and retained on ice. Human HTLV1-immortalized Vδ1^+^ were stained with TCR Vδ1 antibody (Miltenyi Biotec, 130-118-968) and sorted into TCR^+^ and TCR^−^ cells. Sorted cells were confirmed to be >85% to 95% pure prior to RNA extraction.

### In vitro stimulation assays

For analysis of in vitro production of IFNγ and IL-17 by intracellular flow cytometry, tissue-specific γδ T cells were purified after tissue preparations by FACS as describe above and were then cultured in complete RPMI medium and stimulated for 5 h with 25 ng ml^−1^ phorbol 12-myristate 13-acetate (PMA) and 1 µg ml^−1^ Ionomycin in the presence of 10 µg ml^−1^ brefeldin A (BFA) at 37 °C, 5% CO_2_. PMA, Ionomycin and BFA were all purchased from Sigma. For cytokine stimulation, sorted cells were stimulated in complete RPMI with IL-1β (10 ng ml^−1^) plus IL-23 (50 ng ml^−1^) or with IL-12 (100 ng ml^−1^) plus IL-18 (100 ng ml^−1^) for 21 h. BFA (10 µg ml^−1^) was added for the final 5 h, and production of IFNγ or IL-17 was immediately assessed by intracellular cytometry. IL-1β, IL-23, IL-12 and IL-18 were purchased from R&D and resuspended following manufacturer’s instructions.

### RT–qPCR

Epidermis from back skin was separated from dermis as described above. To isolate specific skin γδ T cell populations, cell suspensions were prepared as described in ‘Flow cytometry’. Samples were directly frozen in RLT buffer prior to RNA purification with DNase digest (RNeasy kit; QIAGEN). cDNA was generated using Superscript-II (Invitrogen, Thermo Fisher Scientific) and analysed using SYBR-green assay (Invitrogen) using a QuantStudio3 Real-time PCR machine (Applied Biosystems, Thermo Fisher Scientific). cDNA was analysed for TCR related genes by combining forward primers specific for each of the Vγ gene segment (*Trgv4*, *Trgv5* or *Trgv6*) with a reverse primer specific for *Trgc1* (or *Trgc3*). Expression levels of each gene were normalized to cyclophilin A (*Ppia*) by the d*C*t method and showed as average d*C*t of technical replicates per sample. Primer sequences are indicated in Supplementary Table 4.

### Apoptosis and necrosis detection assay

Apoptosis and necrosis were evaluated using the Apoptosis/Necrosis Detection Kit following the manufacturer’s instructions (Abcam, ab176749). In brief, cells were collected, washed with PBS, and resuspended in the assay buffer provided with the kit. Cells were then incubated with Apopxin Green Indicator, which labels phosphatidylserine exposed on apoptotic cells, together with the CytoCalcein Violet 450 viability dye and 7-AAD necrosis indicator for 30 min at room temperature protected from light. Following staining, samples were analysed by flow cytometry. Live cells were identified as CytoCalcein Violet 450^+^ Apopxin Green^−^ 7-AAD^−^, apoptotic cells as Apopxin Green^+^ 7-AAD^−^, and necrotic/late-apoptotic cells as 7-AAD^+^. Debris and doublets were excluded based on forward and side scatter characteristics.

### Confocal microscopy

Whole depilated split ear sheets were flattened in a histology cassette and fixed with 10% neutral-buffered formalin for 2 h at room temperature and permeabilized in 0.5% Triton X-100 for at least 1 h at room temperature. The epidermal sheets were blocked for 30 min with PBS containing 5% normal donkey serum (blocking buffer) and then incubated with primary antibodies overnight at room temperature in blocking buffer. *z*-sections were acquired on a Leica SP8 confocal microscope and processed and analysed using Fiji (2.3.0/1.53q)^63^. Morphological parameter sphericity was calculated as the ratio of cell object border length to its volume, ranging from 0 to 1 with higher values corresponding to more spherical objects. For analysis of mTmG, thicker intestine sections were fixed with 4% paraformaldehyde overnight at at 4 °C overnight and were then included in 4% low-melting temperature agarose (Invitrogen). Sections of 80 μm were obtained with a Leica VT1200/VT1200 S vibratome and embedded in FluoromountG (ThermoFisher Scientific). GFP was acquired on a Leica SP8 confocal microscope (see above). For thinner gut mTmG sections, after overnight fixation guts were included and frozen in OCT prior cryoprotection with 30% sucrose. Using a Cryostat Leica CM3050S guts were sectioned at 20 um and embedded in FluoromountG. Tomato and GFP was acquired using the same confocal microscope.

### TEWL measurement

Under inhalation anaesthesia, TEWL of the ear pinnae was measured using a Tewameter TM300 probe (Ck Electronic) under constant temperature and humidity conditions. Values were recorded as the average of ten consecutive measurements once readings were stabilized using MPA software (Ck Electronic).

### Ultraviolet radiation model

Under inhalation anaesthesia, mice were positioned under a cardboard box, and one or both ears were exposed and fixed flat using Blu-Tac. UVB radiation (302 nm) was administered to dorsal ear skin for a dose of 45 mJ cm^–2^ (C57BL/6) using an XX-15 Series UV bench lamp (UVP) mounted on a Perspex and plastic housing.

### Ear swelling measurement

Total ear thickness was calculated as the average of three measurements of the ear pinna with a micrometer (Mitutoyo) after discarding the first measurement.

### IMQ-induced psoriasis

To induce psoriasis, ears were treated daily with 5 mg Aldara cream (5% IMQ) per ear for 7 consecutive days. Ear thickness was measured using a micrometer starting 1 day before the first treatment (day 0) and set to 100%. Reddening and scaling were evaluated daily and scored. Reddening and scaling were assessed separately to calculate an average cumulative disease score. For treatment, mice were anaesthetized with isoflurane.

### Skin chemical carcinogenesis

DMBA and TPA were purchased from Sigma and dissolved in acetone or 100% ethanol respectively. For cutaneous carcinogenesis, female mice were shaved on the back using hair clippers and allowed to rest for 5 days prior to DMBA initiation. DMBA (300 nmol) was carefully and slowly applied by pipette, in a 75 μl volume to the entire shaved skin area. Mice were rested for 1 week and 15 nmol TPA then applied 3 times a week to the back skin. Hair regrowth during the experiment was gently removed by clipping with trimmers. Mice were monitored daily, and cutaneous tumours were counted and measured with a calliper once weekly. Back skin and tumours were evaluated by visual inspection by an observer blinded to the experimental groups.

### Epicutaneous inoculation of B16 melanoma cells

For B16 melanoma inoculation, mice were placed individually in an induction chamber, and anaesthetized with 5% isoflurane (Isoflo, Zoetis) in 100% oxygen with a delivery rate of 5 l min^−1^ until loss of righting reflex. Lubricating eye gel (Lubrithal Eye Gel 10G Tube) was applied to the eyes to prevent drying. Mice were shaved (with a Wella razor) and depilated using wecprep blades (Pilling). Mouse B16-F10 melanoma cell line was obtained, authenticated and routinely tested for mycoplasma by the Cell Services STP in the Francis Crick Institute. B16-F10 melanoma were collected by washing with PBS, incubating cells at 37 °C for 5 min with 1× trypsin/EDTA solution (0.25% Trypsin-EDTA-Sigma) and washing with Hanks’ balanced saline solution (HBSS). For epicutaneous inoculation, the stratum corneum of back skin was removed by application and removal of cellophane tape (ScotchTM) five to eight times, and the scarified site was wiped with a cotton-tipped applicator soaked in PBS. B16-F10 cells (2 × 10^5^) were suspended in 20 μl of Matrigel basement membrane matrix (Corning) and applied to the scarified region. Mice were rested for about 10 min to allow solidification of Matrigel before application of Tegaderm film (3M) over the gel. It was removed 4 days later. Developing tumours were measured using a digital calliper. Tumour growth and health status were monitored according to institutional ethical guidelines.

### Subcutaneous inoculation of B16 melanoma cells

For B16-F10 melanoma cell line inoculation, mice were placed individually in an induction chamber and anaesthetized with 5% isoflurane (Isoflo, Zoetis) in 100% oxygen at a flow rate of 5 l min^−1^ until the loss of the righting reflex. Lubricating eye gel (Lubrithal Eye Gel 10G Tube) was applied to prevent corneal drying. The injection site, typically the right flank, was shaved using a Wella razor and cleaned with 70% ethanol. B16-F10 melanoma cells were collected by washing with PBS, incubating at 37 °C for 5 min with 0.25% trypsin/EDTA solution (Sigma), and washed with Hanks’ balanced saline solution (HBSS). For subcutaneous inoculation, B16-F10 cells (1 × 10^6^) were suspended in 100 μl of PBS and injected subcutaneously using a 27G needle into the flank region. Mice were observed for 5–10 min post-injection to ensure proper distribution of the suspension and were then returned to their cages. Developing tumours were measured using a digital calliper at regular intervals. Tumour growth and health status were monitored according to institutional ethical guidelines.

### AOM plus DSS-induced colorectal tumour model

Colorectal tumorigenesis was induced by the administration of a single intraperitoneal injection of AOM (10 mg kg^−1^,13.4 M, ≥98% Sigma-Aldrich A5486). One week later, mice received 1.5% dextran sulfate sodium (DSS, molecular mass approximately 40,000, Thermo Scientific Chemicals) in drinking water for 7 consecutive days, followed by 14 days of regular water. This cycle was repeated a total of three times. Body weight, stool consistency and rectal bleeding were monitored daily to assess disease. Mice exhibiting any harmful signs of suffering such as hunched posture, piloerection, rectal bleeding and >20% body weight loss were euthanized immediately. Following the final DSS cycle, mice were maintained on regular drinking water for an additional five weeks, after which colons were collected and analysed.

### Colorectal tumour analysis

Colons were dissected, flush with cold PBS, opened longitudinally and gently flattened on a piece of filter paper with the mucosal side facing up. Tissues were then placed in 10% neutral-buffered formalin, and fixed 36 h at room temperature. First, tumours were assessed macroscopically. Each colon was then rolled tightly from the distal end towards the proximal end to form a ‘Swiss roll’. Fixed rolls were then processed for paraffin embedding, sectioned at 5 µm, and stained with haematoxylin and eosin for histological analysis.

### Histopathological examination

Four-micrometre-thick, formalin-fixed, paraffin-embedded sections from colon were stained with haematoxylin and eosin and examined by a board-certified Veterinary Pathologists (ASB). Histopathological assessment was performed blind to experimental grouping using a light microscope (Olympus BX43). Four parameters were evaluated and included epithelial hyperplasia and/or goblet cell depletion, leucocyte infiltration into the lamina propria, area affected and markers of severe inflammation, which included crypt abscess formation, submucosal leucocyte infiltration, crypt branching, ulceration and fibrosis. Each parameter was scored on a scale of 0–3 producing a total score ranging from 0 to 12. The presence of neoplasia was semi quantitatively evaluated on a scale 0 to 5 based on the type (adenoma and adenocarcinoma), severity and extension of these lesions.

### Bulk RNA-seq

cDNA was prepared from 10 ng input RNA using the NuGEN Ovation RNA-Seq System (V2), and libraries constructed using the NuGEN Ultralow Library System V2. Both these steps followed the manufacturer’s instructions. The resulting libraries were pooled for sequencing on an Illumina HiSeq 2500 platform with single-ended 75-bp reads.

### Bulk ATAC-seq

ATAC-seq samples were prepared using the ATAC-seq Kit from Active Motif (53150) following the manufacturer’s instructions. For each sample, 50 000 cells were lysed in ice-cold ATAC Lysis Buffer and immediately spin down at 500*g* for 10 min at 4 °C. After supernatant removal, nuclei were resuspended in tagmentation master mix and incubated at 37 °C for 30 min while shaking at 800 rpm. Tagmentation reaction was stopped by addition of DNA Purification Binding buffer (Active Motif). Tagmented DNA fragments were purified and eluted in 35 μl of DNA Elution buffer (Active Motif), which were then amplified in a 50 μl PCR reaction using standard ATAC PCR conditions: 72 °C for 5 min; 98 °C for 30 s and thermocycling at 98 °C for 10 s, 63 °C for 30 s and 72 °C for 1 min for 10 cycles. Each 50 μl PCR reaction contained of 33.5 μl Tagmented DNA, 1 μl dNTPs (10 mM), 10 μl Q5 Reaction Buffer (5×), 0.5 μl of Q5 High-Fidelity Polymerase (2 U/μl), 2.5 μl Nextera i5 Indexed primer (25 μM) and 2.5 μl Nextera i7 Indexed primer (25 μM) (Illumina). Following amplification, ATAC-seq libraries were cleaned up using 60 μl of SPRI bead solution (1.2× the sample volume) and two 80% ethanol washes while being placed on a magnetic rack stand before being eluted in 20 μl DNA Purification Elution Buffer (Active Motif). All ATAC-seq libraries were examined on the TapeStation (Agilent) before sequenced on the NovaSeq 6000 system (Illumina), with paired-end read lengths of 50 bp and at least 50 million uniquely mapped reads per sample.

### Bulk RNA-seq bioinformatic analysis

Reads alignment and quantification: Raw reads were processed using the publicly available nf-core^64^ RNA-seq pipeline v3.3 with STAR^64^ or RSEM^65^ against mouse genome assembly GRCm38 and Ensembl transcript annotation release 95. Gene-level RSEM abundance estimates were imported into R (v4.4.3) using the DESeq2 1.46.0 package^66^. Transcript counts were normalized for downstream analyses through VST using the default function. Experimental batch effect was removed using limma 3.62.2 package. PCA was performed with VST-normalized counts using plotPCA function from DESeq2.

Differential gene expression analysis based on the negative binomial distribution with Wald test between different genotypes was performed for each dataset using the DESeq function from DESeq2 1.46.0 package. Significance was determined by adjusted *P* value < 0.05 and a base mean >10. A combined list of significant DEGs from all pairwise comparisons was created and used to identify the number of genes upregulated (log_2_ fold change >0) and downregulated (log_2_ fold change <0), and to generate heat maps comparing VST-normalized counts across samples with Euclidean distance clustering using the pheatmap 1.0.12 package.

GSEA was performed using the fgsea 1.32.2 package^67^. The analysis was performed on a ranked gene list generated using their level of differential expression (*P* value) and their log_2_ fold change to score magnitude and direction of change. Gene sets were retrieved from the Molecular Signatures Database (MSigDB) using their mouse and human Hallmarks collections with the msigdbr 10.0.1package [https://igordot.github.io/msigdbr/]. Additional R packages used for data analysis and plotting include tidyverse 2.0.0, org.Mm.eg.db 3.20.0, ggrepel 0.9.6, magrittr 2.0.3, ggplot2 3.5.1, and RcolorBrewer 1.1-3. All the code to analyse RNA-seq datasets are available at https://github.com/FrancisCrickInstitute/Cg1-KO.

### Bulk ATAC-seq bioinformatic analysis

For read alignment and peak calling, sequencing reads were processed using the nf-core ATAC-seq pipeline (v2.1.2)^64^, aligned to the mouse reference genome (mm10), and filtered using the ENCODE blacklist to exclude artefactual regions. Peak calling was performed with MACS2 in broad peak mode. Genotype-specific peak intersections were computed using bedtools intersect (v2.30.0), requiring a minimum of 50% reciprocal overlap between peaks. A peak was only considered to be genuine if present in all but one biological replicates for each genotype per organ group. Read counting was performed using featureCounts (v2.0.3) with–fracOverlap 0.2.

For differential accessibility analysis, read counts were imported into R 4.4.1 and filtered to include only standard chromosomes. GC content bias was addressed using the EDASeq package^68^, utilizing full quantile normalization within and between lanes. Offsets incorporating both library size and GC content were used in a negative binomial generalized linear model framework implemented via edgeR. Differential accessibility was assessed with a likelihood ratio test. Peaks with a false discovery rate (FDR) <0.05 were considered significantly differentially accessible. Genome browser data visualization: Genome browser tracks were generated with bigwig coverage files from RNA-seq and ATAC-seq data and bed files for ATAC-seq peaks initially visualized with Integrative Genomics Viewer (IGV) and finally plotted using the ggbio 1.54.0 package with EnsDb.Mmusculus.v79 2.99.0 as gene database. All the code to analyse ATAC-seq datasets is available at https://github.com/FrancisCrickInstitute/Cg1-KO.

### Metabolic analysis

Real-time metabolic profiling was carried out using the Seahorse Extracellular Flux Analyser system (Agilent) as previously described^69^. In brief, human Vδ1 cells were seeded at a density of 100,000 cells per well in poly-d-lysine pre-coated Seahorse XFe96 plates in XF assay buffer (Agilent) and centrifuged at 400*g* for 5 min to settle cells. Plates were then allowed to equilibrate at 37 °C in a non-CO_2_ incubator for 30 min. Mitochondrial function was assessed by measurement of OCR and ECAR under basal conditions and after sequential injection of 1 μM oligomycin, 1.5 μM carbonyl cyanide-*p*-trifluoromethoxyphenylhydrazone (FCCP), 100 nM rotenone and 1 μM antimycin A. To assess contribution of FAO to basal mitochondrial oxygen consumption, 300 μM etomoxir was injected in port A prior to injections of 1 μM oligomycin, 1.5 μM FCCP, 100 nM rotenone and 1 μM antimycin A. Data was acquired using the Seahorse XFe96 Extracellular Flux Analyzer and Wave software (Agilent) following manufacturer recommended settings and plotted using Prism v11 software (GraphPad Software).

### Cancer cell killing assay

Cancer cell killing assays were performed using the IncuCyte Live-Cell Analysis System (Sartorius) to enable real-time monitoring of target cell viability. Cancer cell lines (HCT116, HT29 and A375) were maintained in culture with complete medium (DMEM for HCT116 and A375, or RPMI for HT29 supplemented with 10% fetal bovine serum, 100 U ml^−1^ of penicillin and 100 mg ml^−1^ of streptomycin). Cancer cell lines were authenticated and routinely tested for mycoplasma by the Cell Services STP in the Francis Crick Institute. Cancer cell lines were red-labelled to facilitate cell identification and quantification by stable transduction with a plasmid encoding mCherry and selected using puromycin (1 μg ml^−1^). Red-labelled cancer cells were purified by FACS and culture with puromycin until assayed. Target cancer cells were seeded in 96-well flat-bottom plates at a density of 2 × 10^3^ cells per well in complete growth medium and allowed to adhere overnight at 37 °C in a humidified incubator with 5% CO_2_. The following day, human γδ T cells were added to target cells at different effector-to-target (E:T) ratios (2:1, 5:1 or 10:1). Cell death was monitored using IncuCyte Cytotox Green fluorescent viability dye added at the manufacturer’s recommended concentration. Plates were placed into the IncuCyte system, and images were acquired every 3 h using a 10× objective. Phase contrast and fluorescence images were collected from 5 fields per well to ensure representative sampling and accurate quantification. Data were analysed using IncuCyte analysis software (Sartorius), and target cell area was quantified over time. All conditions were performed in technical triplicates and experiments were repeated independently at least twice. Data are presented as mean ± s.d.

### Data collection software

Conventional flow cytometry data were collected on a BD Fortessa with FACSDiva Software v9.2 (BD). Spectral flow cytometry data were collected on a Aurora flow cytometer with SpectroFlow Software v3.3.0 (Cytek). Gene expression data by quantitative PCR with reverse transcription (RT–qPCR) were collected with QuantStudio Design and Analysis Software vl.5.2 (Applied Biosystems/Thermo Fisher Scientific). Seahorse data were collected with Wave Software v2.4.3.7 (Agilent Technologies). Live imaging lncucyte data were collected with lncucyte Controller Software v2022B Rev3 (Sartorius).

### Normalization and statistical analysis

Groups were compared with Prism v11 software (GraphPad Software) using the 2-tailed unpaired Student *t-*test for comparison of 2 groups or 1-way ANOVA for comparison of 3 groups. For comparisons of three groups across time in multiple experiments, we used a linear mixed-effects model to capture experimental hierarchy on raw values, where time and sample were considered as fixed, and experiment as random effect. Dunnet’s test was used for multiple comparisons correction, where *TRDV1*-knockout cells were compared against control cells. Data are presented as each data point and mean or mean ± s.d. *P* < 0.05 was considered significant. The experiments were not randomized, and the investigators were not blinded to allocation during experiments and outcome assessment. No statistical methods were used to predetermine sample size. All experiments were performed at least twice, either with similar results obtained and representative data shown, or with pooled data shown. Graph bars with pooled data from multiple experiments include all experiments performed. Where representative histograms are shown, data reflect ≥2 independent experiments with ≥3 mice per experiment, in which similar results were obtained.

### Ethics statement

All animal experiments in the UK were approved by the Home Office. All animal experiments in Spain were approved by the Comunidad de Madrid Animal Experimentation Ethics Committee and authorized by the Dirección General de Agricultura, Ganadería y Alimentación of the Comunidad de Madrid, and were performed in accordance with the relevant national and European legislation. The collection and use of human samples were approved by the Clinical Research Ethics Committee (CEIm) of Hospital Clínico San Carlos (Madrid, Spain) and were conducted in accordance with the principles of the Declaration of Helsinki and applicable national regulations. Written informed consent was obtained from all participants prior to sample collection.

### Materials availability

Mouse lines generated in this study will be maintained in the animal house of the current institutes of A.C.H. and M.M.R. and/or stored locally as frozen embryos and can be made available on request.

### Reporting summary

Further information on research design is available in the Nature Portfolio Reporting Summary linked to this article.

## Online content

Any methods, additional references, Nature Portfolio reporting summaries, source data, extended data, supplementary information, acknowledgements, peer review information; details of author contributions and competing interests; and statements of data and code availability are available at https://doi.org/10.1038/s41586-026-11076-4.

## Supplementary information

Supplementary Information This file contains Supplementary Methods, Supplementary Table 2 (differentially expressed TCR proteins from proteomic analysis in epidermal γδ cells), Supplementary Table 4 (oligonucleotide and primer sequences) and Supplementary Table 5 (antibodies, reagents and key resources).

Reporting Summary

Supplementary Table 1 RNA-seq datasets of differential expressed genes. This file contains all the DEGs that were obtained from the bulk RNA-seq analysis following TCR deletion in different mouse tissues and human cells (each tab is a separate dataset from a specific tissue).

Supplementary Table 3 Cross-tissue concordant regulation of DEGs in gene sets significantly differentially expressed following TCR deletion. This file shows a cross-tissue comparison of DEGs following TCR deletion, illustrating common transcriptional regulation (upregulation or downregulation) for genes enriched in specific gene sets from GSEA, particularly for genes enriched in mitotic spindle and in metabolic pathways.

Peer Review File

## Source data

Source Data Fig. 1

Source Data Fig. 2

Source Data Fig. 3

Source Data Fig. 4

Source Data Fig. 5

Source Data Extended Data Fig. 1

Source Data Extended Data Fig. 2

Source Data Extended Data Fig. 3

Source Data Extended Data Fig. 4

Source Data Extended Data Fig. 5

Source Data Extended Data Fig. 6

Source Data Extended Data Fig. 7

Source Data Extended Data Fig. 8

## Data Availability

Raw and processed data for the mouse ATAC-seq and RNA-seq data generated in this paper are available at NCBI’s Gene Expression Omnibus (GEO) repository under the accession identifiers GSE277902 and GSE277903, respectively. The reference genome for mouse RNA-seq and ATAC-seq used was mm10 *Mus musculus* GRCm39 version 115, which is available from Ensembl (https://ftp.ensembl.org/pub/release-115/fasta/mus_musculus/dna/). Human RNA-seq data generated in this paper are available from GEO under accession GSE297713. The reference genome for human RNA-seq used was GRCh38 version 95, which is available from Ensembl (https://ftp.ensembl.org/pub/release-95/fasta/homo_sapiens/dna/). Source data are provided with this paper.

## Code availability

Data processing pipelines and the code to reproduce all figures for this project are publicly available on Github (https://github.com/FrancisCrickInstitute/Cg1-KO).
