# Supplementary material for: γδ T cell receptor dependencies define a unique immunosurveillance modality
Source: Nature. 2026 Sep 30;659(8140):490–501. doi: 10.1038/s41586-026-11076-4 (PMC13754573; doi:10.1038/s41586-026-11076-4)
Supplement: Supplementary file 1 [file 41586_2026_11076_MOESM1_ESM.pdf]

nature portfolio
https://doi.org/10.1038/s41586-026-11076-4

Supplementary information

γδ T cell receptor dependencies define a unique immunosurveillance modality

In the format provided by the authors and unedited

Supplementary Methods

γδ T cell receptor dependencies define a unique immunosurveillance modality

Veland, Nicolas[1, 2,10], Garcia-Cassani, Bethania[3,4,10], Zarco-Cuadrillero, Ángela[4], Mavrigiannaki, Annamaria[2], Natalini, Ambra[2,1], Eum, Josephine[1,2], Suarez-Bonnet, Alejandro[5, 6], Vantourout, Pierre.[1,2], McKenzie, Duncan R.[2], Franken, Jannik[2], Marin, Ana V.[4], Jandke, Anett[7], Andrés-Ejarque, Rosa[8], Strid, Jessica[9], Hayday, Adrian C.[1,2,8,11], and Muñoz-Ruiz, Miguel[2,4,11]

[1]Peter Gorer Department of Immunobiology, King's College London.

[2]Immunosurveillance Laboratory, The Francis Crick Institute, London, NW11AT, United Kingdom.

[3]Development and Homeostasis of the Nervous System Laboratory, The Francis Crick Institute, London, United Kingdom.

[4]Department of Immunology, Ophthalmology and Ear, Nose and Throat, Complutense University School of Medicine and 12 de Octubre Health Research Institute (imas12), Madrid, Spain.

[5]Experimental Histopathology, The Francis Crick Institute, London NW1 1AT, UK,

[6]Department of Pathobiology and Population Sciences, The Royal Veterinary College, North Mimms, Hatfield, Hertfordshire AL9 7TA, UK

[7]Departement Biomedizin DBM Hebelstrasse - FG Hess Hebelstrasse 20 4031 Basel, Switzerland.

[8]Centre for Inflammation Biology and Cancer Immunology and City of London Cancer Centre, King's College London, SE19RT, United Kingdom.

[9]Department of Immunology and Inflammation, Imperial College, London, W12 0NN, United Kingdom

[10]These authors contributed equally to this study.

[11]These authors contributed equally to this study.

Generation of floxed *Trgc1* knock out mouse model

Two guide RNAs (sgRNAs) were designed, one to a predicted cut at site chr13:19297741 upstream of exon 1 of *Trgc1* (5'-GTATATAGTCATTCGTGTAA-3') and one to a predicted cut at site chr13:19401356 (5'-CTTAAATCACAGAACTCGGG-3'). Two ssODN containing the LoxP sites were designed with 50-60bp of homology either side, were synthesised by IDT. C57BL/6J zygotes were electroporated with 50 uL of Cas9 protein/3'-sgRNA/3' LoxP ssODN (1.2 uM/6 uM/8 uM using a Nepa21 electroporator (Sonidel). Founder mice were initially screened by qPCR and a single founder mouse with a perfect integration of the 3' loxP sequence was confirmed by long-range PCR and sequencing of nested PCR amplicons using Illumina MiSeq 250 bp paired-end reads. In addition to the precise homology-directed repair (HDR)-mediated integration of the loxP site, insertions and deletions (indels) were also generated at the CRISPR/Cas9 cut sites as a result of error-prone non-homologous end joining (NHEJ), reflecting the dual repair outcomes of genome editing. The resulting mice were screened by qPCR, and a founder mouse was generated. The deletion was confirmed by Illumina MiSeq sequencing, and the founder was bred with C57BL6/J, resulting in 5 F1 mice. Oligos and primers sequences are included in Supplementary Table 4.

Ultra-low input Mass spectrometry Proteomic analysis

Epidermal sorted γδ T cells were lysed in 30 µL mild lysis buffer (50mM Tris pH:8, 100mM KCL, 0.2% triton) and 30 µL harsh lysis buffer (100 mM TEAB, 5% SDS). Samples were then sonicated and reduced using 50 mM DTT and incubated at 45 °C for 10 min. Samples were then incubated at room temperature for 5 min with 10 mM Iodoacetamide. For trypsin digestion, the PROTIFI S-Trap™ system was utilized using the S-Trap™ Micro Columns (PROTIFI, Cat. # C02-micro-80) according to the manufacturer's instructions. In brief, 5 µL of 55% phosphoric acid was added to all samples. 165 µL of 100 mM TEAB-MeOH Binding Buffer was added to one S-Trap™ Micro Column per sample. Afterwards, the whole sample mixtures were added on top of the Binding Buffer in the Micro Columns and centrifuged for 1 min at 500 g to precipitate samples on top of the S-Trap matrix. Samples were then washed four times by adding additional 165 µL of Binding Buffer to each column, centrifuged for 1 min at 500 g and discarding the flowthrough. Columns were transferred to fresh 1.5 mL Eppendorf Protein LoBind Tubes. A total of 45 µL of Digestion Buffer containing 2 mg Trypsin in TEAB was added to each column. Column in collection tubes were incubated at 45 °C for 5 hours. Following trypsin digestion, peptides were eluted from the columns using different elution buffers. First, 40 µL of Elution Buffer 1 (50 mM TEAB in H2O) was added and columns were centrifuged for 1 min at 350 g to elute peptides generally. Second, 40 µL of Elution Buffer 2 (0.1% formic acid) was added and columns were centrifuged for 1 min at 350 g to elute acidic peptides specifically. Finally, 40 µL of Elution Buffer 3 (50% acetonitrile, 0.1% formic acid) was added and columns were centrifuged for 1 min at 350 g to elute hydrophobic peptides specifically. All elution fractions were combined into the same collection tube. Samples were frozen in dry ice and dried overnight using the Labconco™ CentriVap Cold Trap at -4 °C to remove any residual acetonitrile from the samples. Samples were resuspended in 0.1 % formic acid for sample loading and mass spectrometry. Evotip Pure C18 trap columns (EVOSEP, Cat. # EV2011) were used for sample loading, according to the manufacturer's instructions, loading approximately 200 ng of peptides per sample. Data was acquired using TIMS TOF Pro2 by Bruker, coupled to Evosep One LC system. For the Liquid Chromatography (LC) separation, the standard 60SPD 2.3 method was used, and separation was performed using EV-1109 column, heated at 40° C during analyses. TIMS TOF Pro2 was operated in DIA PASEF mode, scan width was set to 100-1700 m/z with the IM 1K/0 0.6 - 1.6, ramp and accumulation time was locked at 100 ms.

Proteomic bioinformatic analysis

Following acquisition, raw data was processed using Spectronaut 19.5. For peptide identification, followed by protein identity allocation, the mouse UniProt database was used by Spectronaut. The intensity data was normalized using cross-run local normalization, which is based on a retention time dependent local regression model described in Callister et al. 2006. The data was exported to a .tsv file, followed by a custom R script for further analysis using RStudio 2024.04.2+764 with R 4.4.1. All P values were calculated using Welch's t-test. P Values were adjusted using the Benjamini-Hochberg method to adjust for multiple testing and false discovery rate. Batch correction was performed in R with the limma version 3.62.1 package.

Supplementary Table 2: Differentially expressed TCR proteins from proteomic analysis in epidermal γδ cells.

Protein ID	WT 1	WT 2	WT 3	KO 1	KO 2	KO 3	WT mean	KO mean	FC	Log2FC	pValue
TRGC1	808.209	700.322	747.643	205.471	345.572	282.144	752.058	277.729	0.369	-1.467	0.00042
TRDC	311.067	402.464	358.499	180.783	229.122	203.219	357.344	204.374	0.572	-0.805	0.00199
TRDV4	92.540	80.892	95.539	68.592	71.215	61.080	89.657	66.962	0.747	-0.420	0.01643
CD3G	181.226	256.072	260.918	178.153	159.366	126.491	232.738	154.670	0.665	-0.584	0.03075

WT: wild-type ($\gamma\delta^{TCR}$) sample; KO: knock-out ($\gamma\delta^{X}$) sample; FC: Fold Change.

Supplementary Table 4: Oligos and Primers Sequences (5' to 3')

**Mouse *Trgc1* CRISPR single stranded oligos**	
5'-loxP*Trgc1*	AATAAAGCAATGGGCACATTGTCCAAACCACAGCAGAAACATGGAAGGATAATTCCTTTAATAACTTCGTATAGCATACATTATACGAAGTTATCACGAATGACTATATACACGTACAAAAAAAAAAATAAAAGCTACTTACATCTATTTCACC
3'-loxP *Trgc1*	ACACATTCCTGTGCCAGGCGTGGTGGCATCTGCCTTAAATCACAGAACTCATAACTTCGTATAGCATACATTATACGAAGTTATGGGAGGCAGAGGCAGGGAGATTTCTGAGTTGAGGCCAGCCTGGTCTACAA
**Genotyping primers and probe sets**	
LR-F/5'-LoxP *Trgc1* MiSeq Forward	TGCTGTGAACAAGTCTGAGCT
5'-LoxP *Trgc1* MiSeq Reverse	CCCTGTCCCCCATGTGTTAC
3'-LoxP *Trgc1* MiSeq Forward	TGTCATGGTATTCGGTGACAGT
LR-R/3'-LoxP *Trgc1* MiSeq Reverse	TGATCAGGGGTTGCTTGGTG
Tcrg 5loxP-2 Fl Forward	CCACAGCAGAAACATGGAAGGATAATT
Tcrg 5loxP-2 Fl Probe	ATACATTATACGAAGTTATCACGA
Tcrg 5loxP-2 Fl Reverse	CATTTAGCTGATGCTACAATGAGCTCTG
Tcrg 5loxP-2 WT Forward	GCACATTGTCCAAACCACAGCA
Tcrg 5loxP-2 WT Probe	TCATTCGTGTAAAGGAA
Tcrg 5loxP-2 WT Reverse	GCTACAATGAGCTCTGCATTTCTTC
Tcrg 3loxP Fl Forward	GGCATCTGCCTTAAATCACAGAACT
Tcrg 3loxP Fl Probe	ATACATTATACGAAGTTATGGGAG
Tcrg 3loxP Fl Reverse	CTGGCCTCAACTCAGAAATCTCC
Tcrg 3loxP WT Forward	GTCATGGTATTCGGTGACAGTAACAAGAG
Tcrg 3loxP WT Probe	CTCCCGAGTTCTGTGAT
Tcrg 3loxP WT Reverse	CTGGCCTCAACTCAGAAATCTCC
Tcrg-WT Forward	GCCAAATAATGGGCATATTAACAAGTTCT
Tcrg-WT Probe	CTTTTAGTTGCTGTGAGT
Tcrg-WT Reverse	CCAGCAAGTTGTAGGCTTGGT
**RT-qPCR Primers**	
Cyclophilin A Forward	CAAATGCTGGACCAAACACAA
Cyclophilin A Reverse	CCATCCAGCCATTCAGTCTTG
Trgv4 Forward	TATGTCCTTGCAACCCCTACCC
Trgv5 Forward	CCTGATAATCAACAATGTTGTCAC
Trgv6 Forward	ACCAAGTGACAGAAGAGGACACG
Trgc1 Reverse	CCAATACACCCTTATAACATCT
Trgc3 Reverse	GTCCAGGATGGTATTGCCTTT

Supplementary Table 5: Antibodies, Reagents and Key Resources

**Reagent or resource**	**Source**	**Identifier**
**Antibodies**		
Alexa Fluor 405 anti-human ULBP-3 (166510)	R & D Systems	Cat. # FAB1517V, RRID: AB_3646601
Alexa Fluor 647 anti-human/mouse/rat CD278 (ICOS) (C398.4A)	BioLegend	Cat. # 313516, RRID: AB_2122582
Alexa Fluor 647 anti-mouse TCR Vγ6 (1C10-1F7)	BD Biosciences	Cat. # 570926, RRID: AB_3686117
Alexa Fluor 647 anti-mouse TCR Vγ7	In house	
Alexa Fluor 700 anti-mouse CD5 (53-7.3)	BioLegend	Cat. # 100636, RRID: AB_2687001
APC Annexin V	BioLegend	Cat. # 640941, RRID: AB_2616657
APC anti-human CD112 (Nectin-2) (TX31)	BioLegend	Cat. # 337411, RRID: AB_2565729
APC anti-human TCR Vδ1 antibody, REAfinity (REA173)	Miltenyi Biotec	Cat. # 130-118-968, RRID: AB_2733451
APC anti-mouse CD103 (2E7)	BioLegend	Cat. # 121413, RRID: AB_1227503
APC anti-mouse CD137 (17B5)	BioLegend	Cat. # 106110, RRID: AB 2564297
APC anti-mouse CD3 (17A2)	BioLegend	Cat. # 100236, RRID: AB_2561456
APC anti-mouse CD314 (NKG2D) (CX5)	BioLegend	Cat. # 130212, RRID: AB_1236372
APC anti-mouse CD45 (30-F11)	BioLegend	Cat. # 103112, RRID: AB_312977
APC anti-mouse TCR Vγ1.1/Cr4 (2.11)	BioLegend	Cat. # 141108, RRID: AB_10901177
APC anti-mouse TCR Vγ2/4 (UC3-10A6)	BioLegend	Cat. # 137708, RRID: AB_10897644
APC anti-mouse TCR Vγ4 (49.2)	BioLegend	Cat. # 160608, RRID: AB_3683175
APC anti-mouse TCR Vγ5 (536)	BioLegend	Cat. # 137506, RRID: AB_10898113
APC anti-mouse TCRβ (H57-597)	BioLegend	Cat. # 109212, RRID: AB_313435
APC anti-mouse TIGIT (Vstm3) (1G9)	BioLegend	Cat. # 142106, RRID: AB_10960139
APC/Cy7 anti-mouse CD3ε (2C11)	BioLegend	Cat. # 100330, RRID: AB_1877170
APC/Cy7 anti-mouse CD45 (30-F11)	BioLegend	Cat. # 103116, RRID: AB_312981
APC/Cy7 anti-mouse CD69 (H1.2F3)	BioLegend	Cat. # 104526, RRID: AB_10679041
APC/Cy7 anti-mouse NK-1.1 (PK136)	BioLegend	Cat. # 108724, RRID: AB_830871
APC/Fire 810 anti-mouse CD3 (17A2)	BioLegend	Cat. # 100268, RRID: AB_2876392
Brilliant Violet 421 anti-human B7-H6 (1A5)	BD Biosciences	Cat. # 569453, RRID: AB_3685076
Brilliant Violet 421 anti-human CD1d (51.1)	BioLegend	Cat. # 350315, RRID: AB_2687378
Brilliant Violet 421 anti-mouse IL-17A (TC11-18H10.1)	BioLegend	Cat. # 506925, RRID: AB_10900442

Reagent or resource	Source	Identifier
Brilliant Violet 421 anti-mouse TCRβ (H57-597)	BioLegend	Cat. # 109230, RRID: AB_2562562
Brilliant Violet 421 anti-mouse TCRγδ (GL3)	BioLegend	Cat. # 118119, RRID: AB_10896753
Brilliant Violet 421 anti-mouse Vγ1.1 TCR (2.11)	BioLegend	Cat. # 566308, RRID: AB_2739676
Brilliant Violet 421 anti-human CD3 (OKT3)	BioLegend	Cat. # 317344, RRID: AB_2565849
Brilliant Violet 510 anti-human CD335 (NKp46) (9-E2)	BD Biosciences	Cat. # 564064, RRID: AB_2738571
Brilliant Violet 510 anti-mouse CD3 (17A2)	BioLegend	Cat. # 100234, RRID: AB_2562555
Brilliant Violet 510 anti-mouse CD69 (H1.2F3)	BioLegend	Cat. # 104531, RRID: AB_2562326
Brilliant Violet 510 anti-mouse TCR Vγ4 (49.2)	BD Horizon	Cat. # 756139, RRID: AB 3688395
Brilliant Violet 510 anti-human IFN-γ (4S.B3)	BioLegend	Cat. # 502544, RRID: AB_2563883
Brilliant Violet 570 anti-mouse TCR β (H57-597)	BioLegend	Cat. # 109231, RRID: AB_10900242
Brilliant Violet 605 anti-human CD137 (4-1BB) (4B4-1)	BioLegend	Cat. # 309822, RRID: AB_2565997
Brilliant Violet 605 anti-human ULBP-2/5/6 (165903)	BD Biosciences	Cat. # 748131, RRID: AB_2872592
Brilliant Violet 605 anti-human/mouse/rat CD278 (ICOS) (C398.4A)	BioLegend	Cat. # 313538, RRID: AB_2687079
Brilliant Violet 605 anti-mouse CD103 (2E7)	BioLegend	Cat. # 121433, RRID: AB_2629724
Brilliant Violet 605 anti-mouse CD366 (Tim-3) (RMT3-23)	BioLegend	Cat. # 119721, RRID: AB_2616907
Brilliant Violet 605 anti-mouse CD45 (30-F11)	BioLegend	Cat. # 103140, RRID: AB_2562342
Brilliant Violet 605 anti-mouse TCR Vγ2/4 (UC3-10A6)	BD Biosciences	Cat. # 742310, RRID: AB_2740691
Brilliant Violet 650 anti-mouse CD223 (LAG-3) (C9B7W)	BioLegend	Cat. # 125227, RRID: AB_2687209
Brilliant Violet 711 anti-human MIC A/B (6D4)	BD Biosciences	Cat. # 742326, RRID: AB_2740705
Brilliant Violet 711 anti-mouse CD107a (LAMP-1) (1D4B)	BioLegend	Cat. # 121631, RRID: AB_2783065
Brilliant Violet 711 anti-mouse IFN-γ (XMG1.2)	BioLegend	Cat. # 505835, RRID: AB_11219588
Brilliant Violet 750 anti-human TCRβ (IP26)	BioLegend	Cat. # 306746, RRID: AB_2810463
Brilliant Violet 750 anti-mouse CD44 (IM7)	BioLegend	Cat. # 103079, RRID: AB_2941373

Reagent or resource	Source	Identifier
Brilliant Violet 785 anti-mouse CD27 (LG.3A10)	BioLegend	Cat. # 124241, RRID: AB_2800595
Brilliant Violet 786 anti-human CD366 (TIM3) (F38-2E2)	Thermo Fisher Scientific	Cat. # 417-3109-42, RRID: AB_2925738
BUV395 anti-mouse CD45 (30-F11)	BD Biosciences	Cat. # 565967, RRID: AB_2739420
BUV661 anti-mouse CD122 (5H4)	BD Biosciences	Cat. # 741537, RRID: AB_2740112
BUV737 anti-mouse Vγ2 (Vγ4) TCR (UC3-10A6)	BD Biosciences	Cat. # 748998, RRID: AB_2873395
BUV805 anti-mouse CD3 (17A2)	BD Biosciences	Cat. # 749276, RRID: AB_2740114
FITC anti-human CD3 (SK7)	BioLegend	Cat. # 344803, RRID: AB_2043992
FITC anti-human TCR Vδ1 antibody, REAfinity (REA173)	Miltenyi Biotec	Cat. # 130-120-442, RRID: AB_2752100
FITC anti-human TCR Vδ2 antibody (B6)	BioLegend	Cat. # 331405, RRID: AB_1089231
FITC anti-mouse CD19 (6D5)	BioLegend	Cat. # 115505, RRID: AB_313640
FITC anti-mouse CD279 (PD-1) (29F.1A12)	BioLegend	Cat. # 135214, RRID: AB_10680238
FITC anti-mouse CD314 (NKG2D) (C7)	BioLegend	Cat. # 115711, RRID: AB_2133291
FITC anti-mouse F4/80 (BM8)	eBioscience	Cat. # 11-4801-82, RRID: AB_2637191
FITC anti-mouse I-A/I-E (2G9)	BD Biosciences	Cat. # 553623, RRID: AB_394958
FITC anti-mouse TCR Vγ1.1/Cr4 (2.11)	BioLegend	Cat. # 141104, RRID: AB_10697031
FITC anti-mouse TCRβ (H57-597)	eBioscience	Cat. # 11-5961-85, RRID: AB_465329
FITC anti-mouse TCRγδ (GL3)	BioLegend	Cat. # 118106, RRID: AB_313830
FITC anti-human TNF-α (MAb11)	BioLegend	Cat. # 502906, RRID: AB_315258
Pacific Blue anti-human CD45 (HI30)	BioLegend	Cat. # 304029, RRID: AB_2174123
PE anti-human CD95 (Fas)	BioLegend	Cat. # 305607, RRID: AB_314545
PE anti-human TCR Vδ2, REAfinity (REA771)	Miltenyi Biotec	Cat. # 130-111-010, RRID: AB_2653972
PE anti-mouse CD103 (2E8)	BioLegend	Cat. # 121406, RRID: AB_1133989
PE anti-mouse CD122 (IL-2Rβ) (5H4)	BioLegend	Cat. # 105906, RRID: AB_2125736
PE anti-mouse CD137 (17B5)	BioLegend	Cat. # 106106, RRID: AB_2287565
PE anti-mouse CD314 (NKG2D) (CX5)	BioLegend	Cat. # 130207, RRID: AB_1227713
PE anti-mouse CD366 (Tim-3) (RMT3-23)	BioLegend	Cat. # 119704, RRID: AB_345378
PE anti-mouse TIGIT (GIGD7)	eBioscience	Cat. # 12-9501-82, RRID: AB_11042152
PE/Cy7 anti-human TIGIT (VSTM3) (A15153G)	BioLegend	Cat. # 372714, RRID: AB_2632929
PE/Cy7 anti-human NKG2D (1D11)	BioLegend	Cat. # 320812 RRID: AB_2234394

Reagent or resource	Source	Identifier
PE/Cy7 anti-mouse CD159a (NKG2AB6) (16A11)	BioLegend	Cat. # 142809, RRID: AB_2728160
PE/Cy7 anti-mouse CD3 (17A2)	BioLegend	Cat. # 100220, RRID: AB_1732057
PE/Cy7 anti-mouse CD366 (Tim-3) (RMT3-23)	BioLegend	Cat. # 119716, RRID: AB_2571932
PE/Cy7 anti-mouse CD69 (H1.2F3)	BioLegend	Cat. # 104512, RRID: AB_493564
PE/Cy7 anti-mouse IFN-γ (XMG1.2)	BioLegend	Cat. # 505825, RRID: AB_1595591
PE/Cy7 anti-mouse TCR γ/δ (GL3)	BioLegend	Cat. # 118124, RRID: AB_11204423
PE/Cy7 anti-mouse Vgamma2(4) TCR (UC3-10A6)	eBioscience	Cat. # 25-5828-82, RRID: AB 2573474
PE/Cy7 anti-mouse/human CD44 (IM7)	BioLegend	Cat. # 103030, RRID: AB_830787
PE/Cy7 anti-human CD155 (PVR) (SKII.4)	BioLegend	Cat. # 337614, RRID: AB_2565747
PE/Cy7 anti-human TCR PAN γ/δ (IMMU510)	Beckman Coulter	Cat. # B10247, RRID: AB_3662706
PerCP/Cy5.5 anti-mouse CD223 (LAG-3) (C9B7W)	BioLegend	Cat. # 125212, RRID: AB_2561517
PerCP/Cy5.5 anti-mouse CD45 (30-F11)	BioLegend	Cat. # 103132, RRID: AB_893340
PerCP/Cy5.5 anti-mouse IL-17A (TC11-18H10.1)	BioLegend	Cat. # 506919, RRID: AB_469456
PerCP/Cyanine5.5 anti-human IL-13 (JES10-5A2)	BioLegend	Cat. # 501911, RRID: AB_2124284
PerCP/eFluor 710 CD278 (ICOS) (7E.17G9)	eBioscience	Cat. # 46-9942-82, RRID: AB_2744728
PE/Vio700 anti-human CD337 (NKp30) (AF29-4D12)	Miltenyi Biotec	Cat. # 130-112-432, RRID: AB_2657625
Purified rat IgG2b, κ isotype control (RTK4530)	BioLegend	Cat. # 400601, RRID: AB_326545
Anti-human XCL1+XCL2 antibody (EPR26181-30)	Abcam	Cat. # ab302522, RRID: -
**Chemicals, peptides, and recombinant proteins**		
Recombinant Mouse IL-18 (carrier-free)	BioLegend	Cat. # 767004
Recombinant Mouse IL-12	R&D Systems	Cat. # 419-ML-050
Mouse IL-23 Recombinant Protein 10ug	eBioscience	Cat. # 14-8231-63
Recombinant Mouse IL-1 beta/IL-1F2 Protein	R&D Systems	Cat. # 401-ML-025
FBS, heat inactivated	Gibco (Thermo Fisher Scientific)	Cat. # 10270106
Penicillin-streptomycin	Sigma-Aldrich	Cat. # P4333
RPMI 1640	Gibco	Cat. # 21875-091

Reagent or resource	Source	Identifier
LIVE/DEAD Fixable Blue Dead Cell Stain Kit	Thermo Fisher Scientific	Cat. # L34962
Zombie NIR Fixable Viability Kit	BioLegend	Cat. # 423106
DAPI Staining Solution	Miltenyi Biotec	Cat. # 130-111-570
Zenon Rabbit IgG Labeling Kit	Thermo Fisher Scientific	Cat. # Z25308
Red Blood Cell Lysing Buffer Hybri-Max	Sigma-Aldrich	Cat. # R7757
4-OH tamoxifen	Sigma-Aldrich	Cat. # H7904
Tamoxifen	Sigma-Aldrich	Cat. # T5648-5G
10% neutral buffered formalin (NBF)	CellPath	Cat. # BAF-9000-08A
Trypsin from bovine pancreas	Sigma-Aldrich	Cat. # T1005
DNase I from bovine pancreas	Sigma-Aldrich	Cat. # 11284932001
Trypsin/EDTA solution	Gibco	Cat. # 25200-056
HBSS	Thermo Fisher Scientific	Cat. # 14175095
Collagenase type IV	Worthington	Cat. # LS004188
DNAse I	Sigma-Aldrich	Cat. # D5025-15
BSA	Sigma-Aldrich	Cat. # A4503
Dispase II	Sigma-Aldrich	Cat. # D4693
Phorbol 12-myristate 13-acetate (PMA)	Sigma-Aldrich	Cat. # P8139
Ionomycin	Sigma-Aldrich	Cat. # I9657
Brefeldin A	Sigma-Aldrich	Cat. # B7651
**Deposited data**		
Mouse ATAC-seq data	This paper	GEO: GSE277902
Mouse RNA-seq data	This paper	GEO: GSE277903
Human RNA-seq data	This paper	GEO: GSE297713
**Experimental models: organisms/strains**		
Mouse: B6.129S-Tcrdtm1.1(Cre/ERT2)Zhu/J	The Francis Crick Institute	Cat. # JAX:031679, RRID: IMSR_JAX:031679
Mouse: B6.129-Gt(ROSA)26Sortm1(Cre/ERT2)Tyj/J	The Francis Crick Institute	Cat. # JAX: 008463, RRID: IMSR_JAX:008463
Mouse: Gt(ROSA)26Sortm4(ACTB-TdTomato,-EGFP)Luo/J	The Francis Crick Institute	Cat. # JAX: 007576, RRID: IMSR_JAX:007576
**Software**		
ImageJ	Developer: Wayne Rasband (NIH)	https://imagej.nih.gov/ij/, RRID: SCR_003070

Reagent or resource	Source	Identifier
GraphPad Prism version 11.0.0	GraphPad Inc	RRID: SCR_002798
FlowJo version 10	BD	RRID: SCR_008520
SpectroFlo	Cytek Biosciences	SpectroFlo® software (Cytek Biosciences, Fremont, CA, USA)
R (for bioinformatics analysis)	R Foundation for Statistical Computing	https://www.r-project.org/, RRID: SCR_001905

*APC, Allophycocyanin; Cy, cyanine; FITC, fluorescein isothiocyanate; PE, phycoerythrin; PerCP, peridinin chlorophyll protein; BUV, Brilliant Ultraviolet; DAPI, 4′,6-diamidino-2-phenylindole; GEO, Gene Expression Omnibus; RRID, Research Resource Identifier.

**BD: BD Biosciences (US); BioLegend: BioLegend (US); eBioscience: Thermo Fisher Scientific (UK); Thermo Fisher: Thermo Fisher Scientific (US); Worthington: Worthington Biochemical Corporation (US); Peprotech: PeproTech, Inc. (Rocky Hill, NJ, US); ImmunoTools: ImmunoTools GmbH (Gladiolenweg, Germany); Corning: Corning Inc. (US); Miltenyi Biotec: Miltenyi Biotec GmbH (Germany); Sigma-Aldrich: Sigma-Aldrich (US); R&D Systems: R&D Systems, Inc. (US); Jackson Laboratory: The Jackson Laboratory (US); Cytek Biosciences: Cytek Biosciences (Fremont, CA, US); Gibco: Gibco, a brand of Thermo Fisher Scientific (US).
