# Supplementary material for: γδ T cell receptor dependencies define a unique immunosurveillance modality
Source: Nature. 2026 Sep 30;659(8140):490–501. doi: 10.1038/s41586-026-11076-4 (PMC13754573; doi:10.1038/s41586-026-11076-4)
Supplement: Supplementary file 2 [file 41586_2026_11076_MOESM2_ESM.pdf]

Corresponding author(s): Adrian C. Hayday and Miguel Munoz-Ruiz

Last updated by author(s): 22 July, 2026

Reporting Summary

Nature Portfolio wishes to improve the reproducibility of the work that we publish. This form provides structure for consistency and transparency in reporting. For further information on Nature Portfolio policies, see our Editorial Policies and the Editorial Policy Checklist.

Statistics

For all statistical analyses, confirm that the following items are present in the figure legend, table legend, main text, or Methods section.

n/a	Confirmed	
☐	☒	The exact sample size (*n*) for each experimental group/condition, given as a discrete number and unit of measurement
☐	☒	A statement on whether measurements were taken from distinct samples or whether the same sample was measured repeatedly
☐	☒	The statistical test(s) used AND whether they are one- or two-sided<br>*Only common tests should be described solely by name; describe more complex techniques in the Methods section.*
☐	☒	A description of all covariates tested
☐	☒	A description of any assumptions or corrections, such as tests of normality and adjustment for multiple comparisons
☐	☒	A full description of the statistical parameters including central tendency (e.g. means) or other basic estimates (e.g. regression coefficient) AND variation (e.g. standard deviation) or associated estimates of uncertainty (e.g. confidence intervals)
☐	☒	For null hypothesis testing, the test statistic (e.g. *F*, *t*, *r*) with confidence intervals, effect sizes, degrees of freedom and *P* value noted<br>*Give P values as exact values whenever suitable.*
☒	☐	For Bayesian analysis, information on the choice of priors and Markov chain Monte Carlo settings
☒	☐	For hierarchical and complex designs, identification of the appropriate level for tests and full reporting of outcomes
☒	☐	Estimates of effect sizes (e.g. Cohen's *d*, Pearson's *r*), indicating how they were calculated

Our web collection on statistics for biologists contains articles on many of the points above.

Software and code

Policy information about availability of computer code

Data collection

Conventional flow cytometry data were collected on a BD Fortessa with FACSDiva Software v9.2 (BD). Spectral flow cytometry data were collected on a Aurora flow cytometer with SpectroFlow Software v3.3.0 (Cytek). Gene expression data by RT-qPCR were collected with QuantStudio Design & Analysis Software vl.5.2 (Applied Biosystems/Thermo Fisher Scientific). Seahorse data were collected with Wave Software v2.4.3.7 (Agilent Technologies). Live imaging lncucyte data were collected with lncucyte Controller Software version 2022B Rev3 (Sartorius).

Data analysis

Data processing pipelines and the code to reproduce all figures for this project will be made publicly available on Github (https://github.com/FrancisCrickInstitute/Cgl-KO) after publication.

For bulk RNA-sequencing analysis, raw reads were processed using the publicly available nf-core RNAseq pipeline v3.3 with the STAR/RSEM against mouse genome assembly GRCm38 and Ensembl transcript annotation release 95. Gene-level RSEM abundance estimates were imported into R (version 4.4.3) using the DESeq2 1.46.0 package. Transcript counts were normalised for downstream analyses through variance-stabilised transformation (VST) using the default function. Experimental batch effect was removed using limma 3.62.2 package. Principal Component Analysis (PCA) was performed with VST normalized counts using plotPCA function from DESeq2.
Differential gene expression analysis based on the negative binomial distribution with Wald test between different genotypes was performed for each dataset using the DESeq function from DESeq2 1.46.0 package. Significance was determined by adjusted P value< 0.05 and a base mean> 10. A combined list of significantly differentially expressed genes from all pairwise comparisons was created and used to identify the number of genes upregulated (log2 fold change> 0) and down regulated (log2 fold change< 0), and to generate heatmaps comparing VST normalized counts across samples with Euclidean distance clustering using the pheatmap 1.0.12 package.
Gene Set Enrichment Analysis (GSEA) was performed using the fgsea 1.32.2 package. The analysis was performed on a ranked gene list generated using their level of differential expression (P value) and their log2 fold change to score magnitude and direction of change. Gene

sets were retrieved from the Molecular Signatures Database (MSigDB) using their mouse and human Hallmarks collections with the msigdbr 10.0.lpackage [https://igordot.github.io/msigdbr/]. Additional R packages used for data analysis and plotting include tidyverse 2.0.0, org.Mm.eg.db 3.20.0, ggrepel 0.9.6, magrittr 2.0.3, ggplot2 3.5.1, and RcolorBrewer 1.1-3.

ATAC-sequencing reads were processed using the nf-core ATAC-seq pipeline (v2.1.2), aligned to the mouse reference genome (mmlO), and filtered using the ENCODE blacklist to exclude artefactual regions. Peak calling was performed with MACS2 in broad peak mode. Genotype specific peak intersections were computed using bedtools intersect (v2.30.0), requiring a minimum of 50% reciprocal overlap between peaks. A peak was only considered to be genuine if present in at least all but one biological replicates for each genotype per organ group. Read counting was performed using featureCounts (v2.0.3) with --fracOverlap 0.2.
For differential accessibility analysis, read counts were imported into R 4.4.1 and filtered to include only standard chromosomes. GC content bias was addressed using the EDASeq package, employing full quantile normalization within and between lanes. Offsets incorporating both library size and GC content were used in a negative binomial generalized linear model framework implemented via edgeR. Differential accessibility was assessed with a likelihood ratio test. Peaks with an FDR< 0.05 were considered significantly differentially accessible.
Genome browser tracks were generated with bigwig coverage files from RNA-seq and AT AC-seq data and bed files for ATAC-seq peaks initially visualized with Integrative Genomics Viewer (IGV) and finally plotted using the ggbio 1.54.0 package with EnsDb.Mmusculus.v79 2.99.0 as gene database.

Proteomic raw data was processed using Spectronaut 19.5. For peptide identification, followed by protein identity allocation, the mouse UniProt database was used by Spectronaut. The data was exported to a .tsv file, followed and further analysis using RStudio 2024.04.2+764 with R 4.4.1. All P values were calculated using Welch's t-test. P Values were adjusted using the Benjamini-Hochberg method to adjust for multiple testing and false discovery rate. Batch correction was performed in R with the limma version 3.62.1 package.

Confocal images were analysed using Fiji version: 2.3.0/1.53q, and further quantitative analysis using Definiens Developer XD software (Munich, Germany).

Flow cytometry data, including conventional flow cytometry data and spectral flow cytometry data was analysed with FlowJo v10 software (BD Biosciences, Franklin Lakes, NJ).

Metabolic analysis data was acquired using the Seahorse XFe96 Extracellular Flux Analyzer and Wave Software v2.4.3.7 (Agilent Technologies) following manufacturer recommended settings and plotted using Prism version 11 software (GraphPad Software).

Live-cell imaging analysis of cancer cell killing activity was performed using lncucyte Controller Software version 2022B Rev3 (Sartorius), and mCherry fluorescent signal as target cell area was quantified over time.

Additional statistical analysis was performed using Prism version 11 software (Graph Pad Software, San Diego, Calif).

For manuscripts utilizing custom algorithms or software that are central to the research but not yet described in published literature, software must be made available to editors and reviewers. We strongly encourage code deposition in a community repository (e.g. GitHub). See the Nature Portfolio guidelines for submitting code & software for further information.

Data

Policy information about availability of data

All manuscripts must include a data availability statement. This statement should provide the following information, where applicable:

- Accession codes, unique identifiers, or web links for publicly available datasets
- A description of any restrictions on data availability
- For clinical datasets or third party data, please ensure that the statement adheres to our policy

Raw and processed data for the mouse ATAC-seq and RNA-seq data generated in this paper are available at NCBI's Gene Expression Omnibus (GEO) repository under the accession identifiers GSE277902 and GSE277903 respectively. The reference genome for mouse RNA-seq and ATAC-seq used was mm10 == Mus_musculus.GRCm39 version 115, which is available from Ensembl [https://ftp.ensembl.org/pub/release-115/fasta/mus_musculus/dna/]. Human RNA-seq data generated in this paper is available at GEO repository under the accession identifier GSE297713. The reference genome for human RNA-seq used was GRCh38 version 95, which is available from Ensembl [https://ftp.ensembl.org/pub/release-95/fasta/homo_sapiens/dna/].

Research involving human participants, their data, or biological material

Policy information about studies with human participants or human data. See also policy information about sex, gender (identity/presentation), and sexual orientation and race, ethnicity and racism.

Reporting on sex and gender	N/A
Reporting on race, ethnicity, or other socially relevant groupings	N/A
Population characteristics	N/A
Recruitment	N/A
Ethics oversight	N/A

Note that full information on the approval of the study protocol must also be provided in the manuscript.

Field-specific reporting

Please select the one below that is the best fit for your research. If you are not sure, read the appropriate sections before making your selection.

☒ Life sciences ☐ Behavioural & social sciences ☐ Ecological, evolutionary & environmental sciences

For a reference copy of the document with all sections, see nature.com/documents/nr-reporting-summary-flat.pdf

Life sciences study design

All studies must disclose on these points even when the disclosure is negative.

Sample size	No formal statistical method was used to predetermine sample size for the RNA-seq or ATAC-seq experiments. Sample sizes were selected based on established practice in the field, previous published studies using comparable experimental designs, and the availability of biological material. Independent biological replicates were included for each experimental group to ensure reproducibility and to enable robust statistical analysis of differential gene expression and chromatin accessibility using established methods (e.g., DESeq2). The number of biological replicates provided sufficient statistical power to identify reproducible changes while accounting for biological variability between samples.<br>No formal statistical method was used to predetermine sample sizes for experiments other than RNA-seq and ATAC-seq. Sample sizes were chosen based on previous experience with the mouse models, published studies employing similar experimental designs, and practical considerations, including animal availability and ethical principles aimed at minimizing animal use while ensuring robust and reproducible results. The numbers of biological replicates were sufficient to capture biological variability and to enable appropriate statistical comparisons between experimental groups. Independent biological replicates were analysed, and all statistical tests are described in the corresponding figure legends.
Data exclusions	No data were excluded from the analyses unless there was a clear technical issue (e.g., failed staining, high number of dead cells, or tissue contamination), in which case the criteria for exclusion were pre-established.
Replication	All experiments were independently replicated at least two times (often between three and five times) with similar results. Biological and technical replicates are reported in the figure legends. DMBA-TPA was conducted only once but with very large numbers of mice which are independent experimental units.
Randomization	Samples and animals were assigned randomly to experimental groups whenever possible. Randomization was not applied when comparing genetically defined groups or clearly distinct treatments.
Blinding	Data analysis was performed with blinding to the experimental groups whenever possible. This was specifically applied to quantification of tumours in experiments such as B16.F10 subcutaneous, B16.F10 epicutaneous, DMBA-TPA skin carcinogenesis and AOM-DSS colon carcinogenesis. In addition, blinding was applied to histopathology analysis for colitis scoring of AOM-DSS mice, and to the measurement of ear thickness under no challenge and after application of Aldara and UVR.

Reporting for specific materials, systems and methods

We require information from authors about some types of materials, experimental systems and methods used in many studies. Here, indicate whether each material, system or method listed is relevant to your study. If you are not sure if a list item applies to your research, read the appropriate section before selecting a response.

Materials & experimental systems

n/a	Involved in the study
☐	☒ Antibodies
☐	☒ Eukaryotic cell lines
☒	☐ Palaeontology and archaeology
☐	☒ Animals and other organisms
☒	☐ Clinical data
☒	☐ Dual use research of concern
☒	☐ Plants

Methods

n/a	Involved in the study
☒	☐ ChIP-seq
☐	☒ Flow cytometry
☒	☐ MRI-based neuroimaging

Antibodies

Antibodies used

Alexa Fluor 405 anti-human ULBP-3 (166510) R & D Systems Cat. # FAB1517V, RRID: AB_3646601
Alexa Fluor 647 anti-human/mouse/rat CD278 (ICOS) (C398.4A) BioLegend Cat. # 313516, RRID: AB_2122582
Alexa Fluor 647 anti-mouse TCR Vγ6 (1C10-1F7) BD Biosciences Cat. # 570926, RRID: AB_3686117
Alexa Fluor 647 anti-mouse TCR Vγ7 In house
Alexa Fluor 700 anti-mouse CD5 (53-7.3) BioLegend Cat. # 100636, RRID: AB_2687001
APC Annexin V BioLegend Cat. # 640941, RRID: AB_2616657
APC anti-human CD112 (Nectin-2) (TX31) BioLegend Cat. # 337411, RRID: AB_2565729
APC anti-human TCR Vδ1 antibody, REAfinity (REA173) Miltenyi Biotec Cat. # 130-118-968, RRID: AB_2733451

APC anti-mouse CD103 (2E7) BioLegend Cat. # 121413, RRID: AB_1227503
APC anti-mouse CD137 (17B5) BioLegend Cat. # 106110, RRID: AB 2564297
APC anti-mouse CD3 (17A2) BioLegend Cat. # 100236, RRID: AB_2561456
APC anti-mouse CD314 (NKG2D) (CX5) BioLegend Cat. # 130212, RRID: AB_1236372
APC anti-mouse CD45 (30-F11) BioLegend Cat. # 103112, RRID: AB_312977
APC anti-mouse TCR Vγ1.1/Cr4 (2.11) BioLegend Cat. # 141108, RRID: AB_10901177
APC anti-mouse TCR Vγ2/4 (UC3-10A6) BioLegend Cat. # 137708, RRID: AB_10897644
APC anti-mouse TCR Vγ4 (49.2) BioLegend Cat. # 160608, RRID: AB_3683175
APC anti-mouse TCR Vγ5 (536) BioLegend Cat. # 137506, RRID: AB_10898113
APC anti-mouse TCRβ (H57-597) BioLegend Cat. # 109212, RRID: AB_313435
APC anti-mouse TIGIT (Vstm3) (1G9) BioLegend Cat. # 142106, RRID: AB_10960139
APC/Cy7 anti-mouse CD3ε (2C11) BioLegend Cat. # 100330, RRID: AB_1877170
APC/Cy7 anti-mouse CD45 (30-F11) BioLegend Cat. # 103116, RRID: AB_312981
APC/Cy7 anti-mouse CD69 (H1.2F3) BioLegend Cat. # 104526, RRID: AB_10679041
APC/Cy7 anti-mouse NK-1.1 (PK136) BioLegend Cat. # 108724, RRID: AB_830871
APC/Fire 810 anti-mouse CD3 (17A2) BioLegend Cat. # 100268, RRID: AB_2876392
Brilliant Violet 421 anti-human B7-H6 (1A5) BD Biosciences Cat. # 569453, RRID: AB_3685076
Brilliant Violet 421 anti-human CD1d (51.1) BioLegend Cat. # 350315, RRID: AB_2687378
Brilliant Violet 421 anti-mouse IL-17A (TC11-18H10.1) BioLegend Cat. # 506925, RRID: AB_10900442
Brilliant Violet 421 anti-mouse TCRβ (H57-597) BioLegend Cat. # 109230, RRID: AB_2562562
Brilliant Violet 421 anti-mouse TCRγδ (GL3) BioLegend Cat. # 118119, RRID: AB_10896753
Brilliant Violet 421 anti-mouse Vγ1.1 TCR (2.11) BioLegend Cat. # 566308, RRID: AB_2739676
Brilliant Violet 421 anti-human CD3 (OKT3) BioLegend Cat. # 317344, RRID: AB_2565849
Brilliant Violet 510 anti-human CD335 (NKp46) (9-E2) BD Biosciences Cat. # 564064, RRID: AB_2738571
Brilliant Violet 510 anti-mouse CD3 (17A2) BioLegend Cat. # 100234, RRID: AB_2562555
Brilliant Violet 510 anti-mouse CD69 (H1.2F3) BioLegend Cat. # 104531, RRID: AB_2562326
Brilliant Violet 510 anti-mouse TCR Vγ4 (49.2) BD Horizon Cat. # 756139, RRID: AB 3688395
Brilliant Violet 510 anti-human IFN-γ (4S.B3) BioLegend Cat. # 502544, RRID: AB_2563883
Brilliant Violet 570 anti-mouse TCR β (H57-597) BioLegend Cat. # 109231, RRID: AB_10900242
Brilliant Violet 605 anti-human CD137 (4-1BB) (4B4-1) BioLegend Cat. # 309822, RRID: AB_2565997
Brilliant Violet 605 anti-human ULBP-2/5/6 (165903) BD Biosciences Cat. # 748131, RRID: AB_2872592
Brilliant Violet 605 anti-human/mouse/rat CD278 (ICOS) (C398.4A) BioLegend Cat. # 313538, RRID: AB_2687079
Brilliant Violet 605 anti-mouse CD103 (2E7) BioLegend Cat. # 121433, RRID: AB_2629724
Brilliant Violet 605 anti-mouse CD366 (Tim-3) (RMT3-23) BioLegend Cat. # 119721, RRID: AB_2616907
Brilliant Violet 605 anti-mouse CD45 (30-F11) BioLegend Cat. # 103140, RRID: AB_2562342
Brilliant Violet 605 anti-mouse TCR Vγ2/4 (UC3-10A6) BD Biosciences Cat. # 742310, RRID: AB_2740691
Brilliant Violet 650 anti-mouse CD223 (LAG-3) (C9B7W) BioLegend Cat. # 125227, RRID: AB_2687209
Brilliant Violet 711 anti-human MIC A/B (6D4) BD Biosciences Cat. # 742326, RRID: AB_2740705
Brilliant Violet 711 anti-mouse CD107a (LAMP-1) (1D4B) BioLegend Cat. # 121631, RRID: AB_2783065
Brilliant Violet 711 anti-mouse IFN-γ (XMG1.2) BioLegend Cat. # 505835, RRID: AB_11219588
Brilliant Violet 750 anti-human TCRβ (IP26) BioLegend Cat. # 306746, RRID: AB_2810463
Brilliant Violet 750 anti-mouse CD44 (IM7) BioLegend Cat. # 103079, RRID: AB_2941373
Brilliant Violet 785 anti-mouse CD27 (LG.3A10) BioLegend Cat. # 124241, RRID: AB_2800595
Brilliant Violet 786 anti-human CD366 (TIM3) (F38-2E2) Thermo Fisher Scientific Cat. # 417-3109-42, RRID: AB_2925738
BUV395 anti-mouse CD45 (30-F11) BD Biosciences Cat. # 565967, RRID: AB_2739420
BUV661 anti-mouse CD122 (5H4) BD Biosciences Cat. # 741537, RRID: AB_2740112
BUV737 anti-mouse Vγ2 (Vγ4) TCR (UC3-10A6) BD Biosciences Cat. # 748998, RRID: AB_2873395
BUV805 anti-mouse CD3 (17A2) BD Biosciences Cat. # 749276, RRID: AB_2740114
FITC anti-human CD3 (SK7) BioLegend Cat. # 344803, RRID: AB_2043992
FITC anti-human TCR Vδ1 antibody, REAfinity (REA173) Miltenyi Biotec Cat. # 130-120-442, RRID: AB_2752100
FITC anti-human TCR Vδ2 antibody (B6) BioLegend Cat. # 331405, RRID: AB_1089231
FITC anti-mouse CD19 (6D5) BioLegend Cat. # 115505, RRID: AB_313640
FITC anti-mouse CD279 (PD-1) (29F.1A12) BioLegend Cat. # 135214, RRID: AB_10680238
FITC anti-mouse CD314 (NKG2D) (C7) BioLegend Cat. # 115711, RRID: AB_2133291
FITC anti-mouse F4/80 (BM8) eBioscience Cat. # 11-4801-82, RRID: AB_2637191
FITC anti-mouse I-A/I-E (2G9) BD Biosciences Cat. # 553623, RRID: AB_394958
FITC anti-mouse TCR Vγ1.1/Cr4 (2.11) BioLegend Cat. # 141104, RRID: AB_10697031
FITC anti-mouse TCRβ (H57-597) eBioscience Cat. # 11-5961-85, RRID: AB_465329
FITC anti-mouse TCRγδ (GL3) BioLegend Cat. # 118106, RRID: AB_313830
FITC anti-human TNF-α (MAb11) BioLegend Cat. # 502906, RRID: AB_315258
Pacific Blue anti-human CD45 (HI30) BioLegend Cat. # 304029, RRID: AB_2174123
PE anti-human CD95 (Fas) BioLegend Cat. # 305607, RRID: AB_314545
PE anti-human TCR Vδ2, REAfinity (REA771) Miltenyi Biotec Cat. # 130-111-010, RRID: AB_2653972
PE anti-mouse CD103 (2E8) BioLegend Cat. # 121406, RRID: AB_1133989
PE anti-mouse CD122 (IL-2Rβ) (5H4) BioLegend Cat. # 105906, RRID: AB_2125736
PE anti-mouse CD137 (17B5) BioLegend Cat. # 106106, RRID: AB_2287565
PE anti-mouse CD314 (NKG2D) (CX5) BioLegend Cat. # 130207, RRID: AB_1227713
PE anti-mouse CD366 (Tim-3) (RMT3-23) BioLegend Cat. # 119704, RRID: AB_345378
PE anti-mouse TIGIT (GIGD7) eBioscience Cat. # 12-9501-82, RRID: AB_11042152
PE/Cy7 anti-human TIGIT (VSTM3) (A15153G) BioLegend Cat. # 372714, RRID: AB_2632929
PE/Cy7 anti-human NKG2D (1D11) BioLegend Cat. # 320812 RRID: AB_2234394
PE/Cy7 anti-mouse CD159a (NKG2AB6) (16A11) BioLegend Cat. # 142809, RRID: AB_2728160
PE/Cy7 anti-mouse CD3 (17A2) BioLegend Cat. # 100220, RRID: AB_1732057
PE/Cy7 anti-mouse CD366 (Tim-3) (RMT3-23) BioLegend Cat. # 119716, RRID: AB_2571932
PE/Cy7 anti-mouse CD69 (H1.2F3) BioLegend Cat. # 104512, RRID: AB_493564
PE/Cy7 anti-mouse IFN-γ (XMG1.2) BioLegend Cat. # 505825, RRID: AB_1595591

PE/Cy7 anti-mouse TCR γ/δ (GL3) BioLegend Cat. # 118124, RRID: AB_11204423
PE/Cy7 anti-mouse Vgamma2(4) TCR (UC3-10A6) eBioscience Cat. # 25-5828-82, RRID: AB 2573474
PE/Cy7 anti-mouse/human CD44 (IM7) BioLegend Cat. # 103030, RRID: AB_830787
PE/Cy7 anti-human CD155 (PVR) (SKII.4) BioLegend Cat. # 337614, RRID: AB_2565747
PE/Cy7 anti-human TCR PAN γ/δ (IMMU510) Beckman Coulter Cat. # B10247, RRID: AB_3662706
PerCP/Cy5.5 anti-mouse CD223 (LAG-3) (C9B7W) BioLegend Cat. # 125212, RRID: AB_2561517
PerCP/Cy5.5 anti-mouse CD45 (30-F11) BioLegend Cat. # 103132, RRID: AB_893340
PerCP/Cy5.5 anti-mouse IL-17A (TC11-18H10.1) BioLegend Cat. # 506919, RRID: AB_469456
PerCP/Cyanine5.5 anti-human IL-13 (JES10-5A2) BioLegend Cat. # 501911, RRID: AB_2124284
PerCP/eFluor 710 CD278 (ICOS) (7E.17G9) eBioscience Cat. # 46-9942-82, RRID: AB_2744728
PE/Vio700 anti-human CD337 (NKp30) (AF29-4D12) Miltenyi Biotec Cat. # 130-112-432, RRID: AB_2657625
Purified rat IgG2b, κ isotype control (RTK4530) BioLegend Cat. # 400601, RRID: AB_326545
Anti-human XCL1+XCL2 antibody (EPR26181-30) Abcam Cat. # ab302522, RRID: -

Validation

All primary antibodies are identified by their RRIDs. The manufacturer's websites were checked, and all antibodies used in this study are validated for the corresponding species and applications (flow cytometry or immunofluorescence), where indicated. Antibodies were used according to the manufacturers' recommendations. For the mouse TCR V gamma 7 antibody, which was generated in-house, out data validated its specificity when TCR deleted samples were analyzed by flow cytometry and when non-V gamma 7 T cells were included in the analysis.

Eukaryotic cell lines

Policy information about cell lines and Sex and Gender in Research

Cell line source(s)

Mouse B16.F10 and human HCT116, HT29 and A375 commercially available cell lines were obtained from the Cell Services STP in the Francis Crick Institute. We generated a human V delta 1 cell line (details for the cell line generation from one male healthy donor are included in the methods section).

Authentication

Mouse B16.F10 and human HCT116, HT29 and A375 cell lines were authenticated by the Cell Services STP in the Francis Crick Institute.

Mycoplasma contamination

Mouse B16.F10 and human HCT116, HT29 and A375 cell lines were routinely tested for mycoplasma by the Cell Services STP in the Francis Crick Institute.

Commonly misidentified lines
(See ICLAC register)

No commonly misidentified cell lines were used in this study.

Animals and other research organisms

Policy information about studies involving animals; ARRIVE guidelines recommended for reporting animal research, and Sex and Gender in Research

Laboratory animals

Adult mice were used at 6-24 weeks of age. Mice were bred at the Francis Crick Institute (London, United Kingdom) and "Animalario del Centro de Investigaciones Biológicas Margarita Salas" (Madrid, Spain). All mouse strains were of C57BL/6J genetic background: Trgc1-/-, TcrdCreERT2//Trgc1flox/flox, Rosa26CreERT2// Trgc1flox/flox, TcrdCreERT2//Trgc1flox/flox //Rosa26-mTmGflox/flox (this mouse strain is the intercross of TcrdCreERT2 and Rosa26-mTmGflox/flox which enables the γδ T cell-specific expression of EGFP after tamoxifen administration), and Nur77-GFP reporter mice (Nr4a1GFP), that was used to assess antigen receptor signalling activity in vivo through GFP expression downstream of TCR activation. Mice were housed under specific pathogen-free conditions in a 12 hour light/12 hour dark cycle at an ambient temperature of 20–24°C and relative humidity of 45–65%. Mice were kept in filter-topped cages with sterilized food and water ad libitum and autoclaved corncob bedding that was changed at least once weekly.

Wild animals

No wild animals were used in this study.

Reporting on sex

All experiments included both male and female mice in approximately equal proportions. No sex-specific differences were observed in any of the experimental outcomes.

Field-collected samples

The study did not involve samples collected from the field.

Ethics oversight

All animal experiments in the United Kingdom were approved by the Home Office. All animal experiments in Spain were approved by the Comunidad de Madrid Animal Experimentation Ethics Committee and authorized by the Dirección General de Agricultura, Ganadería y Alimentación of the Comunidad de Madrid, and were performed in accordance with the relevant national and European legislation. The collection and use of human samples were approved by the Clinical Research Ethics Committee (CEIm) of Hospital Clínico San Carlos (Madrid, Spain) and were conducted in accordance with the principles of the Declaration of Helsinki and applicable national regulations. Written informed consent was obtained from all participants prior to sample collection.

Note that full information on the approval of the study protocol must also be provided in the manuscript.

Plants

Seed stocks	*Report on the source of all seed stocks or other plant material used. If applicable, state the seed stock centre and catalogue number. If plant specimens were collected from the field, describe the collection location, date and sampling procedures.*
Novel plant genotypes	*Describe the methods by which all novel plant genotypes were produced. This includes those generated by transgenic approaches, gene editing, chemical/radiation-based mutagenesis and hybridization. For transgenic lines, describe the transformation method, the number of independent lines analyzed and the generation upon which experiments were performed. For gene-edited lines, describe the editor used, the endogenous sequence targeted for editing, the targeting guide RNA sequence (if applicable) and how the editor was applied.*
Authentication	*Describe any authentication procedures for each seed stock used or novel genotype generated. Describe any experiments used to assess the effect of a mutation and, where applicable, how potential secondary effects (e.g. second site T-DNA insertions, mosiacism, off-target gene editing) were examined.*

Flow Cytometry

Plots

Confirm that:

☒ The axis labels state the marker and fluorochrome used (e.g. CD4-FITC).

☒ The axis scales are clearly visible. Include numbers along axes only for bottom left plot of group (a 'group' is an analysis of identical markers).

☒ All plots are contour plots with outliers or pseudocolor plots.

☒ A numerical value for number of cells or percentage (with statistics) is provided.

Methodology

Sample preparation	Whole trunk epidermis was separated from the dermis by incubating skin samples in 2 mg/mL Dispase II at 37° C for 2 hours. Following incubation, the epidermal sheets were carefully peeled off and digested with 0.1 mg/mL of DNase I at 37° C for 15 min shaking at 230 rpm to obtain single-cell suspensions for subsequent analysis. After epidermis separation, dermis tissue was finely minced with scissors and incubated at 37° C for 1 hour with shaking at 700 rpm on a thermoblock in digestion solution containing 2.7 mg/ml collagenase XI, 0.25 mg hyaluronidase, and 0.1mg/ml DNase I in RPMI with 1X HEPES. Digested tissue was then filtered through a 30 µm filter cone (CellTrics 30 µm Cat# 04-0042-2316, Sysmex) into a new tube for subsequent analysis. Mouse IEL were isolated from small intestine. Briefly, small intestine was opened, washed in PBS, cut into 0.5-cm long pieces and incubated at room temperature in complete RPMI supplemented with 1mM DTT. Tissues were then subjected to two rounds of washes with complete RPMI, vortex and filtered through 70µm cell strainers. IEL fraction was then purified by Percoll density gradient (80:40:20) centrifugation and stained by flow cytometry. To analyse γδ T cell populations, separate tissue-specific cell suspensions were prepared as described above from ears or back skin or gut of individual animals. γδ T cell were stained with specific antibodies.
Instrument	Data acquisition was performed using the LSR Fortessa (BD Biosciences) and the Spectral Aurora flow cytometer (Cytek Biosciences).
Software	Conventional flow cytometry data was recorded using FACSDiva Software v9.2 (BD) and spectral analysis flow cytometry using the Cytek SpectroFlow Software v3.3.0 (Cytek). All flow cytometry was analyzed using FlowJo v10 software (BD Biosciences).
Cell population abundance	All flow cytometry experiments were performed using single-cell suspensions. The abundance of each cell population is reported as a percentage of the parent population (e.g., live cells, $CD45^+$ cells), unless otherwise specified. Absolute cell numbers were calculated based on the output provided by the cell sorter. In experiments involving cell sorting, absolute numbers were derived from the sorter's event count and the volume collected per fraction. All sorted populations analyzed contained more than 25,000 events to ensure statistical robustness.
Gating strategy	Cells were first gated based on forward and side scatter to exclude debris and aggregates, followed by exclusion of dead cells using a viability dye. Lineage-specific markers were then used to identify subpopulations of interest. Full gating strategies, including representative plots and marker combinations, are provided.

☒ Tick this box to confirm that a figure exemplifying the gating strategy is provided in the Supplementary Information.
