# Supplementary material for: γδ T cell receptor dependencies define a unique immunosurveillance modality
Source: Nature. 2026 Sep 30;659(8140):490–501. doi: 10.1038/s41586-026-11076-4 (PMC13754573; doi:10.1038/s41586-026-11076-4)
Supplement: Supplementary file 3 [file 41586_2026_11076_MOESM5_ESM.pdf]

nature portfolio

Peer Review File

γδ T cell receptor dependencies define a unique immunosurveillance modality

Corresponding Author: Dr Miguel Munoz-Ruiz

Parts of this Peer Review File have been redacted as indicated to maintain the confidentiality of unpublished data.

Version 1:

Reviewer comments:

Referee #1

(Remarks to the Author)
A. It is frequently stated that gamma/delta (gd) T cells „bridge innate and adaptive immunity" because they express a T-cell receptor (TCR) like adaptive ab T cells but also several activating NK receptors, and can respond to cytokine activation (e.g., IL-1beta + IL-23) like ILC. This raises issue about the uniqueness of gd T cells as they share many features with various forms of ILCs. The current study rigorously addresses this "gd paradox" using highly innovative and unique approaches. Veland et al have generated Trgc1 floxed mice crossed to tamoxifen-dependent Cre recombinase mice under control of TCRd or Rosa26 promoter. Treatment of adult mice with tamoxifen for 7 days followed by 7 days of recovery induced acute deletion of certain TCRgd (those linked to Cg1, i.e. Vg4/5/6/7), with massive consequences for the gd compartment in tissues where those Vg TCRs predominate (i.e., intestine and skin) while systemic gd T cells expressing TCR of the g4 and cluster (Vg1) were preserved and partially compensated for the absent Vg's. This unique system allowed the authors to ask a key (and hitherto unsolved) fundamental question: What is the consequence of acute TCR deletion for the phenotype and function of gd T cells in the dermis/epidermis and intestine? The impressive results unequivocally demonstrate that continued TCR expression is vital for maintenance of gd T-cell phenotype and function. The results first show that TCR-ablated gd T cells do persist in dermis/epidermis for at least some time. Using a variety of approaches (spectral and conventional flow cytometry, microscopy, transcriptomics, ATACseq; in vivo assays like transepidermal water loss, ear thickening, tumor rejection) they analyzed the impact of TCR deletion. TCRgd-deleted gd T cells showed specific alterations of certain surface markers (increased expression of CD103, reduced expression of CD69, Tigit, Lag3) which were paralleled by transcriptomic changes. This revealed higher numbers of genes differentially regulated by TCRgd deletion in epidermis compared to dermis. Observations on consequences of TCRgd deletion were paralleled also in the gut. Again, phenotypic changes to some extent resembled those in the skin (increase of CD103, decrease of CD69, Tigit). Further studies revealed important consequences for immunosurveillance of TCRgd deletion. Ear thickening in response to Aldara (i.e., induction of IL-17 by IL-1beta+IL-23) was impaired despite the fact that TCRgd-deleted gd T cells produced IL-17 in vitro in response to IL-1beta+IL-23. Furthermore, TCRgd-deleted mice showed increased tumor formation upon injection of B16.F0 melanoma cells. Last but not least, a proof-of-principle experiment verified comparable consequences of acute TCR deletion in human virally transformed Vd1 gd T cells. Interestingly, a similar set of core genes seemed to be regulated by TCRgd deletion in human Vd1 T cells as in murine cells (e.g., TIGIT, XCL1).
B. This is a highly original study of high significance and relevance to the field. The results are of interest to a broader audience, far beyond gd T-cell aficionados.
C. Overall, this study is very well conducted and constitutes a very significant step forward to a more complete understanding of the role of the TCR for the functioning of gd T cells. The study very clearly separates gd T cells from innate cells like ILCs, NK cells etc. The conclusions and data interpretation are justified and valid.
D. Statistical considerations: In general, appropriate statistical methods have been applied. However, I don't think it's appropriate to calculate significances with the unpaired two-tailed t-test from only two experimental values (Fig. 1f, Suppl. Fig. 1i, right; Suppl. Fig. 1p, Suppl. Fig. 1s).
E. The overall conclusions are robust and valid.
F. Suggested improvements:
1. Fig. 1g: In this example, significant TCRd cells (15%) were still present. Which Vg element did these cells express?
2. Fig. 2e/g: In view of the effect of TCRgd deletion on CD103 expression: was there also an effect on the expression of the KLRG1 receptor which is expressed on TRM cells?
3. Figure 4, line 386: is there a specific reason why consequences of TCR deletion in the gut were analyzed 4 weeks after tamoxifen treatment as compared to earlier time points for the skin?

4. Figure 6/Suppl. Figure 6 and Methods Lines 54-90 (Human Vd1 cells): It is not clear from how many (if more than one) donors such HTLV1-immortalized cell lines have been established and analyzed, please specify. Moreover, the wildtype and Vd1-deleted cells should be characterized in more detail, specifically with respect to surface markers known to contribute to cytotoxic activity of Vd1/DOT cells (e.g., NKG2D, NKp30, NKp40, DNAM-1). In this context, it would be of interest to characterize the target cell used in Fig. 7g (HT29) for expression of relevant ligands.
G. References: are appropriate
H. Clarity: The abstract is clear and accessible to a broad audience. Similarly, introduction, discussion and conclusions are well written and can be followed also by non-specialists

Referee #2

(Remarks to the Author)
This study addresses a long-standing question that has beleaguered immunologists since the discovery of gamma delta (gd) T cells in the 1980s: namely, what is the role of the gdTCR(s) beyond regulating gdT cell development? The authors approach this question by generating an inducible gdTCR deleter mouse to profile tissue-resident cells in adult mice. The focus is mainly on epidermal Vg5 cells, dermal IL-17-producing Vg4/Vg6 cells, and intestinal Vg7 cells. The paper reports that the gdTCR is important for each gdT cell subset investigated, regulating their gene expression to different degrees. Most of the experiments were conducted in naïve, unchallenged mice. However, a psoriasis model and the B16 melanoma model were employed to test response after gdTCR loss, which showed that the gdTCR is necessary for functionality. A human cell line that expresses the Vd1 TCR was also used to validate gdTCR regulation of gene expression in human gdT cells.
The novelty of the study is high – not only because of the question answered but also because of the elegant, genetic approach in both mice and human cells. Validation of the mouse model is made very clear, walking the reader through the complexity of the gdTCR locus in an easy-to-understand way. Efforts were made to confirm specific deletion of Cgamma1 at various levels in a robust manner. The inclusion of mTmG mice was essential here. Another major takeaway from the data provided is the kinetics of Vg5, Vg4/Vg6, and Vg7 cell survival following TCR deletion, where skin Vg4/5/6 remain unaffected while Vg7 cells are lost. The weaknesses of manuscript include the lack of functional data specifically for the Vg5 and Vg7 subsets, and the fact that robustness is not maintained for every set of experiments. Specific points are outlined here:

1. Quantification is needed for every flow plot and set of images in the manuscript. For example, Figure 1c-h, 2g, 4e, 7a, Supp 7a, etc.
2. In some experiments (Figure 1f, 1i), only 2 mice are included. More replicates should be included.
3. Figure 4: Following gdTCR deletion, Vg7 cells are reduced. Do they undergo apoptosis? The FAS expression data in Vg7 cells is interesting but confusing. The authors suggest that FAS is a gut-retention factor for these cells. How can it be ruled out that the TCR rather than FAS is the retention factor for the cells? Do the same enterocytes expressing BTNL1/6 also express FASL?
4. The data in Figure 4b are not presented correctly. Each mouse should be shown as a unit rather than each villus and statistics should be calculated accordingly.
5. Can the authors confirm whether gene expression changes seen in Figure 6 using the human cell line also occur at the protein level? The co-stimulatory/inhibitory molecules and cytokines in particular are easy to measure.
6. How many replicates were performed for Figures 7a and Supp 7a? Please provide graph showing all replicates as done for Supp 7c.
7. The Aldara experiment shown in Figure 7b offers compelling data on the role of gdTCR in Vg4/6 cells, which drive pathology in this model system. However, the same is not provided for Vg5 or Vg7 cells to the same extent and these data are essential. The UVR model in Supp Figure 7f only shows that the gdTCR regulates phenotype – it does not show that Vg5 cell behavior has changed. The B16 melanoma transplanted subcutaneously does not provide the right model system to investigate Vg5 functionality, because Vg5 exist in the epidermis. There is no evidence that Vg5 cells penetrate subcutaneous B16 tumors. As such, I believe the data provide evidence that the gdTCR is important for CD27+Vg4+IFNg+ in controlling tumor growth, based on what we know about the gdT cells that infiltrate subcutaneous B16 tumors (Lanca et al., JI 2013; Lopes et al., Nature Immunology 2021). The Hayday lab has established the importance of Vg5 in cancer immunosurveillance using the carcinogenesis models (i.e. DMBA/TPA and MCA) where the biology of NKG2D signaling is well established (Strid data). Therefore, these models should be employed here. NKG2D expression should also be examined. Tumor penetrance of these models is best using FVB/n mice, but C57BL/6 mice can also be used. For Vg7 cells, the authors should cross mice with ApcMIN mice or transplant colon cancer cell lines orthotopically. Only when these orthotopic models are used can the authors draw conclusions about the gdTCR on tissue-resident Vg5 and Vg7 cells during tumor growth. Currently, the evidence from B16 model does not support the conclusions.
8. In Figure 7e, what does the Vg5 TCR recognize on B16 cells? Do B16 cells express SKINT1?
9. How many replicates were performed for Figure 7g?
10. Figure legends often lack information on sample size.

Referee #3

(Remarks to the Author)
This manuscript addresses the long-standing question of whether activation of the TCR is required for the functional responses among tissue resident γδ T cells. To this end, the authors generate and characterize a global Cγ1 KO mouse and different inducible reporter strains optimized to maximize recombination within the skin and IEL compartment. The success of this approached allowed for the characterization of tissue resident γδ IEL subsets Vγ4/5/7 in the absence of TCR activation. Ultimately, the authors find that TCRγδ signaling affects the transcriptional regulation and chromatin accessibility

of a small subset of genes, and that at steady-state, TCRγδ expression has a lesser impact on those cells that underwent Btnl-dependent, TCR-mediated repertoire selection. These findings complement and support previous work from this group regarding the contribution of Skint1 and Btnl1 in their respective compartments, while adding moderately to our understanding of the role of TCRγδ in dermal γδ T cell function. While this is an important conceptual study, the functional data provided convincingly support the requirement for acute TCRγδ activation in the skin yet fall short of accomplishing the same in the gut. Moreover, the overall rigor of the data could be improved by including more quantification and statistical analyses of flow cytometry-based assays, as this is absent for some experiments or lack sufficient statistical power.

Major Concerns:

1. The relative frequency of TCRγδ+ cells in γδx mice should be represented graphically corresponding to the MFI data shown in Fig S1g. The legend for Fig S1g is confusing: "7 days following tamoxifen treatment of γδTCR and γδx mice, one month after tamoxifen-induced TCRγδ deletion". Does this graph show two different timepoints? Based on these data showing that TCR expression can persist for a month after Cre induction and the relatively long half-life of a DETC, how long does it take for all TCRγδ to be deleted from all cells?

2. In Figure 4a, without reference staining, it is difficult to ascertain whether the majority of the GFP+ cells are located in the epithelium or lamina propria. Figure 4b should be shown with each dot representing an individual mouse as opposed to tissue section. Also, it would be helpful to see both representative flow plots and quantification of the frequency of Vγ subsets in the IEL compartment in the mTmGγδTCR and mTmGγδX mice relative to the Cγ1 KO shown in Figure S1d.

3. Cytokine production by combined dermal and epidermal cells following in vivo deletion of TCRγδ in Fig 7A seems to differ compared the two populations separated (with DETC TCRγδ deleted in vitro) in Figure S7a,b, yet this was not addressed or quantified clearly. The conclusion drawn in line 549, is that acute TCR deletion had "little consistent impact" whereas Fig S7b appears to have a substantial increase in IFNg production by stimulated DETCs and the opposite effect in γδ IELs. IFNg and IL-17 production should be shown (Fig S7a-c) from multiple mice (>2) and statistics performed. Although the potential of dermal γδ T cells to make IL-17 is not altered, this was not directly demonstrated in vivo in response to Aldara. Closing this loop would provide clearer support for these findings.

4. Is ear thickness also reduced following UVR in γδX mice relative to γδTCR similar to the prior findings with Skint1 antibody treatment?

5. Overall, there is a lack of statistical analyses as many figure panels only show representative data (1e,g, S1d,h, 2g, S2, 4e, S4b, 7a,f,g, S7a-c)

6. IEL data was presented, but unlike the skin, no experiments/perturbations were performed to show whether the TCR is required for their protective function in the gut. Based on the data presented, the authors' conclusions that 'active tissue surveillance depends on TCRγδ' applies to the skin, where this group and others have shown clear TCR dependence. However, this conclusion is less well supported in the context of the intraepithelial compartment.

7. Metabolic assays were only performed in immortalized blood cell line, not in tissue resident cells. The authors state that effects on cellular metabolism "likewise implied" based on the altered transcriptome for murine intestinal IEL and dermal γδ T cells, yet this was not directly tested. In particular, the potential involvement of TCRγδ in mediating a switch to glycolysis may not hold true for γδ IELs which uniquely exhibit linked glycolytic and mitochondrial dependency.

Minor Concerns:

1. Some flow plot axes read as "SCC" instead of "SSC" and the corresponding genotype of the mice needs to be labelled in the figure (Fig S1f).

2. In Fig S1d, it is surprising to see 40% Vγ4 staining in the WT IEL compartment. Is this gated on TCRγδ+ cells?

3. Previous work showed that Jun expression was decreased following antibody-mediated inhibition of TCR signaling (PMID: 39311642), yet surprisingly, this gene was upregulated in the γδX mice. Could the authors provide a potential explanation for the observed difference?

4. In Fig 3c, the gene count legend should be scaled appropriately to accurately reflect the number of genes in the pathways with the smaller circles.

5. In Fig S1m and 4a, it is extremely difficult to appreciate the positive staining shown in blue. Can the pseudocolored GFP/TCRab be displayed in a more visible color (i.e white or magenta)?

6. Figure legend Fig S5a,b: Please indicate the cell type.

7. Figure Legend 7e: The difference between 7d and 7e is unclear. Fig 7e indicates that mice were treated with tamoxifen but also that the TCR was deleted in vitro with 4-OHT. Are these two panels from the same experiment? Likewise, was the TCR deleted in vitro or in vivo in Fig 7f? Are there statistics for this experiment? There is also an inconsistency in the figure and figure legend for Fig. 7a regarding treatment duration.

8. The timepoints at which mice were euthanized following TCRγδ deletion should be clearly indicated in the figure legend for each experiment as this appears to vary between experiments.

9. Since γδ IELs die quite easily in ex vivo culture, were the cultures shown in Fig S7c maintained with IL-15? I would expect that the unstimulated ones would not fare well under these conditions.

Referee #4

(Remarks to the Author)
I co-reviewed this manuscript with one of the reviewers who provided the listed reports.

Version 2:

Reviewer comments:

Referee #1

(Remarks to the Author)
The authors are to be congratulated for their efforts to further improve the overall quality and conclusions of their study. My comments have been adequately addressed.

Referee #2

(Remarks to the Author)
As I said previously, this study has addressed a problem that has existed since the discovery of gdT cells in the 1980s, and we now have the answer. The authors have delivered on every request, which has substantially strengthened the conclusions of the study. The addition of three additional in vivo models (B16 epicutaneous challenge, DMBA/TPA, and AOM/DSS) along with previously provided model data provides the most convincing evidence that tissue-resident TCRs are required for tissue perturbation responses and for cancer immunosurveillance. Although the experiments took time, animal license adjustments, and a great deal of breeding, these data were worth the wait. I have no further concerns. I do have two suggestions to improve readability of figures: Figure 1G should include labels next to images on the horizontal (such as mouse 1, 2, 3, etc.), and Figure 7B would benefit from a label describing the difference between the upper (low cancer cell number) and lower (high cancer cell number) pie charts.

Referee #3

(Remarks to the Author)
The revised manuscript addresses many of the prior concerns regarding clarity and reproducibility of the findings by increasing sample size where applicable. The studies in the epidermis and dermis are rigorous and complete. The addition of the inflammation-associated carcinogenesis model in the gut provides a clear indication of a TCR-mediated role for γδ T cells in protection against tumorigenesis. However, additional discussion is warranted to provide a holistic interpretation these findings. This is particularly pertinent since both Vγ7 and Vγ1 IELs are anti-tumorigenic, whereas Vγ4 and Vγ6 have pro-tumor functions, and γδX mice would affect Vγ4/6/7 but not Vγ1 T cells. Further, all characterization of the γδX IEL compartment was performed in the small intestine, with no description of the colonic γδX T cells (IEL or LP) which would be responsible for the phenotypic changes in AOM/DSS model.

Specific Concerns:

1. Across all figures including cells isolated from the gut, please specify the region of the gut (SI vs colon), and based on the isolation method and Vγ usage, label as IELs throughout the manuscript.

2. For ATAC-seq data in the gut, CD45hi Dump neg cells were sequenced, were these cells also GFP+? If GFP+ cells were not sorted then it is presumed that are still Vγ1 IELs contained in these data. Fig 3d and Extended Fig 3d only shows the loss of Vγ7 and Vγ4. This could explain why there are fewer differences observed in the gut relative to the epidermis, which seems like an important caveat/limitation to note.

3. In Fig 7F, it would be helpful to label inflamed and dysplastic/tumor regions of the tissue. The representative data in Fig 7F do not appear to reflect tumor number shown in the right panel of Fig 7G.

Referee #4

(Remarks to the Author)
I co-reviewed this manuscript with one of the reviewers who provided the listed reports.

Open Access This Peer Review File is licensed under a Creative Commons Attribution 4.0 International License, which permits use, sharing, adaptation, distribution and reproduction in any medium or format, as long as you give appropriate credit to the original author(s) and the source, provide a link to the Creative Commons license, and indicate if changes were made.
In cases where reviewers are anonymous, credit should be given to 'Anonymous Referee' and the source.
The images or other third party material in this Peer Review File are included in the article's Creative Commons license, unless indicated otherwise in a credit line to the material. If material is not included in the article's Creative Commons license and your intended use is not permitted by statutory regulation or exceeds the permitted use, you will need to obtain permission directly from the copyright holder.
To view a copy of this license, visit https://creativecommons.org/licenses/by/4.0/

Referee expertise:

Referee #1: γδ T cell biology, cancer
Referee #2: γδ T cell biology, cancer
Referee #3 & #4: γδ T cell biology, barrier function, mucosa
Referees' comments:

Response to the Reviewers.

Prior to our point-by-point response, we apologise to the reviewers for the time taken for the revision. However, in our defence, we note two points.

1. Some of the reviewers' comments (with which we wholly agreed) requested we broaden our analysis of the impact of TCR deletion on immunosurveillance in vivo. This we have achieved, introducing three new models, including one in the gut. These models take many, many months for outcome measures, specifically tumours and inflammatory lesions, to develop, and they can take weeks to thoroughly analyse, specifically by histopathology. Moreover, in order to undertake them, we required amendments to our government-issued animal licence that took months to be approved. And, added to this, we needed to run pilot dose-escalation experiments to determine the appropriate concentrations of cells, carcinogens and inflammatory agents to use in our current animal facility. Happily, all these steps proceeded without any missteps, but they took time.
2. We have additionally repeated and expanded several of the experiments presented in the original submission, e.g., Aldara challenge. While those experiments are of themselves relatively short duration, they require breeding programs that deliver the appropriate cohorts of experimental and control mice. Occasionally there are losses of litters, etc., and the duration of the work is inevitably extended.

   Overall, we are very pleased with the outcomes of all these experiments and thank the reviewers for suggesting them.

   Additionally, we have rearranged the data somewhat, specifically by: [a] reducing the size of Extended Data Fig 1 via the use of a Supplementary data set for the pilot studies with the constitutive indel strain; [b] by condensing Figs 2 and 3 into one figure (new Fig 2). This has allowed us to use two figures (Fig 6 and Fig 7) to present the much-expanded immunosurveillance data. We have deleted some data, e.g. NanoString, that seemed no longer important given all the other datasets that we present.

   Finally, we are grateful to the reviewers for their emphasising the importance of this study in addressing a long-standing and fundamental question in immunology: namely, do γδ T cells post-development genuinely exist as purely innate cells, akin to ILC and NK cells, or do they continue to require their TCR throughout? Some suggestion of the latter derived from our earlier studies in which we acutely knocked down Btnl1 in the gut or antibody-blockaded Skint1 in the skin. However, those experiments did not preclude a TCR-independent role for those molecules, and they did not address the question for the many γδ T cells that are not BTNL-selected.

Moreover, as we consider in the Discussion, the impact of TCR deletion on BTNL-selected Vγ7 IEL exceeds the impact of BTNL-deletion, arguing that even in this instance, the TCR on mature cells is being signalled in a BTNL-independent manner. In short, these are novel and unanticipated findings. Moreover, they seem germane to another issue, which is the widespread conservation of γδ T cells across gnathostomes. Given that no analysis has yet found an unique feature of γδ T cells beyond TCRγδ, it is likely that the TCR itself has been the primary target of natural selection, in keeping with an overarching role in γδ T cell biology.

Point-by-point response

Referee #1

We thank the reviewer for their thorough evaluation and constructive suggestions of our manuscript and for their positive assessment of its originality, rigor, and significance. We appreciate the recognition that our work addresses the longstanding "γδ T-cell paradox" using innovative approaches and that the conclusions are considered robust and broadly relevant.

D. Statistical considerations: In general, appropriate statistical methods have been applied. However, I don't think it's appropriate to calculate significances with the unpaired two-tailed t-test from only two experimental values (Fig. 1f, Suppl. Fig. 1i, right; Suppl. Fig. 1p, Suppl. Fig. 1s).

We agree and apologise for this oversight. There is no longer an example of this, primarily as a result of multiple repeats of several experiments, thereby increasing "n". In no case did the repeat experiments, provide contrasting data, and the study's conclusions have consequently been strengthened. The additional studies are reflected in the updated figures and legends, and specific instances are also cited in response to other reviewers' comments, below.

F. Suggested improvements:

1. Fig. 1g: In this example, significant TCRd cells (15%) were still present. Which Vg element did these cells express?

We have now clarified this issue in the revised paper, particularly in Figure 1b. The percentage of these cells varied somewhat from mouse to mouse but was on average ~15% of $CD45^{high}$ that are $V\gamma5^{+}$ $17D1^{+}$ (Fig1b). In sum, these cells seem to be typical DETC that simply escaped complete deletion in $\gamma\delta^{X}$ mice. In the text: "The remaining γδ cells retained reactivity to 17D1 an antibody specific at steady-state for the $V\gamma5^{+}$ DETC TCR". These cells remained a small minority of epidermal lymphocytes across the experimental time spans examined.

2. Fig. 2e/g: In view of the effect of TCRgd deletion on CD103 expression: was there also an effect on the expression of the KLRG1 receptor which is expressed on TRM cells?

For whatever reason, KLRG1 is not expressed on typical WT DETC, and it is unaffected by acute TCR ablation (see figure below). We have not included this figure in the manuscript and provide it here only for the reviewer's reference.

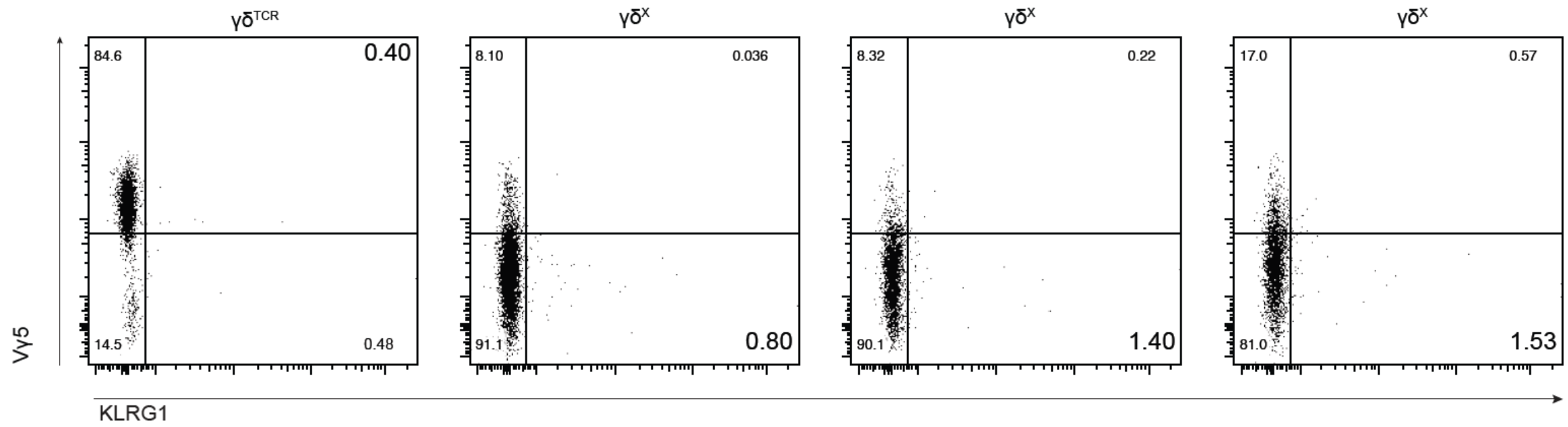

3. Figure 4, line 386: is there a specific reason why consequences of TCR deletion in the gut were analyzed 4 weeks after tamoxifen treatment as compared to earlier time points for the skin?

We have now expanded our analysis of acute TCR deletion in the gut to 2 weeks as well as 4 weeks after tamoxifen induction. In both cases there were significant decreases in the numbers of cells per villi across all $\gamma\delta^{X}$ mice examined when compared with controls (shown now in Figure 3a, 3b, 3d; Extended Data Figure 3a-c). Note too that our analysis of this issue has been substantially extended (see response to Referee 3), and includes micrographic and flow cytometry data, all of which were consistent with one another.

4. Figure 6/Suppl. Figure 6 and Methods Lines 54-90 (Human Vd1 cells): It is not clear from how many (if more than one) donors such HTLV1-immortalized cell lines have been established and analyzed, please specify.

For this study, the parental line was derived from a single donor, as in now clarified in the Methods section.

Moreover, the wildtype and Vd1-deleted cells should be characterized in more detail, specifically with respect to surface markers known to contribute to cytotoxic activity of Vd1/DOT cells (e.g., NKG2D, NKp30, NKp40, DNAM-1). In this context, it would be of interest to characterize the target cell used in Fig. 7g (HT29) for expression of relevant ligands.

We have undertaken these experiments using flow cytometry and they are presented in Figure 5b, 5c and Extended Data Figure 7i. The results are fully consistent with the TCR regulating the expression of NKRs, as previously implied by transcriptomics, and with the availability of NKR ligands being insufficient to support optimal targeting of tumour cells by Vδ1 cells from which the TCR was deleted.

<u>Referee #2 (Remarks to the Author):</u>

We thank the reviewer for their detailed evaluation of our manuscript. We appreciate the recognition of the conceptual importance of a question that has "beleaguered

immunologists since the discovery of gamma delta (γδ) T cells in the 1980s", and the novelty of the inducible genetic approach. We are also grateful for the constructive suggestions, which have helped us to significantly strengthen the manuscript, particularly with respect to functional validation.

Specific points are outlined here:

1. Quantification is needed for every flow plot and set of images in the manuscript. For example, Figure 1c-h, 2g, 4e, 7a, Supp 7a, etc.

We agree and apologise for this oversight. We have by now added the quantifications for all relevant figures.

2. In some experiments (Figure 1f, 1i), only 2 mice are included. More replicates should be included.

We apologise for this too and have increased the number of biological replicates for these experiments, now reflected in the new updated figures.

3. Figure 4: Following gdTCR deletion, Vg7 cells are reduced. Do they undergo apoptosis?

We have now assessed apoptosis and do not observe a difference in between genotypes. Extended Data Figure 3f.

The FAS expression data in Vg7 cells is interesting but confusing. The authors suggest that FAS is a gut-retention factor for these cells. How can it be ruled out that the TCR rather than FAS is the retention factor for the cells? Do the same enterocytes expressing BTNL1/6 also express FASL?

When we repeatedly observed the loss of gut γδ IEL from $γδ^{X}$ mice, we were reminded that FAS-deficient mice also could not retain typical γδ IEL in the intestine [PMID: 7540652] and therefore wondered whether there might be a connection. Indeed, data in Extended Figure 3g show that TCR deletion results in a downregulation of FAS in $γδ^{X}$ compared with $γδ^{TCR}$ gut IEL. This is, as the referee states, an interesting observation, and so we should like to retain it in the paper. Nonetheless, we completely agree with the referee that this does not necessarily mean that FAS is a retention factor rather than its expression being merely a marker for retention. We have amended the text accordingly and have similarly decreased the emphasis on this point. [redacted]

[Figure Redacted]

4. The data in Figure 4b are not presented correctly. Each mouse should be shown as a unit rather than each villus and statistics should be calculated accordingly.

We now present data from a new experiment that included 5 $\gamma\delta^{X}$ and 5 $\gamma\delta^{TCR}$ mice: in Figure 3a and 3b these data are represented by average GFP^{+} cells / villus together with the average number of GFP^{+} cells / villus for each of the 10 animals. In both cases, there is a statistically significant decrease in the cells in $\gamma\delta^{X}$ mice.

5. Can the authors confirm whether gene expression changes seen in Figure 6 using the human cell line also occur at the protein level? The co-stimulatory/inhibitory molecules and cytokines in particular are easy to measure.

We acknowledge the value of this point and now present surface protein quantification data for TIGIT, 4-1BB, TIM-3 and NKRs in Figure 5b, 5c and Extended Data Figure 5g. We present cytokine data in Extended Data Figure 6d.

6. How many replicates were performed for Figures 7a and Supp 7a? Please provide graph showing all replicates as done for Supp 7c.

These experiments are now shown in Figure 6a and Extended Data Figure 6a-c; for biological replicates, n = 3 for Figure 6a and n = 4-6 for the Extended Data Figure 6a-c. Examples of the primary data read-outs are shown together with the graphs.

7. The Aldara experiment shown in Figure 7b offers compelling data on the role of gdTCR in Vg4/6 cells, which drive pathology in this model system. However, the same is not provided for Vg5 or Vg7 cells to the same extent and these data are essential. The UVR model in Supp Figure 7f only shows that the gdTCR regulates phenotype – it does not show that Vg5 cell behavior has changed. The B16 melanoma transplanted subcutaneously does not provide the right model system to investigate Vg5 functionality, because Vg5 exist in the epidermis. There is no evidence that Vg5 cells penetrate

subcutaneous B16 tumors. As such, I believe the data provide evidence that the gdTCR is important for CD27+Vg4+IFNg+ in controlling tumor growth, based on what we know about the gdT cells that infiltrate subcutaneous B16 tumors (Lanca et al., JI 2013; Lopes et al., Nature Immunology 2021). The Hayday lab has established the importance of Vg5 in cancer immunosurveillance using the carcinogenesis models (i.e. DMBA/TPA and MCA) where the biology of NKG2D signaling is well established (Strid data). Therefore, these models should be employed here. NKG2D expression should also be examined. Tumor penetrance of these models is best using FVB/n mice, but C57BL/6 mice can also be used. For Vg7 cells, the authors should cross mice with ApcMIN mice or transplant colon cancer cell lines orthotopically. Only when these orthotopic models are used can the authors draw conclusions about the gdTCR on tissue-resident Vg5 and Vg7 cells during tumor growth. Currently, the evidence from B16 model does not support the conclusions.

We completely understand the reviewer's concern and consequently have substantially expanded the experimental systems in which we have assayed the impact of TCRγδ deletion. This has taken many months because in some cases, the studies were no longer covered by our animal licence, requiring us to obtain new approvals. We would make the following points:

a. We retain the B16 model used in the original submission because it is so widely used, because it is a stringent test of immunosurveillance, and because γδ T cell-deficient mice were shown to have increased susceptibility.
b. We have additionally employed B16 cells in an epicutaneous challenge model that was specifically developed to test the cancer immunosurveillance responses of epidermal tissue-resident T cells [PMID: 30598548].
c. We have employed the DMBA-TPA model as the reviewer requested. This model takes longer to run in B6 mice than in FVB mice in which we originally did all our formative studies. Nonetheless, even in B6 mice, it is well-suited to testing the impact of epithelial immunosurveillance on carcinogenesis and its inflammatory component.
d. We have employed the gut equivalent of the DMBA-TPA model, namely the AOM-DSS model, that likewise assays susceptibility to carcinogenesis and inflammatory lesions. This model is widely used in the γδ T cell community and has been shown to be more severe in mice lacking γδ T cells and in particular, gut γδ IEL [PMID: 33989816; PMID: 35857588].

In every model, a non-redundant role for TCRγδ was evident as presented in Figure 7 and Extended Data Figure 7.

8. In Figure 7e, what does the Vg5 TCR recognize on B16 cells? Do B16 cells express SKINT1?

We do not know what TCRγδ is recognising in each setting where its biological activity is evident. This is considered at some length in the Discussion and clearly is the next bridge to cross. Meanwhile, we confirm that B16 cells do not express SKINT1; see figure below comparing CD45(-) skin cells with B16 cells. We have not included this figure in the manuscript, providing it here only for the reviewer's reference.

Skint1 mRNA (dCT)

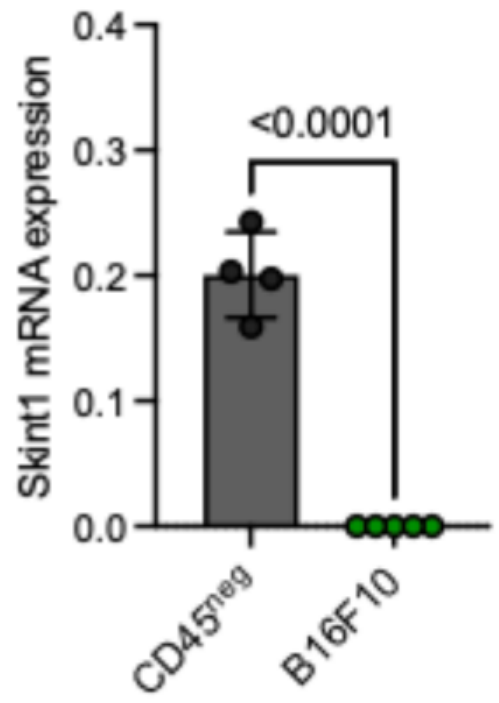

9. How many replicates were performed for Figure 7g?

We have now applied the IncuCyte system in 3-5 independent experiments to three independent cell lines: Figure 7h and Extended Data Figure 7h. For HCT116, n = 5; for HT29, n = 4 and for A375, n = 3.

10. Figure legends often lack information on sample size.

We have provided this information on the figure legends.

Referee #3 (Remarks to the Author):

We thank the reviewer for their careful and constructive evaluation of our manuscript. We are pleased that they recognise the conceptual importance of addressing whether TCR activation is required for the functional responses of tissue-resident γδ T cells, as well as the strength of our genetic models and the insights gained into epidermal and intestinal γδ T cell biology. We appreciate their acknowledgement that our findings support and extend previous work from our group on Skint1- and Btnl1-dependent selection pathways. These comments were helpful and helped us in strengthening the manuscript.

Major Concerns:
1. The relative frequency of TCRγδ$^+$ cells in γδ^x mice should be represented graphically corresponding to the MFI data shown in Fig S1g.

To address this point, we have now included in Extended Data Figure 1c a graph showing the frequencies of TCRγδ$^+$ cells and their TCRδ MFI comparing the two genotypes.

The legend for Fig S1g is confusing: “7 days following tamoxifen treatment of γδTCR and γδx mice, one month after tamoxifen-induced TCRγδ deletion”. Does this graph show two different timepoints?

We apologise; the legend was mistaken and has now been corrected.

Based on these data showing that TCR expression can persist for a month after Cre induction and the relatively long half-life of a DETC, how long does it take for all TCRγδ to be deleted from all cells?

For the Tcrd$^{Cre\text{-}ERT2}$ model, our data suggest a penetrance in the skin of ~85-90%, although there is mouse to mouse variation, as would be predicted. For the most part, the residual TCR^{+} cells are DETC that escaped deletion. Although we have not sampled skin from a single animal longitudinally, the data we have suggest that the population is sustained at approximately the same percentage over at least a 2 -3-month period post tamoxifen treatment, indicating that the TCR^{+} cells in vivo have neither a growth advantage nor disadvantage relative to TCR-deleted cells (2-months post-tamoxifen data are included in Figure 1f). Possibly more complete TCR deletion could be achieved with successive tamoxifen treatments, but this would introduce complications to the experimental design and, importantly, was clearly not necessary to observe [a] the major effects that the loss of the TCR had on most γδ cells in each anatomical compartment and [b] the consequent impacts on immunosurveillance. This point is considered in the Discussion.

2. In Figure 4a, without reference staining, it is difficult to ascertain whether the majority of the GFP+ cells are located in the epithelium or lamina propria.

We acknowledge this point and have addressed it. First, to gain an optimal assessment in situ of the representation of cells in the intestine, our GFP^{+} cell counts were performed using 80 µm sections where it is challenging to assign cells to the epithelium or the lamina propria, in part owing to variability in the plane of section. We have included additional examples of these data to add strength to our cell quantitation. Additionally, we have now included a reference staining using tdTomato from the same mouse line (mTmG), using thinner cryosections (20 µm) that permit the epithelial layer to be more clearly visualized. As expected, the majority of GFP^{+} cells are IELs (Extended Data Figure 3c). Moreover, the overt depletion of IEL was validated by applying flow cytometry to an established protocol that reliably enriches for IEL (Figure 3c).

Figure 4b should be shown with each dot representing an individual mouse as opposed to tissue section.

We now present data from a new experiment that included 5 γδ^{X} and 5 γδ^{TCR} mice: in Figure 3a and 3b these data are represented by average GFP^{+} cells / villus together with the average number of GFP^{+} cells / villus for each of the 10 animals. In both cases, there is a statistically significant decrease in the cells in γδ^{X} mice.

Also, it would be helpful to see both representative flow plots and quantification of the frequency of Vγ subsets in the IEL compartment in the mTmGγδTCR and mTmGγδX mice relative to the Cγ1 KO shown in Figure S1d.

We agree and have addressed this point in Figure 3c.

3. Cytokine production by combined dermal and epidermal cells following in vivo deletion of TCRγδ in Fig 7A seems to differ compared the two populations separated (with

DETC TCRγδ deleted in vitro) in Figure S7a, b, yet this was not addressed or quantified clearly.

We apologise for the lack of quantitation which is now readdressed in Figure 6a and Extended Data Figure 6a, 6b, 6c. For Extended Data Fig 6b, the deletion of the TCR *in vitro* is performed in short-term DETC cultures which are less recalcitrant to IFNγ production than DETC directly *ex vivo*.

The conclusion drawn in line 549, is that acute TCR deletion had "little consistent impact" whereas Fig S7b appears to have a substantial increase in IFNg production by stimulated DETCs and the opposite effect in γδ IELs.

We completely understand the reviewer's perspective. Eliciting cytokines from skin γδ cells is variable from mouse to mouse and experiment to experiment, and across experiments the differences in the % of cells staining for intracellular cytokines was not significantly different across genotypes. However, we did note a significant increase in the amount of IFNγ detected per cell in stimulated DETC and this is now commented on (Extended Data Figure 6b).

IFNg and IL-17 production should be shown (Fig S7a-c) from multiple mice (>2) and statistics performed.

We have addressed this point: n = 6 in Extended Data Figure 6a; n = 3-5 in Extended Data Figure 6b, and n = 4 in Extended Data Figure 6c.

Although the potential of dermal γδ T cells to make IL-17 is not altered, this was not directly demonstrated in vivo in response to Aldara. Closing this loop would provide clearer support for these findings.

We are extremely grateful to the reviewer for raising this very important point. Specifically, it is well established that following Aldara challenge of WT mice, the rapidly expanding skin-draining lymph nodes (dLN) harbour activated, dermis-derived γδ T cells producing IL17 [PMID: 30455268]. Strikingly, this does not happen in $\gamma\delta^{X}$ mice (Figure 6d); the γδ T cells that are present are massively diminished in their production of IL17. However, this is not because the cells are not competent to produce IL17 because, consistent with the in vitro data shown in Fig 6a and Extended Data Fig 6a, the dLN cells produce IL17 when stimulated with either IL-1 + IL-23 or with PMA + ionomycin (Figure 6e). Thus, the overarching role of TCR γδ is seen *in vivo*, where it is evidently critical for relieving a block on effector function, even when that effector activation is induced by innate stimuli. This major point is commented on in the Discussion.

4. Is ear thickness also reduced following UVR in γδX mice relative to γδTCR similar to the prior findings with Skint1 antibody treatment?

Following UVR, TCR-ablated mice did not phenocopy the alteration in ear thickness that was observed in Skint1-blockaded mice [PMID: 35165446].

5. Overall, there is a lack of statistical analyses as many figure panels only show representative data (1e, g, S1d, h, 2g, S2, 4e, S4b, 7a, f,g, S7a-c.

We apologise: we have by now increased n values across the board; have quantified the data; and provided statistical analyses. Throughout we combine the presentation of representative raw data (e.g., the flow plots) with bar graphs that aggregate data from multiple independent samples.

6. IEL data was presented, but unlike the skin, no experiments/perturbations were performed to show whether the TCR is required for their protective function in the gut. Based on the data presented, the authors' conclusions that 'active tissue surveillance depends on TCRγδ' applies to the skin, where this group and others have shown clear TCR dependence. However, this conclusion is less well supported in the context of the intraepithelial compartment.

We agree that this was an important gap to fill, and we have done so by use of the AOM-DSS model that enables us to score the impact of TCR ablation on the development of inflammatory lesions and carcinogenesis. The model has been applied in several studies of γδ T cells with conclusions being drawn that a protective role is played by γδ T cells, particularly those in the epithelium [PMID: 33989816; PMID: 35857588]. We now show that this protective role is substantially diminished when the TCR is ablated (Figure 7f, 7g; Extended Data Figure 7f, 7g), further strengthening our conclusion that TCRγδ is required in real time for optimal γδ T cell-mediated immunosurveillance.

7. Metabolic assays were only performed in immortalized blood cell line, not in tissue resident cells. The authors state that effects on cellular metabolism "likewise implied" based on the altered transcriptome for murine intestinal IEL and dermal γδ T cells, yet this was not directly tested. In particular, the potential involvement of TCRγδ in mediating a switch to glycolysis may not hold true for γδ IELs which uniquely exhibit linked glycolytic and mitochondrial dependency.

We acknowledge this point. We note that following acute TCR ablation, genes that contribute to metabolic regulation are some of the most differentially expressed genes in dermal cells, in gut cells and in human cells. The human cells offered the opportunity to assess the functional implications of this and those data are presented. We were, however, inappropriate in assuming that similar functional impacts would directly extend to the other cell types and reflecting this, have modified the text. Meanwhile, we have performed Seahorse experiments using Flow-sorted gut IEL from $\gamma\delta^{TCR}$ and $\gamma\delta^{X}$ mice and, as the reviewer notes, the cells show conspicuously low metabolic rates. To this point, we have not detected differences across the genotypes, but we would note that our input cell numbers are severely limited by the synchronous availability of age- and sex-matched mice of the two genotypes. Thus, we believe that assessing the full impact of TCR ablation on the regulation of T cell metabolism at steady-state and following challenge should form a central thread of ongoing and future studies that will require a substantially expanded breeding programme feeding into several independent assay systems.

Minor Concerns:

1. Some flow plot axes read as “SCC” instead of “SSC” and the corresponding genotype of the mice needs to be labelled in the figure (Fig S1f).

We apologise and have now corrected this.

2. In Fig S1d, it is surprising to see 40% Vγ4 staining in the WT IEL compartment. Is this gated on TCRγδ+ cells?

Yes, the gating was on γδ T cells. We do see this occasionally, but the reviewer is correct in inferring that this is not typical as is evident from the updated version of Supplementary material Figure 1d.

3. Previous work showed that Jun expression was decreased following antibody-mediated inhibition of TCR signaling (PMID: 39311642), yet surprisingly, this gene was upregulated in the γδX mice. Could the authors provide a potential explanation for the observed difference?

It can be quite challenging to provide explanations for apparent differences when different experimental approaches were employed. However, we have reviewed the publicly available data in relation to the paper cited [PMID: 39311642] and note an expected inverse correlation (data included for the benefit of the reviewers). Specifically, while there is, as the referee points out, a divergent result for the expression of *Jun* RNA (panel A), there is in Fischer *et al*, an upregulation of *Lag3, Tigit, Pdcd1,* and *Ctla4* (panel B) RNA. Since these are all markers of T cell activation, we would expect them to be suppressed in the TCR-ablated cells which is precisely what we observe (panel B). We note that the regulation of T cells *in vivo* is complicated, and that inhibition may oftentimes be preceded by transient activation: that might indeed explain the respective patterns observed in the two studies, but this is of course mere conjecture.

Comparison of data from Fischer *et al*. 2024

A

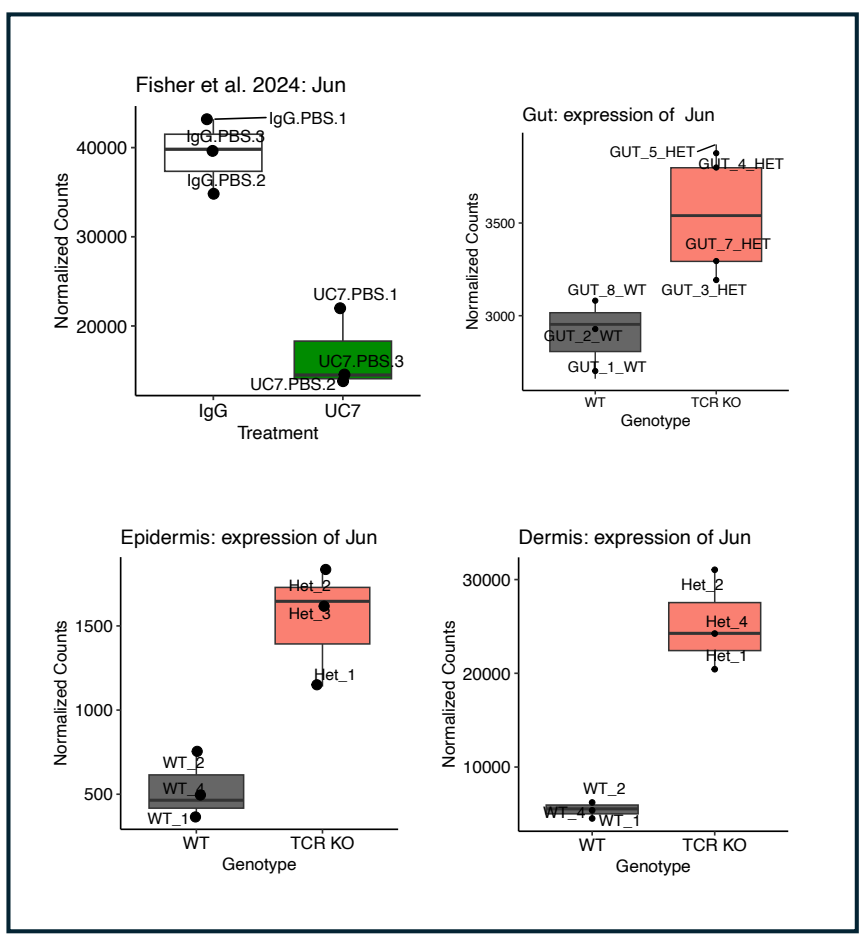

B

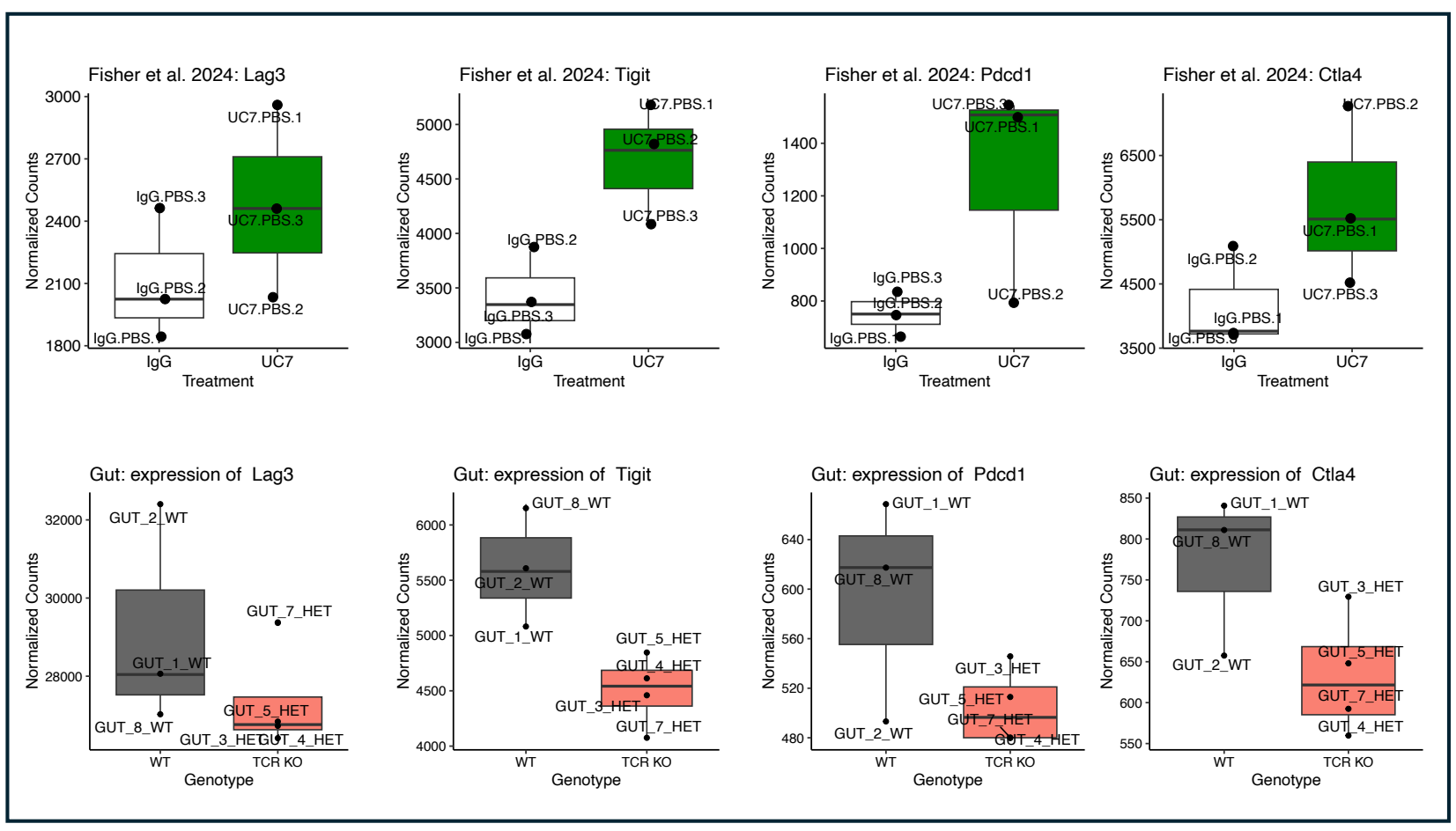

Fischer *et al*. open boxes = IgG control; green boxes = anti-TCRγδ (UC7)
Veland *et al*. grey boxes = $\gamma\delta^{TCR}$; pink boxes = $\gamma\delta^{X}$ (note, HET is an in-house nomenclature)

4. In Fig 3c, the gene count legend should be scaled appropriately to accurately reflect the number of genes in the pathways with the smaller circles.

We agree; please see new Extended Data Figure 2e.

5. In Fig S1m and 4a, it is extremely difficult to appreciate the positive staining shown in blue. Can the pseudocolored GFP/TCRab be displayed in a more visible color (i.e white or magenta)?

We agree. Moreover, we don't anymore believe that TCRαβ adds much for the purpose of this figure and have modified our approach to show GFP^+ cells and tdTomato as a reference stain (Extended Data Figure 3c).

6. Figure legend Fig S5a,b: Please indicate the cell type.

We have added this into the Figure legend.

7. Figure Legend 7e: The difference between 7d and 7e is unclear. Fig 7e indicates that mice were treated with tamoxifen but also that the TCR was deleted in vitro with 4-OHT. Are these two panels from the same experiment?

We apologise for the lack of clarity. The difference between those figures was that deletion in Figure 7d occurred *in vivo* after tamoxifen injection, while deletion in Figure 7e occurred *in vitro* after 4-OHT application. Results following deletion *in vivo* are now shown in Extended Data Figure 7c and results following deletion *in vitro* are now shown in Extended Data Figure 7d.

Likewise, was the TCR deleted in vitro or in vivo in Fig 7f? Are there statistics for this experiment? There is also an inconsistency in the figure and figure legend for Fig. 7a regarding treatment duration.

As above, the results following deletion *in vitro* are now shown in Extended data Figure 7d.

8. The timepoints at which mice were euthanized following TCRγδ deletion should be clearly indicated in the figure legend for each experiment as this appears to vary between experiments.

We apologise: this has now been updated in all the figure legends.

9. Since γδ IELs die quite easily in ex vivo culture, were the cultures shown in Fig S7c maintained with IL-15? I would expect that the unstimulated ones would not fare well under these conditions.

We completely agree with the reviewer: all experiments now shown included IL-15 during the entire *ex vivo* IEL culture (Extended Data Figure 6c).

Referee #4 (Remarks to the Author):

I co-reviewed this manuscript with one of the reviewers who provided the listed reports.

We thank you for your commitment to reviewing and improving our study.

We thank the referee for their sustained interest in the study and their suggestions for improvement.
With regard to the specific points they raise:

1. However, additional discussion is warranted to provide a holistic interpretation these findings. This is particularly pertinent since both Vγ7 and Vγ1 IELs are anti-tumorigenic, whereas Vγ4 and Vγ6 have pro-tumor functions, and γδX mice would affect Vγ4/6/7 but not Vγ1 T cells. Further, all characterization of the γδX IEL compartment was performed in the small intestine, with no description of the colonic γδX T cells (IEL or LP) which would be responsible for the phenotypic changes in AOM/DSS model.

With due respect for this point, one of our goals has been to substantially reduce the size of the Discussion so that it focuses very clearly on core issues informed by this study, specifically, innate versus adaptive immunity, gamma delta T cell classification, the implications for immunosurveillance, and the questions raised about the role of the TCR. Thus, with the space available, we do not believe that we can prioritise speculation concerning specific subsets of gamma delta T cells, particularly since classifications such as pro-tumour and anti-tumour may prove to under-estimate the pleiotropic functions of particular V-gamma-expressing subsets, particularly over the extensive time course of the gut carcinogenesis experiments. At this point, the data show unequivocally a dependence on the TCR in all immunosurveillance systems examined including the gut. Most certainly, later studies can probe how discrete subsets, including Lamina Propria cells and colonic cells are specifically affected by acute TCR deletion, but those are beyond the scope of this study which is already very extensive.

2. Across all figures including cells isolated from the gut, please specify the region of the gut (SI vs colon), and based on the isolation method and Vγ usage, label as IELs throughout the manuscript.

We thank the referee for their suggestion and have revised the text of the manuscript accordingly to clarify the sources of all cell types being examined in each displayed dataset. We believe that this is a preferable to adding additional clutter to the figures themselves. The effectiveness of our methods for the isolation and characterisation of IEL has been validated by the results of studies that we have conducted over the past several decades.

3. For ATAC-seq data in the gut, CD45hi Dump neg cells were sequenced, were these cells also GFP+? If GFP+ cells were not sorted then it is presumed that are still Vγ1 IELs contained in these data. Fig 3d and Extended Fig 3d only shows the loss of Vγ7 and Vγ4. This could explain why there are fewer differences observed in the gut relative to the epidermis, which seems like an important caveat/limitation to note.

We thank the reviewer for raising this point and have now clarified in the paper that the dump channel included an antibody against V-gamma-1 and therefore those cells would have mostly been removed.

4. In Fig 7F, it would be helpful to label inflamed and dysplastic/tumor regions of the tissue. The representative data in Fig 7F do not appear to reflect tumor number shown in the right panel of Fig 7G.

We thank the reviewer for this suggestion. We have added annotation to denote the areas of interest in [new] Fig 5 (formerly Fig7). The histology in this figure is provided primarily to illustrate the type of lesions that develop, and this is aided by the suggested addition of annotation. The polyp counts relate to the images shown in the Extended data fig 8g [formerly Extended data fig 7g) that accompanies Fig 5 [formerly Fig 7].
